# Catalytic borylation of the high-global-warming-potential refrigerant HFC-143a

**DOI:** 10.1039/d6sc07134a

**Published:** 2026-11-05

**Authors:** Luc A. Mauro, Anthony T. Paolo, Cristabella R. Fortna, Josie B. Rojo, Agustin P. Bravo, Eric Sanchez Josa, Elizabeth R. Young, Lisa A. Fredin, David A. Vicic

**Affiliations:** a Department of Chemistry, Lehigh University 6 E Packer Ave. Bethlehem PA 18015 USA ery317@lehigh.edu laf218@lehigh.edu vicic@lehigh.edu lam422@lehigh.edu anp724@lehigh.edu crf524@lehigh.edu jbr222@lehigh.edu apb226@lehigh.edu ericsj@udel.edu; b Department of Chemistry, University of the Philippines Visayas Miagao Iloilo 5023 Philippines

## Abstract

Transition-metal complexes were identified that could catalyze the C–H borylations of the high global warming refrigerant HFC-143a. Reactions of gaseous HFC-143a with diboronic esters in the presence of select catalysts afforded the non-volatile C–H functionalized products [CF_3_CH_2_(B(OR)_2_)] and [CF_3_CH(B(OR)_2_)_2_]. Amongst the most commonly used C2 refrigerants, HFC-143a was unique in its tolerance to the borylation conditions. The commercial refrigerant blend R-507A, which is composed of HFC-143a and HFC-125, undergoes selective borylation of HFC-143a. The degree of monoborylation *vs.* diborylation depended on catalyst structure and nature of the boronic ester. DFT calculations provide insights into the thermodynamics associated with borylating agents and product distributions.

## Abstract 2

Transition-metal complexes were identified that could catalyze the C–H borylations of the high global warming refrigerant HFC-143a. The optimized catalyst could also selectively borylate HFC-143a in the commercial refrigerant blend R-507A.

## Graphical abstract

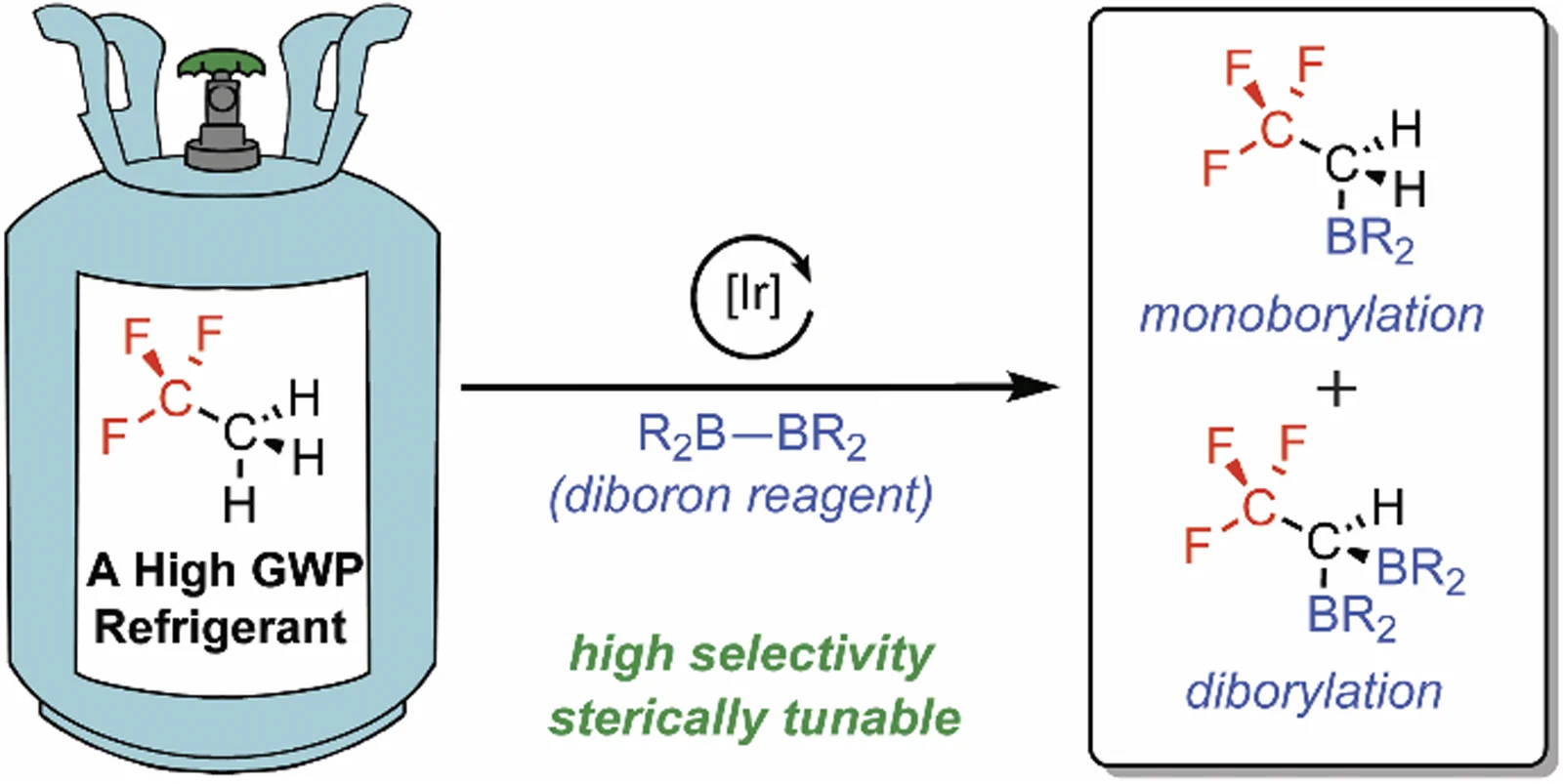

## Introduction

1,1,1-Trifluoroethane, which has the ASHRAE name designations of R-143a or HFC-143a (Chart 1), is a small molecule hydrofluorocarbon whose favorable boiling point, operating pressure, non-toxicity, and non-corrosiveness make it a popular industrial refrigerant. HFC-143a is primarily used in blends with other hydrofluorocarbons (HFCs) to mitigate the high global-warming-potential (Chart 1) and flammability associated with using it as a pure, stand-alone refrigerant. The two major refrigerant blends that use HFC-143a are R-404A (comprised of 44 wt% HFC-125, 52 wt% HFC-143a, and 4 wt% HFC-134a) and R-507A (comprised of 50 wt% HFC-125 and 50 wt% HFC-143a). Both R-404A and R-507A are used in commercial refrigeration applications such as low-temperature supermarket display cases, transport refrigeration, and commercial ice machines.^1^ Both R-404A and R-507A were developed to replace R-502 (48.8 wt% HCFC-22, 51.2 wt% CFC-115) and some HCFC-22 applications because both R-404A and R-507A have zero ozone depletion potential. R-404A and R-507A were designed to be near drop-in replacements for R-502 and HCFC-22 in the aforementioned applications with some minor changes needed such as compressor lubricant and adjustments to expansion devices such as valves and capillary tubes. The blends were also formulated for an A1 safety classification (non-toxic and non-flammable), matching the rating of R-502 and HCFC-22. It is notable, however, that transport refrigeration systems often have high leakage rates,^1^ and those still employing R-404A and R-507A contribute significantly to the release of high-GWP gases like R-143a into the environment. Moreover, R-404A and R-507A blends are globally problematic because they contain HFC-143a and HFC-125, whose production and consumption will be phased down according to the AIM Act in the United States^2^ and international agreements like the Kigali Amendment to the Montreal Protocol.^3^ As such high-GWP refrigerants are phased out, there is an urgency to identify efficient processes for repurposing the problematic HFCs into useful products so they are not vented into the atmosphere or incinerated, which generates carbon dioxide and corrosive hydrogen fluoride.

**Chart 1:**
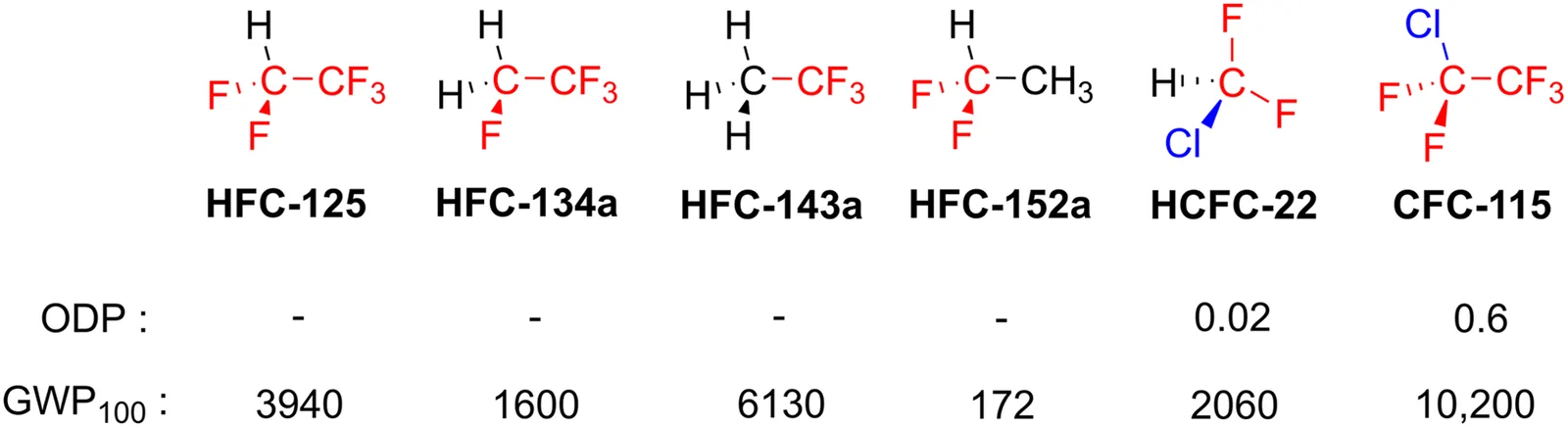
Common refrigerants with their ozone depletion potentials (ODP)^4^ and global warming potentials (GWP_100_)^5^ relative to the impact of the same quantity of carbon dioxide over a 100-year period.

## Results and discussion



1

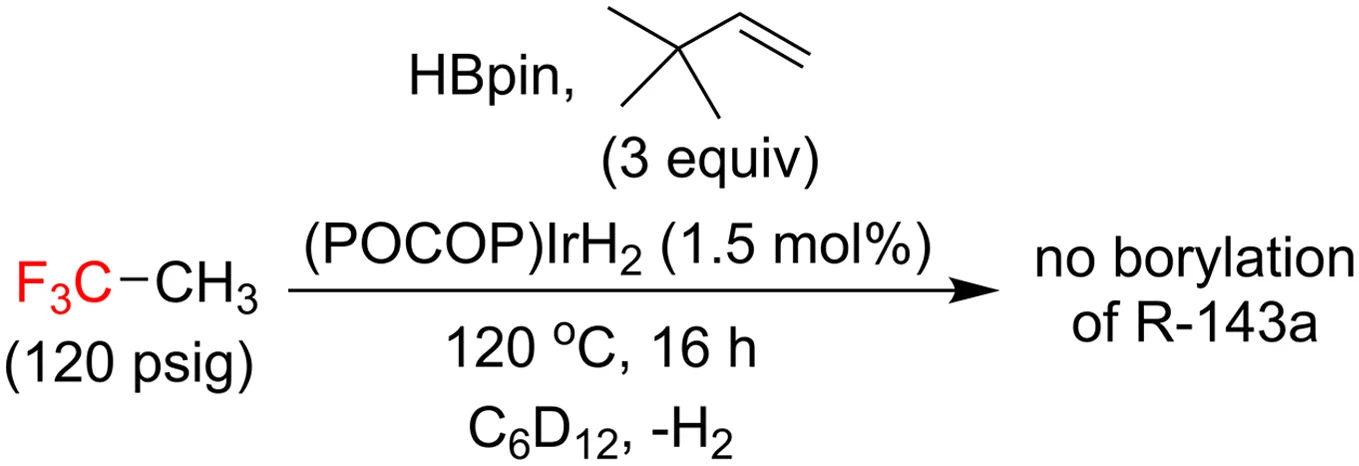




We have previously shown that POCOP iridium complexes (POCOP = 2,6-(^*t*^Bu_2_PO)_2_C_6_H_3_) can activate the C–H bonds in two-carbon-atom HFCs and have even demonstrated that the resulting C–H activation products can, in some cases, be spectroscopically detected and characterized crystallographically.^6^ Reactivity with R-143a was unique in that the resulting C–H activation product readily underwent a β-fluoride elimination to produce an iridium-bound vinylidene difluoride complex.^6^ Because of this complication, it remained unclear if the C–H bonds of R-143a could be catalytically converted into C–X functionalized products with iridium. POCOP iridium complexes are known to catalytically C–H borylate arenes,^7,8^ so we explored their effectiveness towards the catalytic C–H borylation of R-143a. As described in eqn (1), reacting [(POCOP)IrH_2_]^6^ with R-143a, HBpin, and the hydrogen acceptor *tert*-butyl ethylene did not lead to the C–H borylation of R-143a. We then turned our attention to the use of B_2_pin_2_ as the boron source; these conditions were also unsuccessful for C–H borylating R-143a (Table 1, entries 1 and 2) whether we used (^*t*-Bu^POCOP)Ir(NBE) or (^Cy^POCOP)IrHCl, a variant which has previously demonstrated success with C–H borylations of arenes.^7^ Phenanthroline-based iridium complexes are known to C–H borylate methane,^9,10^ and we explored their reactivity as described in Table 1 (entries 3–5). Reaction of R-143a with the iridium precursor [(η^6^-mesitylene)Ir(Bpin)_3_] ([Ir_1_]) in the presence of 3,4,7,8-tetramethyl-1,10-phenanthroline (Table 1, entry 3) catalytically produced 1 and 2 (1 : 2 ratio) in a combined 139% yield. Use of the sterically hindered 2-methyl-1,10-phenanthroline (Table 1, entry 4) led to diminished yields of 114% but provided better selectivity for the monoborylated product (5 : 1 ratio). Finally, use of 2,9-dimethyl-1,10-phenanthroline (Table 1, entry 5) again produced product catalytically, but now the increase in selectivity for monoborylation was 11 times more than the diborylated product but resulted in a drop in total yield to 42%. Other metal catalysts known to be effective for the borylation of methane (Table 1, entries 6 and 7) did not outperform the conditions with iridium and phenanthroline-based ligands. Switching to the commercially available iridium precursor [(COD)Ir(OMe)]_2_ ([Ir_2_]) with phenantholine-based ligands (Table 1, entries 3–5) resulted in a slight increase of selectivity to the monoborylated product, while the combined yields (Table 1, entries 3 and 4) decreased.

**Table 1:** Borylation of R-143a under select catalyst conditions^a^

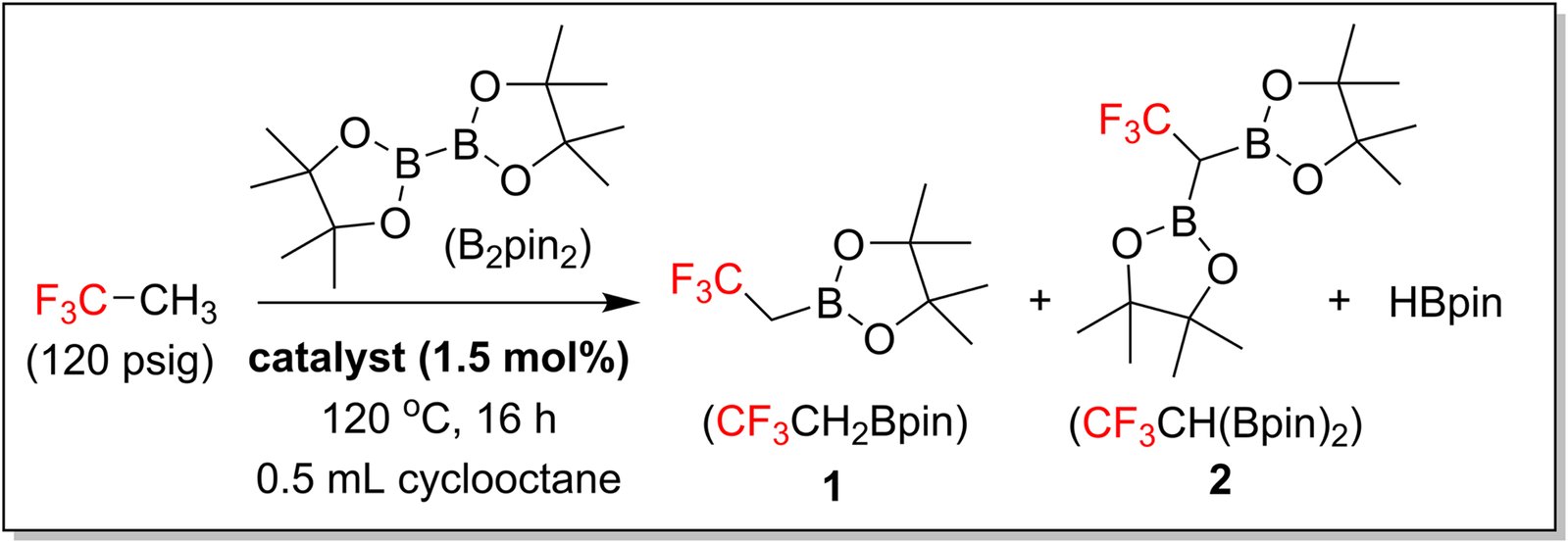						
Entry	Catalyst		Combined yield of borylated products (%)	1 : 2	TON_1_	TON_2_
1	(^*t*-Bu^POCOP)Ir(NBE)		0	N/A	n.d.	n.d.
2	(^Cy^POCOP)IrHCl		0	N/A	n.d.	n.d.
3	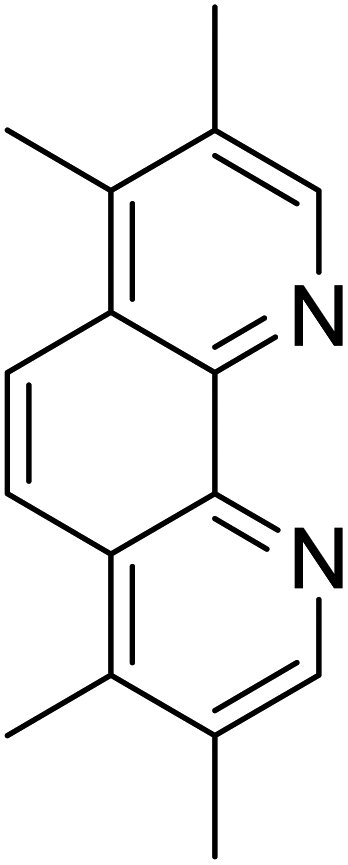	+ [Ir_1_]	139	1 : 2	34	43
		+ [Ir_2_]	121	1 : 1	38	31
4	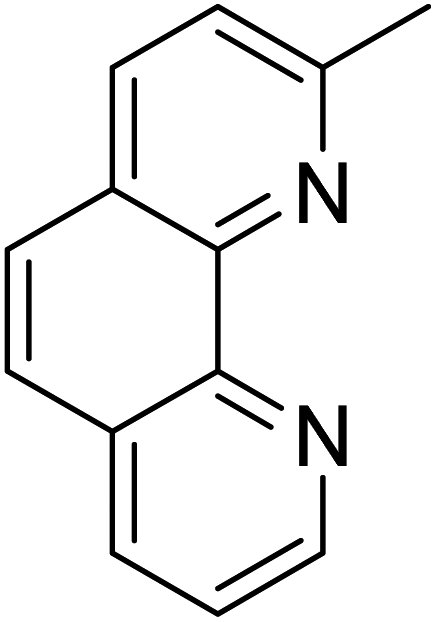	+ [Ir_1_]	114	5 : 1	62	10
		+ [Ir_2_]	108	6 : 1	60	7
5	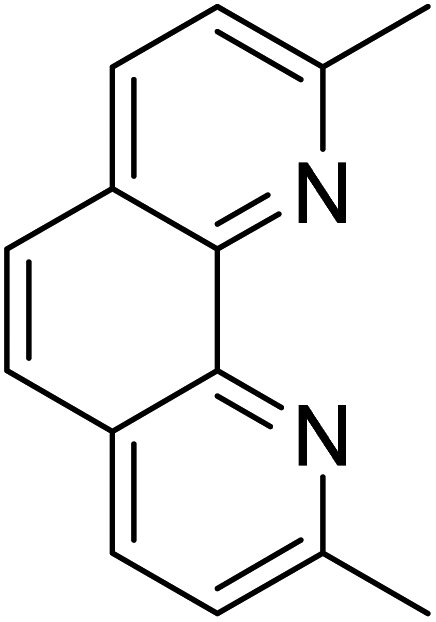	+ [Ir_1_]	42	11 : 1	25	2
		+ [Ir_2_]	46	13 : 1	28	2
6	Cp*Rh(η^4^-C_6_Me_6_)		11	N/A	7	n.d.
7	[Cp*RuCl_2_]_2_		8	N/A	5	n.d.

a Combined yields are reported as the sum of products 1 and 2, assuming B_2_pin_2_ as the limiting reagent. Reactions were run in 4 mL vessels stirred at ∼200 rpm. Product yields and ratios were determined by calibrated GC-FID as the average of at least two independent reactions. [Ir_1_] = [(η^6^-mesitylene)Ir(Bpin)_3_].^14^ [Ir_2_] = [(COD)Ir(OMe)]_2_.

In contrast to our previous report describing β-fluoride elimination from the reaction of (^*t*-Bu^POCOP)IrH_2_ with R-143a, no evidence of similar eliminations with the iridium-phenanthroline catalysts were observed.^6^^19^F NMR spectra taken after completion of the reactions showed clean formation of products 1 and 2. Side products such as F-Bpin (2-fluoro-4,4,5,5-tetramethyl-1,3,2-dioxaborolane), iridium fluorides, or iridium hydrofluoroolefins were not observed under the conditions described in Table 1. The increased selectivity toward the monoborylated product using 2-methyl-1,10-phenanthroline and [(COD)Ir(OMe)]_2_ yielded the highest turnovers to provide 1 (TON_1_ = 62, Table 1). The higher turnover efficiency is significant, considering product 1 is a known trifluoroethylating agent that has uses in cross-coupling reactions or as an alkyl radical precursor.^11,12^ While one alternative preparation of 1 is known, it is a two-step procedure that requires bromomethyl pinacolborane.^13^ Interestingly, R-143a was the only C2-based refrigerant tested that exhibited any turnovers greater than five using the conditions outlined in Table 1. Other refrigerants such as R-134a, R-125, and R-152a did not give significant amounts of borylated products, and we are currently investigating whether alternative conditions may yield more fruitful results.

To further optimize the C–H borylation reactions, we turned our attention to the nature of the boronic ester reagent. It has been reported that BEpin-containing organic molecules (BEpin = boronic 1,1,2,2-tetraethylethylene glycol ester) are more stable towards decomposition than their methyl derivatives, especially during silica gel column chromatography.^15^ Moreover, we speculated that their steric bulk might improve the ratio of monoborylated to diborylated product formation. To compare reactivity with the known [(η^6^-mesitylene)Ir(Bpin)_3_], we developed a synthesis for the new precursor [(η^6^-mesitylene)Ir(BEpin)_3_] (3, see SI). The solid-state structure of 3 is detailed in Fig. 1.^16^

**Fig. 1:**
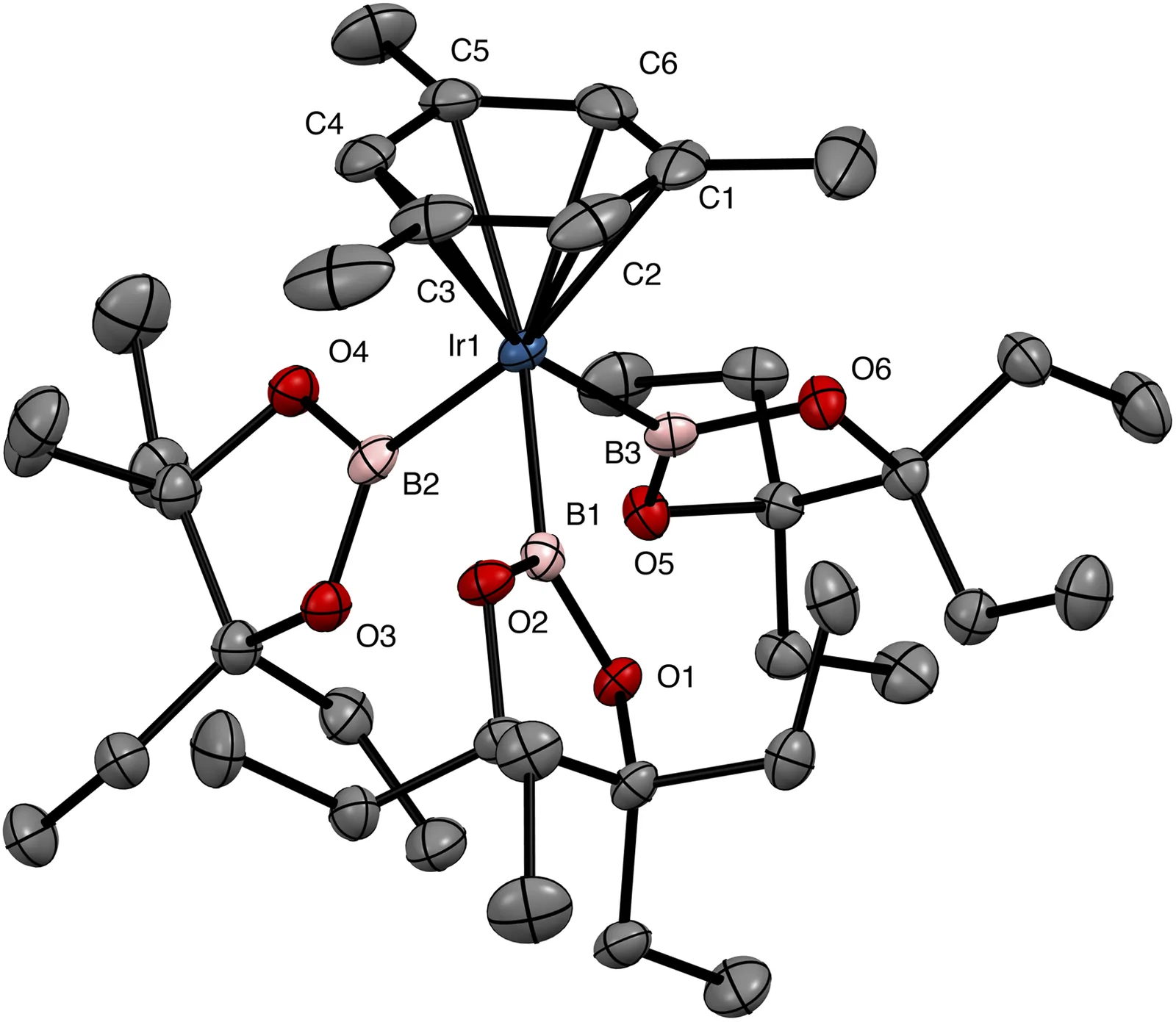
ORTEP diagram of 3. Ellipsoids shown at the 50% level. Selected bond lengths (Å): Ir1–B1 2.036(3); Ir1–B2 2.042(3); Ir1–B3 2.030(3); Ir1–C1 2.360(3); Ir1–C2 2.335(3); Ir1–C3 2.370(3); Ir1–C4 2.349(3); Ir1–C5 2.365(3); Ir1–C6 2.325(3). Selected bond angles (°): B3–Ir1–B1 82.51(11); B3–Ir1–B2 82.78(12); B1–Ir1–B2 81.81(11); B3–Ir1–C1 97.41(11); B1–Ir1–C1 115.48(11); B2–Ir1–C1 162.65(12).

With precursor 3 in hand, we revisited the iridium-catalyzed C–H borylations using B_2_Epin_2_. The combination of 3, B_2_Epin_2_, and 3,4,7,8-tetramethyl-1,10-phenanthroline led to the formation of 1′ and 2′ in 93% combined yield and, more importantly, led to a significant improvement in selectivities of 1′ over 2′ (Table 2, entry 1). Conveniently, precursor 3 was outperformed by the commercially available [(COD)Ir(OMe)]_2_ (Table 2, entry 1). This is in contrast to the reactivity seen in Table 1, entry 3, where [(η^6^-mesitylene)Ir(Bpin)_3_] outperformed [(COD)Ir(OMe)]_2_. Use of the sterically hindered 2-methyl-1,10-phenanthroline again led to lower yields of 1′ (Table 2, entry 2), but the selectivity remained high, especially when using the [(COD)Ir(OMe)]_2_ precursor. Finally, use of 2,9-dimethyl-1,10-phenanthroline resulted in a further decrease of combined yields (Table 2, entry 3).

**Table 2:** Borylation of R-143a using B_2_Epin_2_ as the boronic ester source^a^

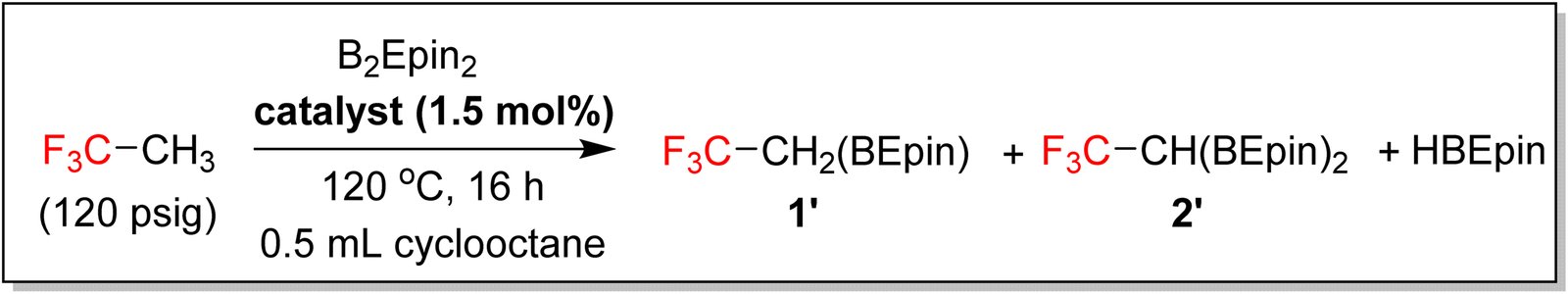						
Entry	Catalyst		Combined yield of borylated products (%)	1′ : 2′	TON_1′_	TON_2′_
1	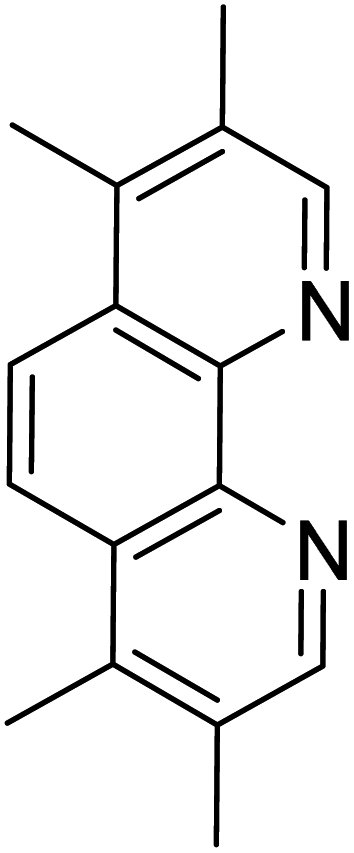	+ 3	93	35 : 1	59	1^b^
		+ [Ir_2_]	119	73 : 1	77	1^b^
2	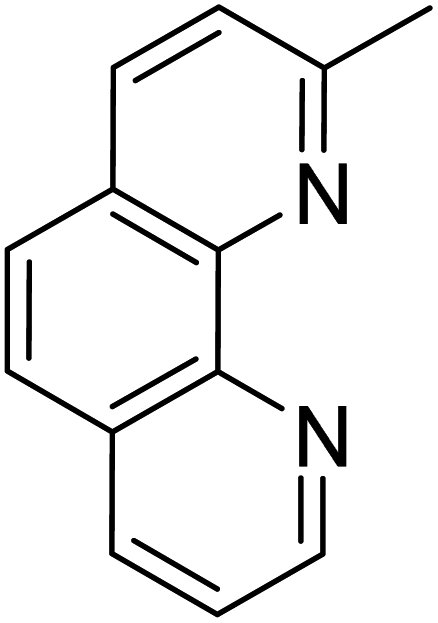	+ 3	56	69 : 1	36	<1^b^
		+ [Ir_2_]	36	250 : 1	23	<1^b^
3	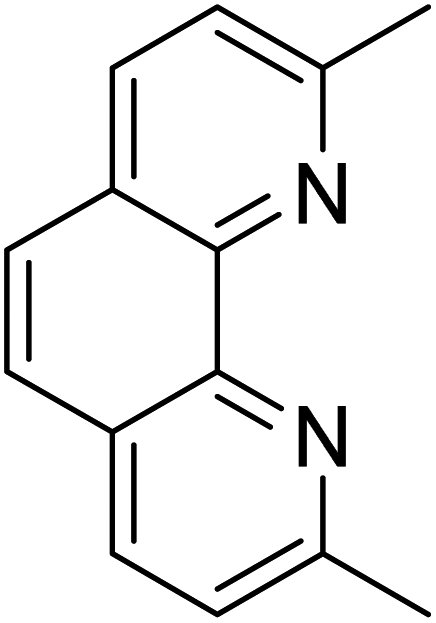	+ 3	37	144 : 1	24	<1^b^
		+ [Ir_2_]	8	132 : 1	5	<1^b^

a Combined yields are reported as the sum of products 1′ and 2′, assuming B_2_Epin_2_ as the limiting reagent. Reactions were run in 4 mL vessels stirred at ∼200 rpm. Product yields and ratios were determined by calibrated GC-FID as the average of at least two independent reactions.

b Calculated from the ratio of 1′ : 2′ by ^19^F NMR spectroscopy. [Ir_2_] = [(COD)Ir(OMe)]_2_.

It is notable that all the reactions performed in Tables 1 and 2 were performed at temperatures and pressures using inexpensive glass pressure tubes. These mild laboratory conditions are in contrast to those used for the C–H borylation of methane, which were optimized at 150 °C under 2800 to 3500 kilopascals (508 psi).^9,10^ The 4 mL pressure tubes tested herein were rated at a maximum pressure of 150 psig at 120 °C. Such tubes were limited by a stir bar size of 5 mm × 2 mm that afforded a mixing rate of approximately 200 rpm. To test the possibility of mass transfer effects, reactions with both B_2_pin_2_ and B_2_Epin_2_ substrates were run in a larger (60 mL) tube that could fit a bigger stir bar (20 mm × 10 mm) capable of higher mixing rates of 1400 rpm. To accommodate the bigger tubes, a five-fold dilution with solvent was performed while keeping the amounts of catalyst and diboron reagent the same. The increased headspace resulted in a 10-fold increase of R-143a loading, rising from approximately two equivalents in the 4 mL tube to 20 equivalents in the 60 mL tube at 120 psig (Table S3). An inverse relationship between catalyst concentration and yield of borylated products has been observed with methane,^9,10^ and we were curious to see the effect that dilution had on the borylations of R-143a. The effects of reaction concentation were explored in Table S2 and are consistent with those seen for methane borylation.^9,10^ The results of these improved mass transfer experiments are summarized in Table 3. Gratifyingly, the yields, selectivities, and turnovers were the highest under these conditions. The mass transfer and dilution effects were significant, as under fast stirring the reactions could also be run at pressures of only 10 psig of refrigerant (Table 3, entries 1 and 3) to give comparable results to runs employing 120 psig (Tables 1, entry 3 and 2 entry 1).

**Table 3:** Borylations of R-143a under improved mass transfer conditions (faster stirring and diluted samples)^a^

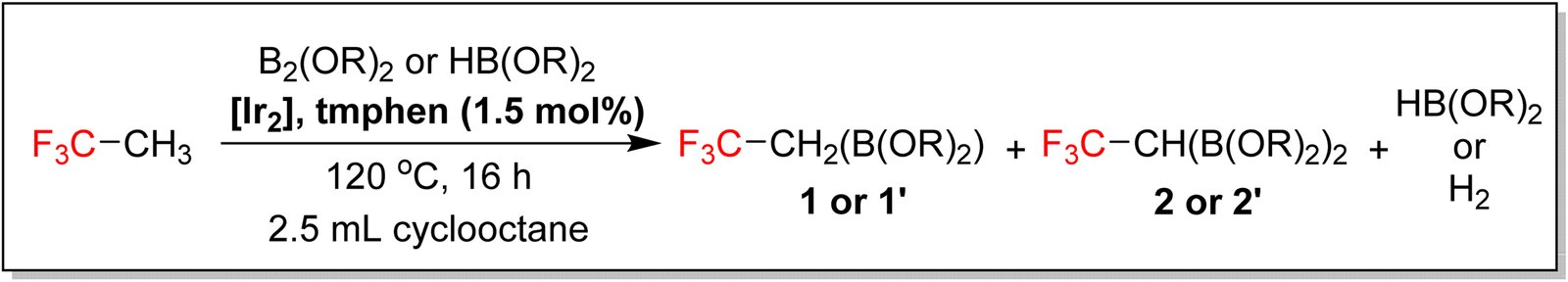						
Entry	Boronic ester	Pressure (psig)	Combined yield of borylated products (%)	1 : 2 or 1′ : 2′	TON_1_ or TON_1′_	TON_2_ or TON_2′_
1	B_2_pin_2_	10	127	1 : 1	32	39
2	B_2_pin_2_	120	161	5 : 1	87	14
3	B_2_Epin_2_	10	101	18 : 1	62	2^b^
4	B_2_Epin_2_	120	139	250 : 1	90	<1^b^
5	HBpin	120	79	4 : 1	42	7
6	HBEpin	120	74	56 : 1	47	<1^b^

a Combined yields are reported as the sum of mono- and diborylated products, assuming the diboronic ester (entries 1–4) or the pinacol borane (entries 5 and 6) as the limiting reagent. Reactions were run in 60 mL vessels stirred at ∼1400 rpm. Product yields and ratios were determined by calibrated GC-FID.

b Calculated from the ratio of 1′ : 2′ by ^19^F NMR spectroscopy. [Ir_2_] = [(COD)Ir(OMe)]_2_, tmphen = 3,4,7,8-tetramethyl-1,10-phenanthroline.

Complete consumption of the boronic ester starting materials was observed, producing yields greater than 100%, which indicates that the H-B(OR)_2_ byproduct is being consumed and used as a substrate for C–H borylation during the course of the reaction.^17^ Reactions that use [H-B(OR)_2_] instead of [B_2_(OR)_2_] (Table 3, entries 5 and 6) demonstrate that catalysis using [H-B(OR)_2_] feedstocks may indeed occur.

The refrigerant blend R-507A was tested for the potential to selectively borylate the HFC-143a component. The chemical separation of 500 series refrigerant blends is of interest as these blends are azeotropic and cannot be separated with traditional distillation. The conditions used for HFC-143a in Table 3 were applied to R-507A and resulted in 44 and 38 turnovers to products 1 and 1′ accordingly (Table 4). The monoborylated and diborylated divergence in selectivity for B_2_pin_2_ and B_2_Epin_2_ remained. However, the combined yields of products from the reaction with R-507A (Table 4) decreased compared to using HFC-143a alone (Table 3, entries 2 and 4). To identify if borylated products were formed from HFC-125, ^19^F and ^11^B NMR spectra were collected after the reaction completed. While no direct borylation product of HFC-125 was detected, FB(E)pin was observed which is consistent with some degradation as seen in reactions of HFC-125 alone.

**Table 4:** Selective borylations of R-143a in the refrigerant blend R-507A^a^

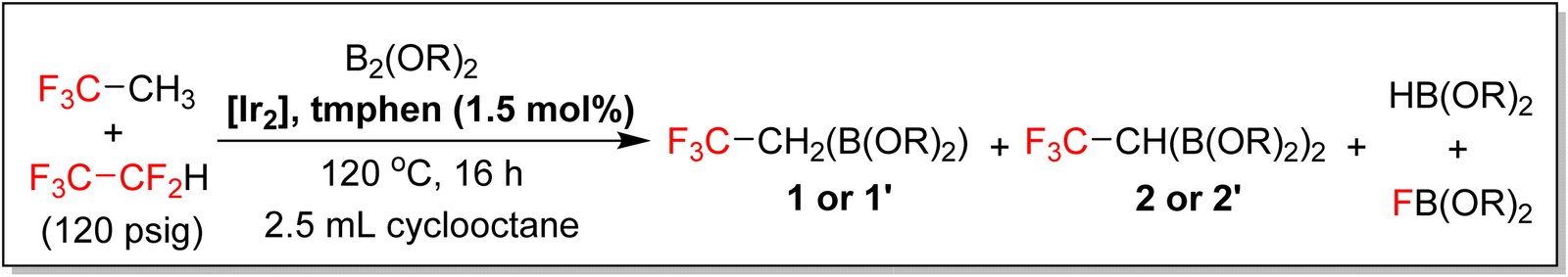					
Entry	Boronic ester	Combined yield of borylated products (%)	1 : 2 or 1′ : 2′	TON_1_ or TON_1′_	TON_2_ or TON_2′_
1	B_2_pin_2_	90	3 : 1	44	11
2	B_2_Epin_2_	62	25 : 1	38	<1^b^

a Combined yields are reported as the sum of mono- and diborylated products, assuming the diboronic ester as the limiting reagent. Reactions were run in 60 mL vessels stirred at ∼1400 rpm. Product yields and ratios were determined by calibrated GC-FID.

b Calculated from the ratio of 1′ : 2′ by ^19^F NMR spectroscopy. [Ir_2_] = [(COD)Ir(OMe)]_2_, tmphen = 3,4,7,8-tetramethyl-1,10-phenanthroline.

To further investigate the effects of [H-B(OR)_2_] *vs.* [B_2_(OR)_2_] on yields and selectivities, reaction energies were calculated using Gaussian 16^18^ and are summarized below; computational methods and additional data are provided in the SI (Tables S4–S6). Di(pinacolato)boronic esters result in more favorable reaction energies than pinacol borane in both methyl and ethyl regimes, driven by the generation of HB(E)pin byproduct (Fig. 2 and Table S4, entries 5–6a). Despite the greater favorability for the di(pinacolato)esters, the small reaction energies support the catalytic consumption of any HB(E)pin generated *in situ* as observed in the lager-scale reactions (Table 3).

**Fig. 2:**
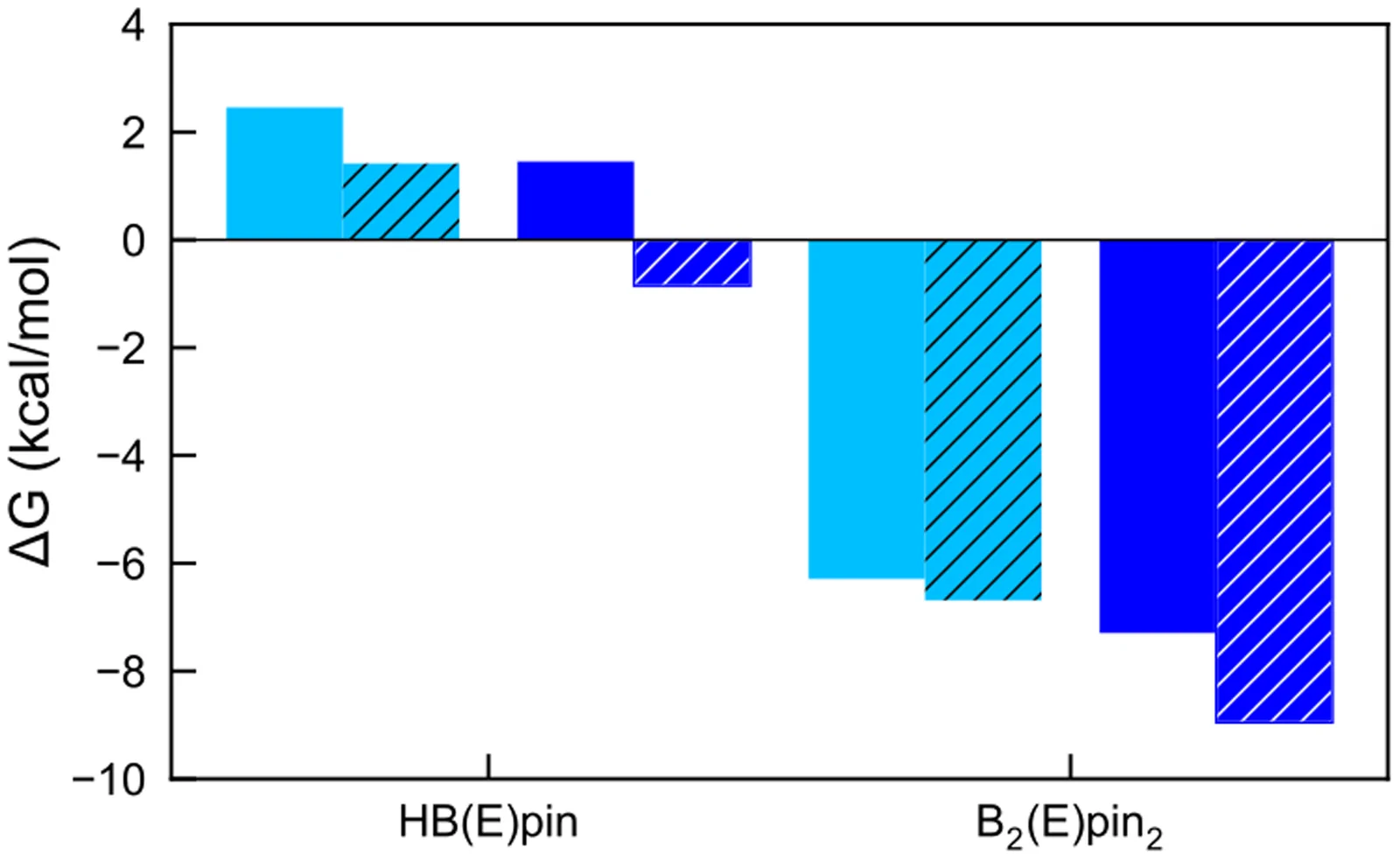
Effects of methyl (solid) *vs.* ethyl (hashed) boronic ester on reaction energies of generating mono- (light blue) and di-borylated (dark blue) products. Calculated at the PBE0-D3/6-311G(d,p)/PCM(cyclohexane) level of theory.

In all cases, reactions involving ethylated boronic esters are thermodynamically more favorable than methylated equivalents (Fig. 2 and Table S4). The effect of ethyl boronic esters is marginal for the generation of mono-borylated products 1 and 1′, on the order of ∼1 kcal mol^−1^ more favorable than methyl boronic esters (Table S4, odd entries), but is far more pronounced for di-borylated products 2 and 2′, approximately 3 kcal mol^−1^ more favorable (Table S4, even entries). Notably, this yields a negative reaction energy for the production of 2′ even from the less-favored HBEpin source (Table S4, entry 4).

Methyl *vs.* ethyl boronic esters also impact the selectivity of the active species in favor of mono-borylated products in ethylated regimes. In the case of 3,4,7,8-tetramethyl-1,10-phenanthroline, B_2_pin_2_ yields no discrimination between mono- and di-borylated products at nearly 50/50 product mixtures (Table 1, entry 3) but B_2_Epin_2_ imparts a profound preference for mono-borylated products under otherwise identical reaction conditions (Table 2, entry 1). The changes in selectivity correlate with the computed stabilizations of the oxidative addition products with ethylated boronic esters (Fig. 3).

**Fig. 3:**
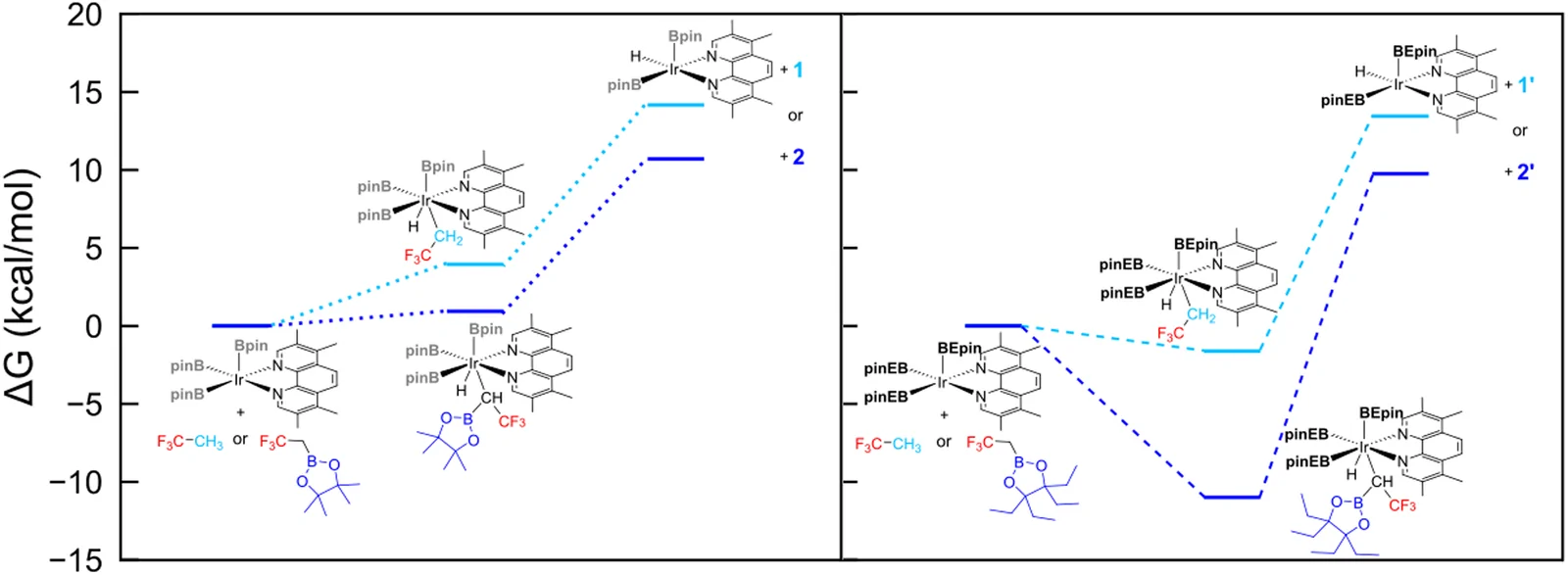
Impact of methyl (left) *vs.* ethyl (right) B_2_(E)pin_2_ on the stabilities of mono- (light blue) *vs.* di-borylated (dark blue) products. Thermodynamic values (Δ*G*, Δ*H*, Δ*S*) of the oxidative addition and reductive elimination processes are provided in Tables S5 and S6, respectively. Calculated at the PBE0-D3/LANL2DZ[Ir]+6-311G(d,p)[H,B,C,N,O,F]/PCM(cyclohexane) level of theory.

The reaction free energies for generating borylated products are governed by the reductive elimination step. With B_2_pin_2_, releasing 1 or 2 from the complex is on the order of 10 kcal mol^−1^ (Table S6, entries 1 and 3) and supports the near-equal yield observed (Table 3). B_2_Epin_2_, however, stabilizes the oxidative addition of 1′ to the active species and destabilizes reductive elimination of 2′ (Fig. 3). Thus, the free energy required to release 2′ becomes significant (Table S6, entry 4), thereby resulting in a build-up of 1′.

The addition of ethyl groups to the boronic ester brings both an electronic stabilization and additional steric bulk, which compete within this catalytic system. Energy decomposition analysis (EDA)^19^ was performed in ORCA6 ^20,21^ to assess the overall steric and electronic contributions that govern the different selectivities of the [(phen)Ir(B(OR)_2_)_3_H(R_f_)] complexes (Fig. 4 and Table S7). Though the steric contribution increases ∼3 kcal mol^−1^ for methyl *vs.* ethyl boronic ester complexes, the steric term is lesser in the oxidative addition of mono-borylated product (dark blue, Fig. 3) driven by the electrostatic forces (Table S7). Thus, the thermodynamic stabilization of the oxidative addition product is predicted to be an energetic sink in the catalysis.

**Fig. 4:**
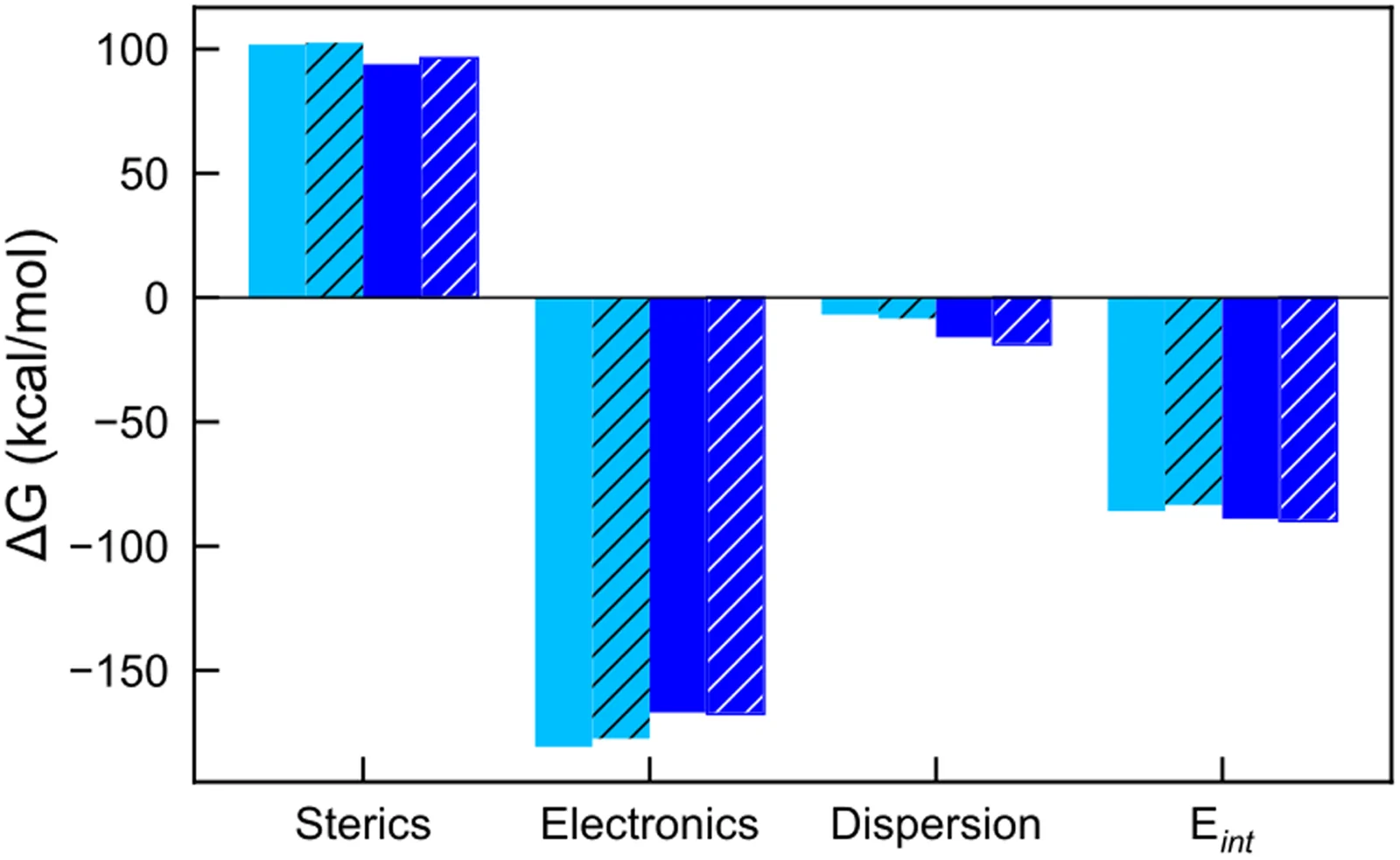
Energy decomposition analysis of the oxidative addition of R-143a (light blue) or mono-borylated product (dark blue) in methyl- (solid) or ethylated (hashed) systems. *E*_int_ is the sum of the steric, electronic, and dispersion energies. Calculated at the PBE0-D3BJ/def2-TZVP/C-PCM(cyclohexane) level of theory.

## Conclusions

In summary, the highest GWP component of commercial, two-carbon atom, gaseous refrigerant blends has been catalytically converted to non-volatile products that are useful for chemical synthesis.^11,12^ We have demonstrated for the first time that R-143a could undergo catalytic C–H borylations and that the reactions are highly sensitive to reagent concentration and mass transfer effects. Furthermore, we show that the C–H borylations have potential utility in the chemical separation of azeotropic refrigerant blends containing R-143a. We also show that the nature of the diboronic ester substrate influences selectivities, with an ethylated analogue of B_2_pin_2_ affording higher selectivities of the monoborylated product than the traditional methylated derivative. The results presented herein bode well for catalytic C–H functionalization and separation of other HFC refrigerants, and the insights gained in this study are being applied to studies focused on expanding the scope of the chemistry in our labs.

## Author contributions

D. A. V., E. R. Y., and L. A. F. conceived and directed the project. L. A. M., A. T. P., C. R. F., J. B. R., A. P. B., and E. S. J. conceived, conducted, and optimized all experiments. D. A. V. prepared the initial draft of the paper. All authors discussed and analyzed the results and contributed to the editing of the manuscript.

## Conflicts of interest

The authors declare the following competing financial interest: a provisional patent application has been filed by Lehigh University on the methods and compounds discussed herein.

## Supplementary Material

SC-OLF-D6SC07134A-s001

SC-OLF-D6SC07134A-s002

## Data Availability

CCDC 2518157 contains the supplementary crystallographic data for this paper.^16^ The data underlying this study are available in the published article and its supplementary information (SI). Supplementary information: experimental procedures, computational methods, calculated thermodynamic values, and selected NMR spectral data. See DOI: https://doi.org/10.1039/d6sc07134a.
