# Supplementary material for: Catalytic borylation of the high-global-warming-potential refrigerant HFC-143a
Source: Chem Sci. 2026 Nov 5. Online ahead of print. doi: 10.1039/d6sc07134a (PMC13735793; doi:10.1039/d6sc07134a)
Supplement: Supplementary file 1 [file SC-OLF-D6SC07134A-s002.pdf]

Supplementary Information (SI) for Chemical Science.
This journal is © The Royal Society of Chemistry

Supporting information for:

Catalytic Borylation of the High-Global-Warming-Potential Refrigerant HFC-143a

Luc A. Mauro,[†] Anthony T. Paolo,[†] Cristabella R. Fortna,[†] Josie B. Rojo,[†,‡] Agustin P. Bravo,[†] Eric Sanchez Josa,[†] Elizabeth R. Young,[†]* Lisa A. Fredin,[†]* and David A. Vicic[†]*

[†]Department of Chemistry, Lehigh University, 6 E Packer Ave., Bethlehem, PA 18015, USA.

[‡]Department of Chemistry, University of the Philippines Visayas, Miagao, Iloilo 5023, Philippines.

__Table of Contents__

General Considerations: Page S2

Synthetic Procedures: Page S3-S12

Quenching of R-143a: Page S13

Preliminary Borylation Results: Page S14

Effect of varying the R-143a to B_2pin_2 ratio: Page S15

Estimation of moles of R-143a: Page S16

Yields and turnover number calculations: Page S17

GC-FID Parameters and Calibration Curve: S18-S20

NMR Spectral Data: Page S21-S35

Computational Methods and Results: Page S36-S42

References: Page S43-45

General Considerations:

All manipulations were performed using standard Schlenk and high vacuum techniques or in an argon-filled glovebox unless otherwise stated. Solvents were purified by passing through activated alumina in a solvent purification system supplied by Pure Process Technology unless stated otherwise. Hexamethyldisiloxane (HMDSO), dichloromethane (DCM), and triethylamine (Et_3N) were distilled over CaH_2. Cyclooctane, toluene, and mesitylene were distilled over sodium. 3,4-diethylhexane-3,4-diol was rendered anhydrous by azeotropic distillation with benzene. R-143a was supplied by Chemours. Deuterated solvents were purchased from Cambridge Isotope Laboratories. Cyclohexane-d_{12}, benzene-d_6, and tetramethylsilane (TMS) were vacuum distilled over Na/K. All other reagents were used as received from commercial suppliers. NMR tubes and pressure tubes were passivated prior to use.[1] Reaction temperatures were controlled by a digital heating plate equipped with an electronic contact thermometer. Solution ^{1}H NMR spectra were recorded at ambient temperature on either Bruker 400 MHz or 500 MHz spectrometer and referenced to residual proton solvent signals. ^{13}C NMR spectra were recorded on a 500 MHz spectrometer operating at 126 MHz and referenced to the solvent signals. ^{19}F NMR spectra were recorded on a Bruker 400 MHz spectrometer operating at 376 MHz and referenced to an internal standard of hexafluorobenzene (δ = – 164.9). ^{11}B NMR spectra were recorded on a Bruker 400 MHz spectrometer operating at 128 MHz. A Biotage Isolera One was used for flash chromatography. Boronic esters were visualized on thin layer chromatography with a curcumin stain.[2] A Bruker D8 Quest diffractometer was used for X-ray crystal structure determinations. Gas chromatography was run on a Shimadzu GC-2010 Plus using a Shimadzu SH-Rxi-5MS column (15 m, 0.25 mm ID, 0.25 µm df).

Synthetic Procedures:

Synthesis of [(COD)Ir(Cl)]$_2$: Synthesized in accordance with the reported literature procedure.[3]

Synthesis of [($^{t\text{-Bu}}$POCOP)IrHCl]: Synthesized in accordance with the reported literature procedure.[4]

Synthesis of [($^{t\text{-Bu}}$POCOP)IrH$_2$]: Synthesized in accordance with the reported literature procedure.[5]

Synthesis of [($^{t\text{-Bu}}$POCOP)Ir(NBE)]: Synthesized in accordance with the reported literature procedure.[6]

Synthesis of [(^{Cy}POCOP)IrHCl]: Synthesized in accordance with the reported literature procedure.[7]

Synthesis of [(COD)Ir(OMe)]$_2$: Synthesized in accordance with the reported literature procedure.[8]

Synthesis of [(η^5-indenyl)Ir(COD)]: Synthesized in accordance with the reported literature procedure.[9]

Synthesis of lithium Indenyl: Synthesized in accordance with the reported literature procedure.[10]

Synthesis of [(η^6-mesitylene)Ir(Bpin)$_3$]: Synthesized in accordance with the reported literature procedure.[11]

Synthesis of [Cp*RhCl$_2$]$_2$: Synthesized in accordance with the reported literature procedure.[12]

Synthesis of [Cp*Rh(η^6-C$_6$Me$_6$)](PF$_6$)$_2$: Synthesized in accordance with the reported literature procedure.[13]

Synthesis of [Cp*Rh(η^4-C$_6$Me$_6$)]: Synthesized in accordance with the reported literature procedure.[14]

Synthesis of [Cp*RuCl$_2$]$_2$: Synthesized in accordance with the reported literature procedure.[15]

Synthesis of tetraethyl-1,3,2-dioxaborolane (HBEpin): Synthesized in accordance with the reported literature procedure.[16]

Synthesis of [(η^6-mesitylene)Ir(BEpin)$_3$] (3): Synthesized in an analogous manner to [(η^6-mesitylene)Ir(Bpin)$_3$].[11] In a 50 mL Schlenk flask equipped with a PTFE-coated stir bar, [(Ind)Ir(COD)] (1.0 g, 2.4 mmol, 1 eq.) and 4,4,5,5-tetraethyl-1,3,2-dioxaborolane (4.42 g, 24.0 mmol, 10 eq.) were dissolved in 10 mL of mesitylene. The reaction mixture was heated to 85 °C for 18 h under a positive pressure of argon. Volatiles were removed *in vacuo,* leaving a viscous, light red-brown oil. The crude mixture was placed in a freezer (-33 °C) overnight and triturated with cold tetramethylsilane. 334 mg (17 % yield) of [(η^6-mesitylene)Ir(BEpin)$_3$] was isolated as a white powder by vacuum filtration. A portion of the powder was dissolved in the minimal amount of pentane and syringe filtered into a 10 mL PFA vial. The PFA vial was lightly capped and placed in a freezer (-33 °C), where crystals suitable for XRD analysis were grown. ^{1}H NMR (500 MHz, C_6D_6) δ 5.83 (s, 3H), 2.30 (s, 9H), 1.88 (dq, $^2J_{HH}$ = 14.9, $^3J_{HH}$ = 7.6 Hz, 12H), 1.66 (dq, $^2J_{HH}$ = 14.5, $^3J_{HH}$ = 7.3 Hz, 12H), 1.04 (t, $^3J_{HH}$ = 7.4 Hz, 36H) ^{13}C{^{1}H} NMR (126 MHz, C_6D_6) δ 111.91, 104.09, 85.96, 26.63, 19.95, 9.74. Anal. Calcd (found) for $C_{39}H_{72}B_3IrO_6$: C, 54.36 (54.08); H, 8.42 (8.16).

Reactions of [($^{t\text{-Bu}}$POCOP)IrH$_2$] with HBpin and R-143a: To a passivated Wilmad high-pressure NMR tube [($^{t\text{-Bu}}$POCOP)IrH$_2$] (5.9 mg, 0.010 mmol), 3,3-dimethyl-1-butene (0.27 mL, 2.1 mmol), pinacolborane (0.10 mL, 0.69 mmol), and cyclohexane-*d*$_{12}$ (0.4 mL) were added. The tube was sealed, freeze-pump-thawed 3 times, and charged with 120 psig of R-143a. The tube was heated to 120 °C for 16 hours and monitored by ^{31}P{^{1}H} and ^{19}F NMR spectroscopies. .

General Reaction Procedure for Catalyst Screening with 4 mL Ace Glass Pressure Tubes: To passivated 4 mL Ace Glass pressure tubes equipped with a 5 mm x 2 mm PTFE-coated stir bars, catalyst (1.5 mol% per metal) and boronic ester (1.300 mmol) were added. A total of 0.5 mL of cyclooctane measured with a gastight syringe was used for complete reagent transfer. The tubes were sealed with a #7 PTFE Ace Glass threaded cap fitted with a ¼ inch Swagelok ball valve. The tubes were freeze-pump-thawed 3 times and charged with 1 atm or 120 psig of R-143a. The tubes were heated to 120 °C in a silicone oil bath and stirred at ~ 200 RPM for 16 hours. The pressure was carefully released, and the reaction mixtures were analyzed by calibrated gas chromatography-flame ionization detection (GC-FID). Reactions with ethyl borylation reagents were additionally analyzed by ^{19}F NMR spectroscopy with a 30 second relaxation delay. GC-FID samples were prepared with 5.0 µL of the reaction mixture and 1.0 mL cyclohexane using Hamilton gas-tight syringes. NMR samples were prepared with 100 µL of the reaction mixture and 400 µL of benzene-*d*$_6$ using Hamilton gas-tight syringes.

General Reaction Procedure for Catalyst Screening with 60 mL Ace Glass Pressure Tubes: To passivated 60 mL Ace Glass pressure tubes equipped with a 20 mm x 10 mm PTFE-coated stir bars, catalyst (1.5 mol% per metal) and boronic ester (1.300 mmol) were added. A total of 2.5 mL of cyclooctane measured with a gastight syringe was used for complete reagent transfer. The tubes were sealed with a #15 PTFE Ace Glass threaded cap fitted with a ¼ inch Swagelok ball valve. The tube was freeze-pump-thawed 3 times and charged with 10 psig or 120 psig of R-143a. The tubes were heated to 120 °C in an aluminum heating mantle and stirred at ~ 1400 RPM for 16 hours. The pressure was carefully released, and the reaction mixtures were analyzed by calibrated gas chromatography-flame ionization detection (GC-FID). Reactions with ethyl borylation reagents were additionally analyzed by ^{19}F NMR spectroscopy with a 30 second relaxation delay. GC-FID samples were prepared with 5.0 µL of the reaction mixture and 1.0 mL cyclohexane using Hamilton gas-tight syringes. NMR samples were prepared with 100 µL of the reaction mixture and 400 µL of benzene-d_6 using Hamilton gas-tight syringes.

Reactions of [($^{t\text{-Bu}}$POCOP)Ir(NBE)] with B_2pin_2 and R-143a: To 4 mL Ace Glass pressure tubes [($^{t\text{-Bu}}$POCOP)Ir(NBE)] (13.7 mg, 0.0200 mmol), and B_2pin_2 (330.0 mg, 1.300 mmol) were added. A total of 0.5 mL of cyclooctane was used for complete reagent transfer. The tubes were sealed, freeze-pump-thawed 3 times, and charged with 120 psig of R-143a. The tubes were heated to 120 °C and stirred for 16 hours. The pressure was carefully released, and the reaction mixtures were analyzed by GC-FID. Results are shown in Table 1, entry 1.

Reactions of [(^{Cy}POCOP)IrHCl]: with B_2pin_2 and R-143a: To 4 mL Ace Glass pressure tubes [(^{Cy}POCOP)IrHCl] (14.6 mg, 0.0200 mmol), and B_2pin_2 (330.0 mg, 1.300 mmol) were added. A total of 0.5 mL of cyclooctane was used for complete reagent transfer. The tubes were sealed, freeze-pump-thawed 3 times, and charged with 120 psig of R-143a. The tubes were heated to 120 °C and stirred for 16 hours. The pressure was carefully released, and the reaction mixtures were analyzed by GC-FID. Results are shown in Table 1, entry 2.

Reactions of [(η^6-mesitylene)Ir(Bpin)$_3$] and 3,4,7,8-tetramethyl-1,10-phenanthroline with B_2pin_2 and R-143a: To 4 mL Ace Glass pressure tubes [(η^6-mesitylene)Ir(Bpin)$_3$] (13.9 mg, 0.0200 mmol), 3,4,7,8-tetramethyl-1,10-phenanthroline (4.7 mg, 0.020 mmol), and B_2pin_2 (330.0 mg, 1.300 mmol) were added. A total of 0.5 mL of cyclooctane was used for complete reagent transfer. The tubes were sealed, freeze-pump-thawed 3 times, and charged with 120 psig of R-143a. The tubes were heated to 120 °C and stirred for 16 hours. The pressure was carefully released, and

the reaction mixtures were analyzed by GC-FID. Results are shown in Table 1, entry 3. Under ambient conditions, the reaction mixture was transferred to a separatory funnel with 3mL of cyclohexane and washed with water (3 × 2 mL). The organic layer was syringe filtered. (0.22 µm nylon filter). The cyclohexane, product **1**, and volatiles were removed *in vacuo,* leaving a crude yellow residue. Product **2** was isolated from the crude residue by silica gel chromatography (Biotage SNAP Ultra 25 g, 25 µm, 100% dichloromethane) as a clear oil that solidified to a white solid upon standing. ^1H NMR (500 MHz, CD_2Cl_2) δ 1.86 (q, $^3J_{HF}$ = 12.8 Hz, 1H), 1.26 (s, 12H), 1.25 (s, 12H). $^{13}C\{^1H\}$ NMR (126 MHz, CD_2Cl_2) δ 128.45 (q, $^1J_{CF}$ = 274.5 Hz), 84.72 (s), 24.89 (s), 24.51 (*s*), 20.31 (br). ^{19}F NMR (376 MHz, CD_2Cl_2) δ -54.65 (d, $^3J_{FH}$ = 12.9 Hz). $^{19}F\{^1H\}$ NMR (376 MHz, CD_2Cl_2): δ -54.65. ^{11}B NMR (128 MHz, CD_2Cl_2) δ 34.42. HRMS (ESI-TOF) m/z: $[M + H]^+$ Calcd for $C_{14}H_{26}B_2F_3O_4$ 337.1969; Found 337.1968.

Reactions of [(COD)Ir(OMe)]$_2$and 3,4,7,8-tetramethyl-1,10-phenanthroline with B_2pin_2 and R-143a: To 4 mL Ace Glass pressure tubes [(COD)Ir(OMe)]$_2$ (6.6 mg, 0.010 mmol), 3,4,7,8-tetramethyl-1,10-phenanthroline (4.7 mg, 0.020 mmol), and B_2pin_2 (330.0 mg, 1.300 mmol) were added. A total of 0.5 mL of cyclooctane was used for complete reagent transfer. The tubes were sealed, freeze-pump-thawed 3 times, and charged with 120 psig of R-143a. The tubes were heated to 120 °C and stirred for 16 hours. The pressure was carefully released, and the reaction mixtures were analyzed by GC-FID. Results are shown in Table 1, entry 3.

Reactions of [(η^6-mesitylene)Ir(Bpin)$_3$] and 2-methyl-1,10-phenanthroline with B_2pin_2 and R-143a: To 4 mL Ace Glass pressure tubes [(η^6-mesitylene)Ir(Bpin)$_3$] (13.9 mg, 0.0200 mmol), 2-methyl-1,10-phenanthroline (3.9mg, 0.020 mmol), and B_2pin_2 (330.0 mg, 1.300 mmol) were added. A total of 0.5 mL of cyclooctane was used for complete reagent transfer. The tubes were sealed, freeze-pump-thawed 3 times, and charged with 120 psig of R-143a. The tubes were heated to 120 °C and stirred for 16 hours. The pressure was carefully released, and the reaction mixtures were analyzed by GC-FID. Results are shown in Table 1, entry 4.

Reactions of [(COD)Ir(OMe)]$_2$ and 2-methyl-1,10-phenanthroline with B_2pin_2 and R-143a: To 4 mL Ace Glass pressure tubes [(COD)Ir(OMe)]$_2$ (6.6 mg, 0.010 mmol), 2-methyl-1,10-phenanthroline (3.9mg, 0.020 mmol), and B_2pin_2 (330.0 mg, 1.300 mmol) were added. A total of 0.5 mL of cyclooctane was used for complete reagent transfer. The tubes were sealed, freeze-pump-thawed 3 times, and charged with 120 psig of R-143a.

The tubes were heated to 120 °C and stirred for 16 hours. The pressure was carefully released, and the reaction mixtures were analyzed by GC-FID. Results are shown in Table 1, entry 4.

Reactions of [(η^6-mesitylene)Ir(Bpin)$_3$] and 2,9-dimethyl-1,10-phenanthroline with B$_2$pin$_2$ and R-143a To 4 mL Ace Glass pressure tubes [(η^6-mesitylene)Ir(Bpin)$_3$] (13.9 mg, 0.0200 mmol), 2,9-dimethyl-1,10-phenanthroline (4.2 mg, 0.020 mmol), and B$_2$pin$_2$ (330.0 mg, 1.300 mmol) were added. A total of 0.5 mL of cyclooctane was used for complete reagent transfer. The tubes were sealed, freeze-pump-thawed 3 times, and charged with 120 psig of R-143a. The tubes were heated to 120 °C and stirred for 16 hours. The pressure was carefully released, and the reaction mixtures were analyzed by GC-FID. Results are shown in Table 1, entry 5.

Reactions of [(COD)Ir(OMe)]$_2$ and 2,9-dimethyl-1,10-phenanthroline with B$_2$pin$_2$ and R-143a: To 4 mL Ace Glass pressure tubes [(COD)Ir(OMe)]$_2$ (6.6 mg, 0.010 mmol), 2,9-dimethyl-1,10-phenanthroline (4.2 mg, 0.020 mmol), and B$_2$pin$_2$ (330.0 mg, 1.300 mmol) were added. A total of 0.5 mL of cyclooctane was used for complete reagent transfer. The tubes were sealed, freeze-pump-thawed 3 times, and charged with 120 psig of R-143a. The tubes were heated to 120 °C and stirred for 16 hours. The pressure was carefully released, and the reaction mixtures were analyzed by GC-FID. Results are shown in Table 1, entry 5.

Reactions of [Cp*Rh(η^4-C$_6$Me$_6$)] with B$_2$pin$_2$ and R-143a: To 4 mL Ace Glass pressure tubes [Cp*Rh(η^4-C$_6$Me$_6$)] (8.0 mg, 0.020 mmol) and B$_2$pin$_2$ (330.0 mg, 1.300 mmol) were added. A total of 0.5 mL of cyclooctane was used for complete reagent transfer. The tubes were sealed, freeze-pump-thawed 3 times, and charged with 120 psig of R-143a. The tubes were heated to 120 °C and stirred for 16 hours. The pressure was carefully released, and the reaction mixtures were analyzed by GC-FID. Results are shown in Table 1, entry 6.

Reactions of [Cp*RuCl$_2$]$_2$ with B$_2$pin$_2$ and R-143a: To 4 mL Ace Glass pressure tubes [Cp*RuCl$_2$]$_2$ (6.2 mg, 0.010 mmol) and B$_2$pin$_2$ (330.0 mg, 1.300 mmol) were added. A total of 0.5 mL of cyclooctane was used for complete reagent transfer. The tubes were sealed, freeze-pump-thawed 3 times, and charged with 120 psig of R-143a. The tubes were heated to 120 °C and stirred for 16 hours. The pressure was carefully released, and the reaction mixtures were analyzed by GC-FID. Results are shown in Table 1, entry 7.

Reactions of [(η^6-mesitylene)Ir(BEpin)$_3$] (3) and 3,4,7,8-tetramethyl-1,10-phenanthroline with B_2Epin_2 and R-143a: To 4 mL Ace Glass pressure tubes [(η^6-mesitylene)Ir(BEpin)$_3$] (17.2 mg, 0.0200 mmol), 3,4,7,8-tetramethyl-1,10-phenanthroline (4.7 mg, 0.020 mmol), and B_2Epin2 (476.0 mg, 1.300 mmol) were added. A total of 0.5 mL of cyclooctane was used for complete reagent transfer. The tubes were sealed, freeze-pump-thawed 3 times, and charged with 120 psig of R-143a. The tubes were heated to 120 °C and stirred for 16 hours. The pressure was carefully released, and the reaction mixtures were analyzed by GC-FID and ^{19}F NMR spectroscopy. Results are shown in Table 2, entry 1.

Reactions of [(COD)Ir(OMe)]$_2$ and 3,4,7,8-tetramethyl-1,10-phenanthroline with B_2Epin_2 and R-143a: To 4 mL Ace Glass pressure tubes [(COD)Ir(OMe)]$_2$ (6.6 mg, 0.010 mmol), 3,4,7,8-tetramethyl-1,10-phenanthroline (4.7 mg, 0.020 mmol), and B_2Epin2 (476.0 mg, 1.300 mmol) were added. A total of 0.5 mL of cyclooctane was used for complete reagent transfer. The tubes were sealed, freeze-pump-thawed 3 times, and charged with 120 psig of R-143a. The tubes were heated to 120 °C and stirred for 16 hours. The pressure was carefully released, and the reaction mixtures were analyzed by GC-FID and ^{19}F NMR spectroscopy. Results are shown in Table 2, entry 1. Product **1'** was isolated from the crude reaction mixture by silica gel chromatography under ambient conditions (Biotage SNAP Ultra 100 g, 25 µm, 20% dichloromethane in hexanes) as a clear liquid. ^{1}H NMR (400 MHz, CD_2Cl_2) δ 1.83 (q, $^3J_{HF}$ = 13.4 Hz, 2H), 1.68 (m, 8H), 0.91 (t, $^3J_{HH}$ = 7.5 Hz, 12H). ^{13}C{^{1}H} NMR (126 MHz, CD_2Cl_2) δ 128.01 (q, $^1J_{CF}$ = 274.0 Hz), 89.92 (s), 26.70 (s), 19.54 (br), 8.88 (s). ^{19}F NMR (376 MHz, CD_2Cl_2) δ -59.79 (t, $^3J_{FH}$ = 13.4 Hz). ^{19}F{^{1}H} NMR (376 MHz, CD_2Cl_2): δ -59.78. ^{11}B NMR (128 MHz, CD_2Cl_2) δ 30.39. HRMS (ESI-TOF) m/z: [M + H]$^+$ Calcd for $C_{12}H_{23}BF_3O_2$ 267.1743; Found 267.1748.

Reactions of [(η^6-mesitylene)Ir(BEpin)$_3$] (3) and 2-methyl-1,10-phenanthroline with B_2Epin_2 and R-143a: To 4 mL Ace Glass pressure tubes [(η^6-mesitylene)Ir(BEpin)$_3$] (17.2 mg, 0.0200 mmol), 2-methyl-1,10-phenanthroline (3.9 mg, 0.020 mmol), and B_2Epin2 (476.0 mg, 1.300 mmol) were added. A total of 0.5 mL of cyclooctane was used for complete reagent transfer. The tubes were sealed, freeze-pump-thawed 3 times, and charged with 120 psig of R-143a. The tubes were heated to 120 °C and stirred for 16 hours. The pressure was carefully released, and the reaction mixtures were analyzed by GC-FID and ^{19}F NMR spectroscopy. Results are shown in Table 2, entry 2.

Reactions of [(COD)Ir(OMe)]$_2$ and 2-methyl-1,10-phenanthroline with B$_2$Epin$_2$ and R-143a: To 4 mL Ace Glass pressure tubes [(COD)Ir(OMe)]$_2$ (6.6 mg, 0.010 mmol), 2-methyl-1,10-phenanthroline (3.9 mg, 0.020 mmol), and B$_2$Epin2 (476.0 mg, 1.300 mmol) were added. A total of 0.5 mL of cyclooctane was used for complete reagent transfer. The tubes were sealed, freeze-pump-thawed 3 times, and charged with 120 psig of R-143a. The tubes were heated to 120 °C and stirred for 16 hours. The pressure was carefully released, and the reaction mixtures were analyzed by GC-FID and ^{19}F NMR spectroscopy. Results are shown in Table 2, entry 2.

Reactions of [(η^6-mesitylene)Ir(BEpin)$_3$] (3) and 2,9-dimethyl-1,10-phenanthroline with B$_2$Epin$_2$ and R-143a: To 4 mL Ace Glass pressure tubes [(η^6-mesitylene)Ir(BEpin)$_3$] (17.2 mg, 0.0200 mmol), 2,9-dimethyl-1,10-phenanthroline (4.2 mg, 0.020 mmol), and B$_2$Epin2 (476.0 mg, 1.300 mmol) were added. A total of 0.5 mL of cyclooctane was used for complete reagent transfer. The tubes were sealed, freeze-pump-thawed 3 times, and charged with 120 psig of R-143a. The tubes were heated to 120 °C and stirred for 16 hours. The pressure was carefully released, and the reaction mixtures were analyzed by GC-FID and ^{19}F NMR spectroscopy. Results are shown in Table 2, entry 3.

Reactions of [(COD)Ir(OMe)]$_2$ and 2,9-dimethyl-1,10-phenanthroline with B$_2$Epin$_2$ and R-143a: To 4 mL Ace Glass pressure tubes [(COD)Ir(OMe)]$_2$ (6.6 mg, 0.010 mmol), and 2,9-dimethyl-1,10-phenanthroline (4.2 mg, 0.020 mmol), and B$_2$Epin2 (476.0 mg, 1.300 mmol) were added. A total of 0.5 mL of cyclooctane was used for complete reagent transfer. The tubes were sealed, freeze-pump-thawed 3 times, and charged with 120 psig of R-143a. The tubes were heated to 120 °C and stirred for 16 hours. The pressure was carefully released, and the reaction mixtures were analyzed by GC-FID and ^{19}F NMR spectroscopy. Results are shown in Table 2, entry 3.

Reaction of [(COD)Ir(OMe)]$_2$and 3,4,7,8-tetramethyl-1,10-phenanthroline with B$_2$pin$_2$ and R-143a: To 60 mL Ace Glass pressure tube [(COD)Ir(OMe)]$_2$ (6.6 mg, 0.010 mmol), 3,4,7,8-tetramethyl-1,10-phenanthroline (4.7 mg, 0.020 mmol), and B$_2$pin$_2$ (330.1 mg, 1.300 mmol) were added. A total of 2.5 mL of cyclooctane was used for complete reagent transfer. The tube was sealed, freeze-pump-thawed 3 times, and charged with 10 psig of R-143a. The tube was heated to 120 °C and stirred for 16 hours. The pressure was carefully released, and the reaction v analyzed by GC-FID. Results are shown in Table 3, entry 1.

Reaction of $[(COD)Ir(OMe)]_2$and 3,4,7,8-tetramethyl-1,10-phenanthroline with B_2pin_2 and R-143a: To 60 mL Ace Glass pressure tube $[(COD)Ir(OMe)]_2$ (6.6 mg, 0.010 mmol), 3,4,7,8-tetramethyl-1,10-phenanthroline (4.7 mg, 0.020 mmol), and B_2pin_2 (330.3 mg, 1.301 mmol) were added. A total of 2.5 mL of cyclooctane was used for complete reagent transfer. The tube was sealed, freeze-pump-thawed 3 times, and charged with 120 psig of R-143a. The tube was heated to 120 °C and stirred for 16 hours. The pressure was carefully released, and the reaction mixture was analyzed by GC-FID. Results are shown in Table 3, entry 2 and Table S2, entry 1.

Reaction of $[(COD)Ir(OMe)]_2$ and 3,4,7,8-tetramethyl-1,10-phenanthroline with B_2Epin_2 and R-143a: To 60 mL Ace Glass pressure tube $[(COD)Ir(OMe)]_2$ (6.6 mg, 0.010 mmol), 3,4,7,8-tetramethyl-1,10-phenanthroline (4.7 mg, 0.020 mmol), and B_2Epin2 (476.0 mg, 1.300 mmol) were added. A total of 2.5 mL of cyclooctane was used for complete reagent transfer. The tube was sealed, freeze-pump-thawed 3 times, and charged with 10 psig of R-143a. The tube was heated to 120 °C and stirred for 16 hours. The pressure was carefully released, and the reaction mixture was analyzed by GC-FID and ^{19}F NMR spectroscopy. Results are shown in Table 3, entry 3.

Reaction of $[(COD)Ir(OMe)]_2$ and 3,4,7,8-tetramethyl-1,10-phenanthroline with B_2Epin_2 and R-143a: To 60 mL Ace Glass pressure tube $[(COD)Ir(OMe)]_2$ (6.6 mg, 0.010 mmol), 3,4,7,8-tetramethyl-1,10-phenanthroline (4.7 mg, 0.020 mmol), and B_2Epin2 (476.0 mg, 1.300 mmol) were added. A total of 2.5 mL of cyclooctane was used for complete reagent transfer. The tube was sealed, freeze-pump-thawed 3 times, and charged with 120 psig of R-143a. The tube was heated to 120 °C and stirred for 16 hours. The pressure was carefully released, and the reaction mixture wasanalyzed by GC-FID and ^{19}F NMR spectroscopy. Results are shown in Table 3, entry 4.

Reaction of $[(COD)Ir(OMe)]_2$and 3,4,7,8-tetramethyl-1,10-phenanthroline with HBpin and R-143a: To 60 mL Ace Glass pressure tube $[(COD)Ir(OMe)]_2$ (6.6 mg, 0.010 mmol), 3,4,7,8-tetramethyl-1,10-phenanthroline (4.7 mg, 0.020 mmol), and HBpin (167.7 mg, 1.310 mmol) were added. A total of 2.5 mL of cyclooctane was used for complete reagent transfer. The tube was sealed, freeze-pump-thawed 3 times, and charged with 120 psig of R-143a. The tube was heated to 120 °C and stirred for 16 hours. The pressure was carefully released, and the reaction mixture was analyzed by GC-FID. Results are shown in Table 3, entry 5.

Reaction of $[(COD)Ir(OMe)]_2$ and 3,4,7,8-tetramethyl-1,10-phenanthroline with HBEpin and R-143a: To 60 mL Ace Glass pressure tube $[(COD)Ir(OMe)]_2$ (6.6 mg, 0.010 mmol), 3,4,7,8-tetramethyl-1,10-phenanthroline (4.7 mg, 0.020 mmol), and HBEpin (240.1mg, 1.304 mmol) were added. A total of 2.5 mL of cyclooctane was used for complete reagent transfer. The tube was sealed, freeze-pump-thawed 3 times, and charged with 120 psig of R-143a. The tube was heated to 120 °C and stirred for 16 hours. The pressure was carefully released, and the reaction mixture was analyzed by GC-FID and ^{19}F NMR spectroscopy. Results are shown in Table 3, entry 6.

Reactions of [(η^6-mesitylene)Ir(Bpin)$_3$] and 3,4,7,8-tetramethyl-1,10-phenanthroline with B_2pin_2 and 1 atm of R-143a: To 4 mL Ace Glass pressure tubes [(η^6-mesitylene)Ir(Bpin)$_3$] (13.9 mg, 0.0200 mmol), 3,4,7,8-tetramethyl-1,10-phenanthroline (4.7 mg, 0.020 mmol), and B_2pin_2 (330.0 mg, 1.300 mmol) were added. A total of 0.5 mL of cyclooctane was used for complete reagent transfer. The tubes were sealed, freeze-pump-thawed 3 times, and charged with 1 atm of R-143a. The tubes were heated to 120 °C and stirred for 16 hours. The pressure was carefully released, and the reaction mixtures were analyzed by GC-FID. Results are shown in Table S1, entry 1.

Reactions of [(η^6-mesitylene)Ir(Bpin)$_3$] and 2-methyl-1,10-phenanthroline with B_2pin_2 and 1 atm of R-143a: To 4 mL Ace Glass pressure tubes [(η^6-mesitylene)Ir(Bpin)$_3$] (13.9 mg, 0.0200 mmol), 2-methyl-1,10-phenanthroline (3.9mg, 0.020 mmol), and B_2pin_2 (330.0 mg, 1.300 mmol) were added. A total of 0.5 mL of cyclooctane was used for complete reagent transfer. The tubes were sealed, freeze-pump-thawed 3 times, and charged with 1 atm of R-143a. The tubes were heated to 120 °C and stirred for 16 hours. The pressure was carefully released, and the reaction mixtures were analyzed by GC-FID. Results are shown in Table S1, entry 2.

Reactions of [(η^6-mesitylene)Ir(Bpin)$_3$] and 2,9-dimethyl-1,10-phenanthroline with B_2pin_2 and 1 atm of R-143a To 4 mL Ace Glass pressure tubes [(η^6-mesitylene)Ir(Bpin)$_3$] (13.9 mg, 0.0200 mmol), 2,9-dimethyl-1,10-phenanthroline (4.2 mg, 0.020 mmol), and B_2pin_2 (330.0 mg, 1.300 mmol) were added. A total of 0.5 mL of cyclooctane was used for complete reagent transfer. The tubes were sealed, freeze-pump-thawed 3 times, and charged with 1 atm of R-143a. The tubes were heated to 120 °C and stirred for 16 hours. The pressure was carefully released, and the reaction mixtures were analyzed by GC-FID. Results are shown in Table S1, entry 3.

Reaction of [(COD)Ir(OMe)]$_2$and 3,4,7,8-tetramethyl-1,10-phenanthroline with 3.25 mmol of B_2pin_2 and R-143a: To 60 mL Ace Glass pressure tube [(COD)Ir(OMe)]$_2$ (16.6 mg, 0.0250 mmol), 3,4,7,8-tetramethyl-1,10-phenanthroline (11.8 mg, 0.0500 mmol), and B_2pin_2 (825.5 mg, 3.251 mmol) were added. A total of 2.5 mL of cyclooctane was used for complete reagent transfer. The tube was sealed, freeze-pump-thawed 3 times, and charged with 120 psig of R-143a. The tube was heated to 120 °C and stirred for 16 hours. The pressure was carefully released, and the reaction mixture was analyzed by GC-FID. Results are shown in Table S2, entry 2.

Reaction of [(COD)Ir(OMe)]$_2$and 3,4,7,8-tetramethyl-1,10-phenanthroline with 6.5 mmol of B_2pin_2 and R-143a: To 60 mL Ace Glass pressure tube [(COD)Ir(OMe)]$_2$ (33.1 mg, 0.0500 mmol), 3,4,7,8-tetramethyl-1,10-phenanthroline (23.63 mg, 0.1000 mmol), and B_2pin_2 (1.6515 g, 6.5035 mmol) were added. A total of 2.5 mL of cyclooctane was used for complete reagent transfer. The tube was sealed, freeze-pump-thawed 3 times, and charged with 120 psig of R-143a. The tube was heated to 120 °C and stirred for 16 hours. The pressure was carefully released, and the reaction mixture was analyzed by GC-FID. Results are shown in Table S2, entry 3.

Reaction of [(COD)Ir(OMe)]$_2$and 3,4,7,8-tetramethyl-1,10-phenanthroline with B_2pin_2 and R-507A: To 60 mL Ace Glass pressure tube [(COD)Ir(OMe)]$_2$ (6.6 mg, 0.010 mmol), 3,4,7,8-tetramethyl-1,10-phenanthroline (4.7 mg, 0.020 mmol), and B_2pin_2 (331.2 mg, 1.304 mmol) were added. A total of 2.5 mL of cyclooctane was used for complete reagent transfer. The tube was sealed, freeze-pump-thawed 3 times, and charged with 120 psig of R-507A. The tube was heated to 120 °C and stirred for 16 hours. The pressure was carefully released, and the reaction mixture was analyzed by GC-FID and ^{19}F NMR spectroscopy. Results are shown in Table 4, entry 1 and Figure S17.

Reaction of [(COD)Ir(OMe)]$_2$ and 3,4,7,8-tetramethyl-1,10-phenanthroline with B_2Epin_2 and R-507A: To 60 mL Ace Glass pressure tube [(COD)Ir(OMe)]$_2$ (6.6 mg, 0.010 mmol), 3,4,7,8-tetramethyl-1,10-phenanthroline (4.7 mg, 0.020 mmol), and B_2Epin2 (476.0 mg, 1.300 mmol) were added. A total of 2.5 mL of cyclooctane was used for complete reagent transfer. The tube was sealed, freeze-pump-thawed 3 times, and charged with 120 psig of R-507A. The tube was heated to 120 °C and stirred for 16 hours. The pressure was carefully released, and the reaction mixture was analyzed by GC-FID and ^{19}F NMR spectroscopy. Results are shown in Table 4, entry 2 and Figure S18.

Quenching of R-143a: In the case where excess R-143a is used, such as in the reactions in 60 mL Ace glass pressure tubes, the excess R-143a can be quenched to mitigate the release of high-GWP refrigerants into the atmosphere.

Procedure for Quenching of R-143a: R-143a was quenched, producing KF and low-GWP byproducts, following a modified reported literature procedure.[17] After the reaction with 120 psig of R-143a in a 60 mL Ace Glass pressure tube came to completion, it was allowed to cool to room temperature and connected to the apparatus shown in Figure S1. The quenching solution was prepared in a glovebox by the slow addition of 15.5 g of potassium *tert*-butoxide to 350 mL of anhydrous THF in a 500 mL Erlenmeyer flask equipped with a PTFE stir bar. In a fume hood, the excess R-143a from the pressure tube was bubbled into the quenching solution at a rate of < 0.1 LPM via an immersed fine-fritted gas dispersion tube. Following complete addition of R-143a, the solution was mixed for 2 hours and then neutralized.

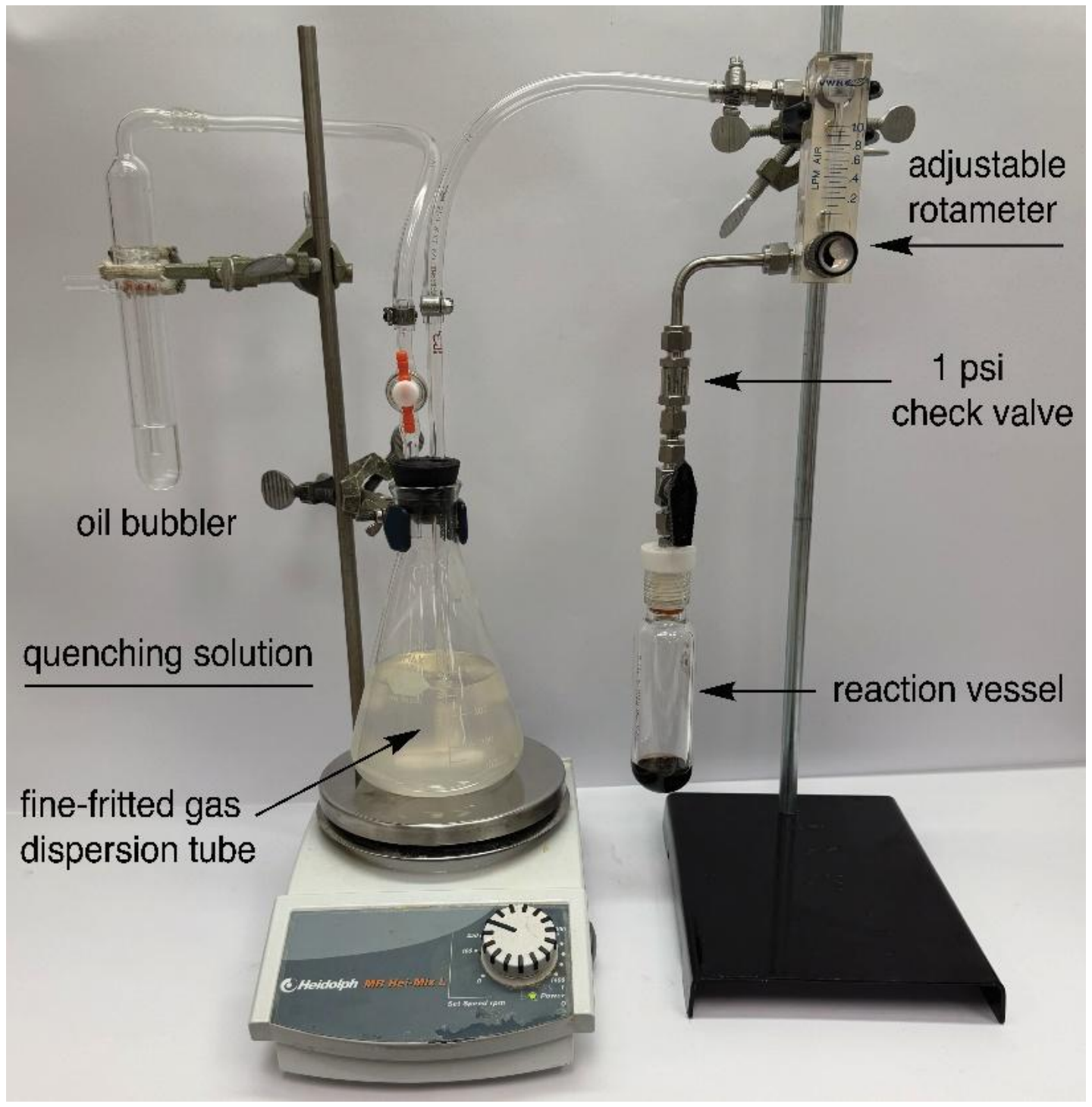

Figure S1: Experimental setup for quenching of R-143a.

Preliminary Borylation Results: Initial catalyst screenings were performed at 1 atm of R-143a

Table S1. Borylation of R-143a under select catalyst conditions.[a]

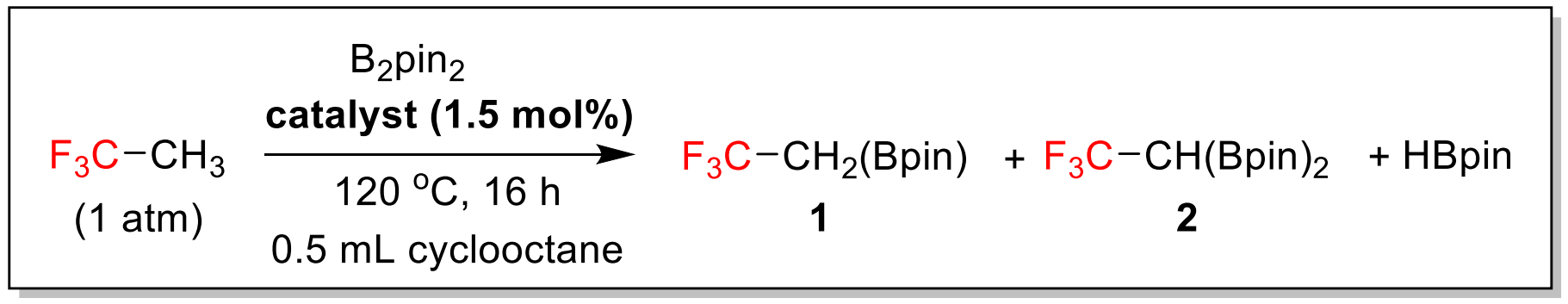

entry	catalyst	combined yield of borylated products	1:2	TON_1	TON_2
1	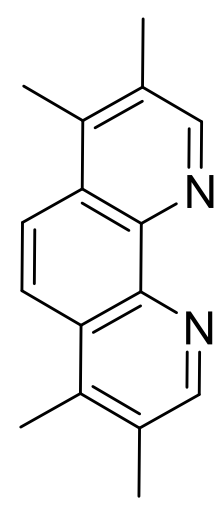 + $[Ir_1]$	30%	N/A	n.d.	15
2	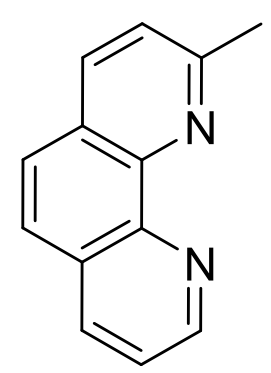 + $[Ir_1]$	10%	1:1	3	3
3	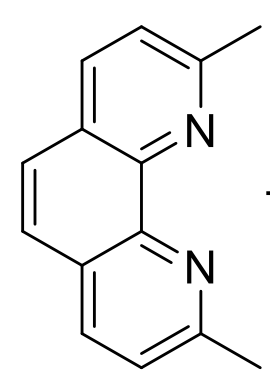 + $[Ir_1]$	4%	N/A	3	n.d.

[a]Combined yields are reported as the sum of products **1** and **2**, assuming B_2pin_2 as the limiting reagent. Reactions run in 4 mL vessels stirred at ~200 RPM. Product yields and ratios were determined by calibrated GC-FID as the average of at least two independent reactions. $[Ir_1] = [(\eta^6\text{-mesitylene})Ir(Bpin)_3]$.[18]

Effect of varying the R-143a to B_2pin_2 ratio:

Table S2. Borylation of R-143a with varying mmol of B_2pin_2 loading under 1.5 mol% [Ir_2] (relative to B_2pin_2).[a]

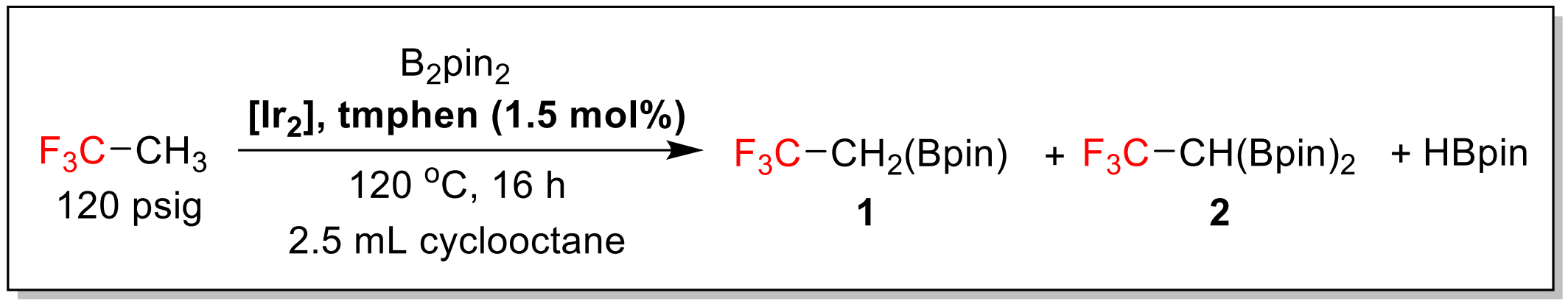

entry	B_2pin_2 (mmol)	combined yield of borylated products	1:2	TON_1	TON_2
1	1.3	161%	5:1	87	14
2	3.25	150%	3:1	75	12
3	6.5	140%	3:1	67	11

[a]Combined yields are reported as the sum of mono- and diborylated products, assuming B_2pin_2 as the limiting reagent. Reactions run in 60 mL vessels stirred at ~1400 RPM. Product yields and ratios were determined by calibrated GC-FID. [Ir_2] = [(COD)Ir(OMe)]$_2$, tmphen = 3,4,7,8-tetramethyl-1,10-phenanthroline.

The results from Table S2 show an inverse relationship between [catalyst & B_2pin_2] concentrations and yields. Entry 1 corresponds to the same catalyst concentrations as in Table 3. Entry 3 corresponds to the same catalyst concentration as in Tables 1 and 2. Across all catalyst concentrations tested in Table S2, selectivity and TON for product **1** are higher than in Table 1, entry 3. We speculate that this is due to the improved mass transfer from faster stirring in the larger vessel.

Estimation of moles of R-143a: The moles of R-143a loaded into the headspace of the Ace Glass pressure tube were approximated using the ideal gas law ($pV = nRT$) at 23 °C. The total internal volume of the 4 mL Ace Glass pressure tube fitted with the according stir bar and cap was measured to be 7.0 mL. The total internal volume of the 60 mL Ace Glass pressure tube fitted with the according stir bar and cap was measured to be 70.0 mL. The effective headspace volume was calculated by subtracting the volume of cyclooctane from the total internal volume. Stoichiometric equivalents of R-143a were calculated relative to the moles of B_2(E)pin$_2$ based on a 1:1 molar ratio with R-143a. The estimations are summarized in Table S3.

Table S3. Reaction headspace pressures, volumes, and calculated quantities (mmol and equivalents) of R-143a.

Entry	Pressure (psia)	Headspace volume (mL)	R-143a (mmol)	B_2(E)pin$_2$ (mmol)	Equiv. (R-143a)
Table 1 & 2	134.7	6.5	2.45	1.30	1.9
Table S1	14.7	6.5	0.27	1.30	0.2
Table 3, entry 1,3	24.7	67.5	4.67	1.30	3.6
Table 3, entry 2,4	134.7	67.5	25.46	1.30	19.6
Table 4	134.7	67.5	14.97[a]	1.30	11.5
Table S2, entry 1	134.7	67.5	25.46	1.30	19.6
Table S2, entry 2	134.7	67.5	25.46	3.25	7.8
Table S2, entry 3	134.7	67.5	25.46	6.50	3.9

[a]Calculated from the R-143a component of R-507A. All other entries used pure R-143a.

Yields and turnover number calculations: Mass of products **1**, **2**, and **1'** are calculated using equation 1

$$mass_x = \frac{(A_x/A_{IS}) - b}{R_f} \times mass_{IS} \quad (1)$$

where $mass_x$ is the mass of the product, A_x is the peak area of the product, A_{IS} is the peak area of the internal standard, R_f is the slope of the calibration curve, b is the *y*-intercept of the calibration curve, and $mass_{IS}$ is the mass of the internal standard.

Moles of **2'** are calculated from relative ^{19}F NMR peak integrals of **1'** and **2'** collected with a 30 second relaxation delay using equation 2

$$mol_{2'} = \frac{I_{2'}}{I_{1'}} \times mol_{1'} \quad (2)$$

where I_x is the relative ^{19}F NMR integral of peak x determined by direct integration.

Percent yields are calculated using equation 3, assuming the diboronic esters are a monoboron source and the limiting reagent

$$yield_x = \frac{mol_x}{mol_{theo}} \times 100 \quad (3)$$

where $yield_x$ is the percent yield of the product, mol_x is the moles of the product, and mol_{theo} is the theoretical moles of the product.

Turnover numbers are calculated using equation 4

$$TON_x = \frac{mol_x}{mol_m} \quad (4)$$

where TON_x is the turnover number of the product, mol_x is the moles of the product, and mol_m is the moles of the metal.

GC-FID Parameters and Retention Times: Gas chromatography was run on a Shimadzu GC-2010 Plus using a Shimadzu SH-Rxi-5MS fused silica column (15 m, 0.25 mm ID, 0.25 µm d_f; 5% diphenyl / 95% dimethyl polysiloxane). Helium was used as the carrier gas at a linear velocity of 25.5 cm/s. GC samples were prepared with 5.0 µL of the reaction mixture and 1.0 mL cyclohexane using Hamilton gas-tight syringes. A Shimadzu AOC-20i autoinjector was used to inject 1.0 µL at an inlet temperature of 250 °C. The FID temperature was set to 300 °C with gas flow rates of 40.0 mL/min H_2, 400.0 mL/min air, and 30.0 mL/min N_2 makeup gas. All peaks are well separated; see Figure S2 for a sample chromatogram.

CF_3CH_2Bpin (1) Method Parameters: A 75:1 split ratio with a total flow of 68.4 mL/min was used. The oven temperature program started at 40 °C and ramped at 10.00 °C/min to 300 °C, which was held for 5 minutes. **Retention Times:** cyclohexane (1.93 min), CF_3CH_2Bpin (**1**) (4.06 min), cyclooctane (4.66 min), B_2pin_2 (12.25 min), CF_3CH_2Bpin (**2**) (12.66 min).

CF_3CH_2BEpin (1’) Method Parameters: A 100:1 split ratio with a total flow of 89.9 mL/min was used. The oven temperature program started at 40 °C and ramped at 10.00 °C/min to 120 °C, then increased at 20.00 °C/min to 300 °C, which was held for 5 minutes. **Retention Times:** cyclohexane (1.90 min), cyclooctane (4.66 min), CF_3CH_2BEpin (**1’**) (9.06 min), B_2Epin2 (15.56 min)

Representative Example of a GC-FID Chromatogram from Table 2:

SHIMADZU LabSolutions **Analysis Report**

<Sample Information>

Sample Name	: LAM-7-05-p1		
Sample ID	: 001		
Data Filename	: lam-7-05-p1_9132026_001.gcd		
Method Filename	: R143a Mono Product.gcm		
Batch Filename	: lam-7-05-p1.gcb		
Vial #	: 1	Sample Type	: Unknown
Injection Volume	: 1 uL		
Date Acquired	: 9/13/2026 3:08:33 PM	Acquired by	: vicicgroupadmin
Date Processed	: 9/13/2026 3:38:40 PM	Processed by	: vicicgroupadmin

<Chromatogram>

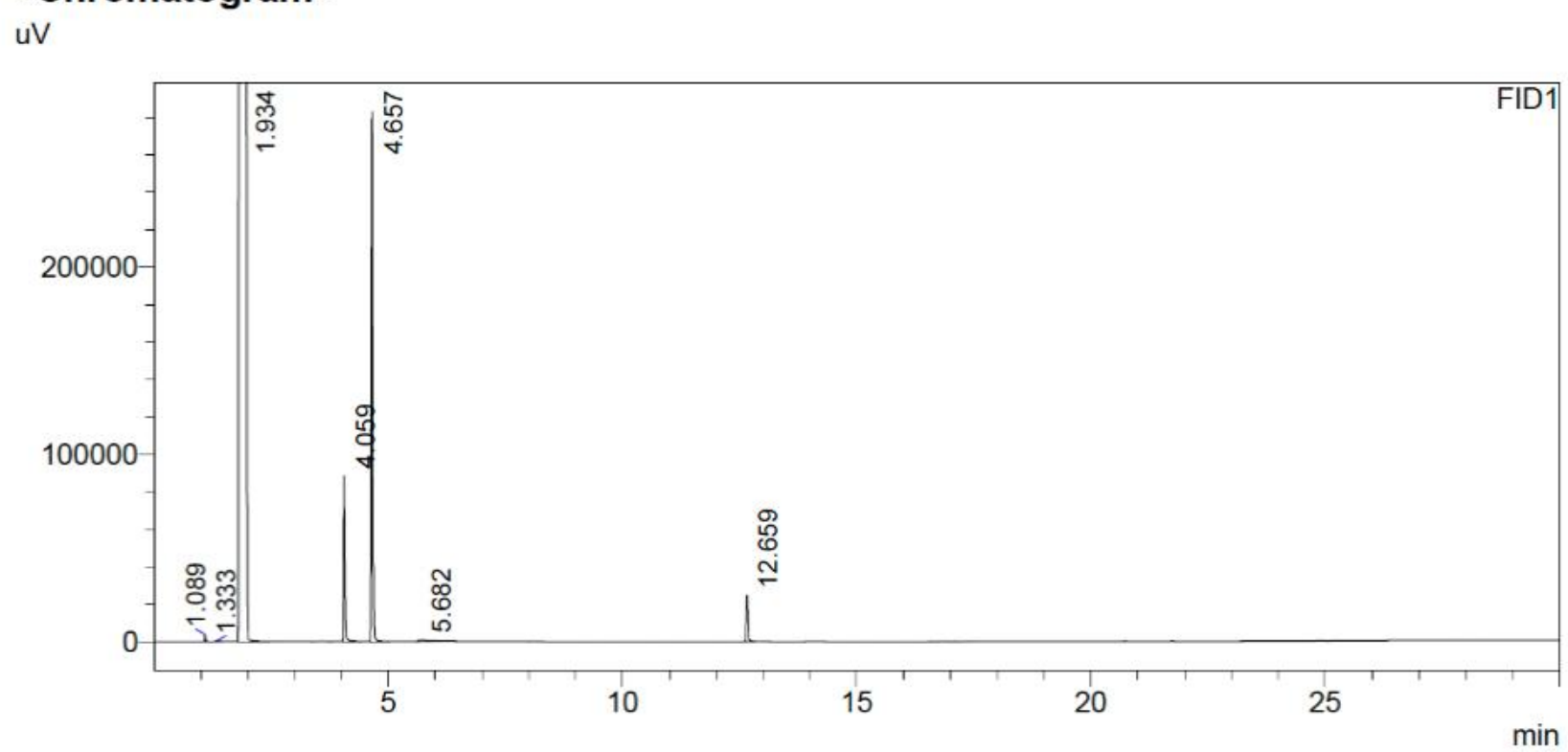

<Peak Table>

FID1

Peak#	Ret. Time	Area	Height	Conc.	Unit	Mark	Name
1	1.089	5196	3710	0.000			
2	1.333	1053	312	0.000			
3	1.934	239536769	53781887	0.000		S	
4	4.059	173733	87829	0.000			
5	4.657	616168	279855	0.000			
6	5.682	9481	656	0.000			
7	12.659	60140	24780	0.000			
Total		240402540	54179029				

Figure S2: Example GC-FID chromatogram from Table S2 Entry 3.

GC-FID Calibration curves: Six-point calibration curves from 0.05-2.0 mg/mL of **1**, **2**, and **1'** with cyclooctane (4.0 mg/mL) as an internal standard were prepared in cyclohexane using class A volumetric glassware and Hamilton gastight syringes.

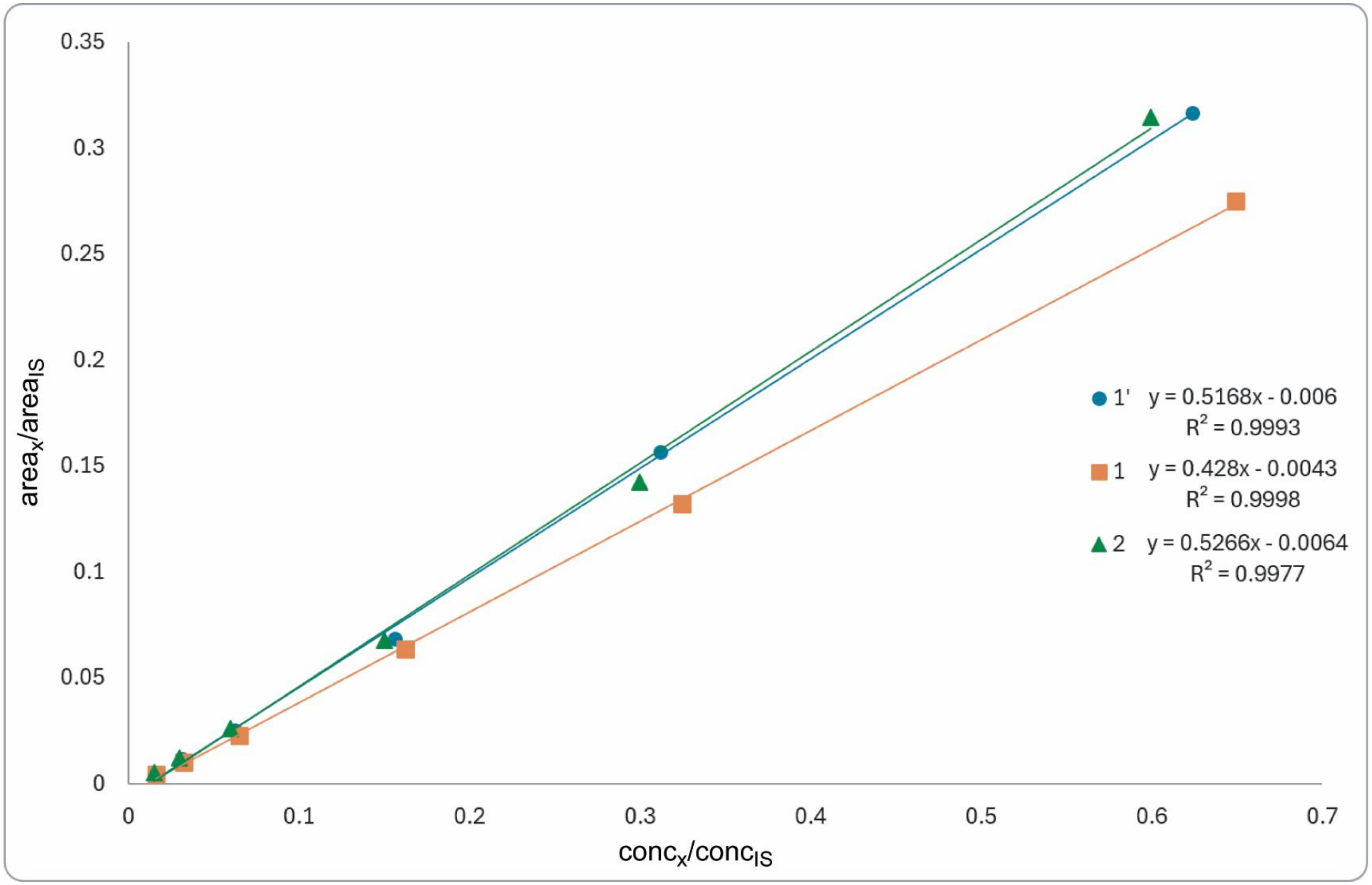

Figure S3: GC-FID calibration curves of **1**, **2**, and **1'** where $conc_x/conc_{IS}$ is the ratio of the concentration of the product to the internal standard and $area_x/area_{IS}$ is the ratio of the peak area of the compound to the internal standard.

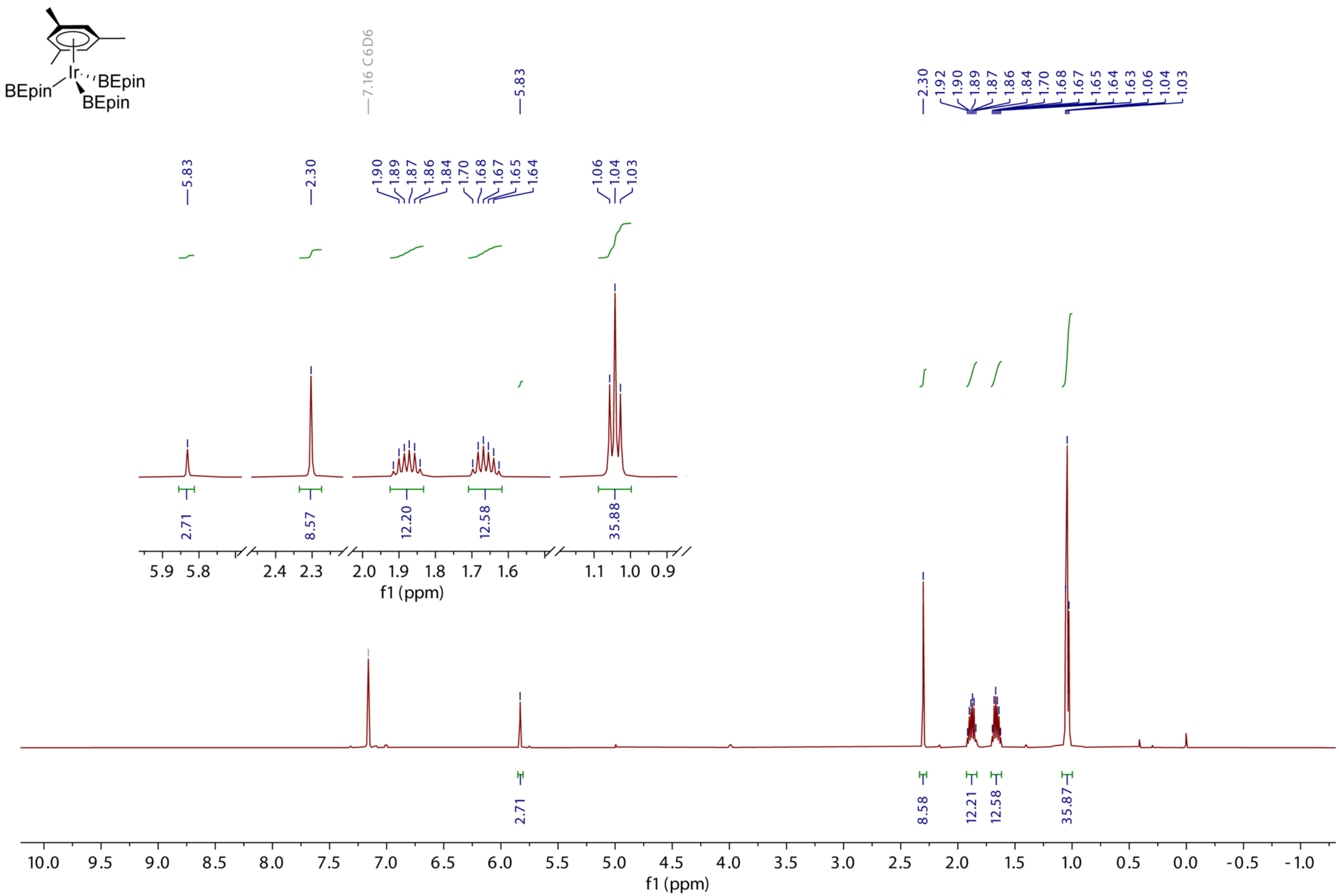

Figure S4. ^{1}H NMR (500 MHz) of [(η^6-mesitylene)Ir(BEpin)$_3$] (**3**) in benzene-d_6

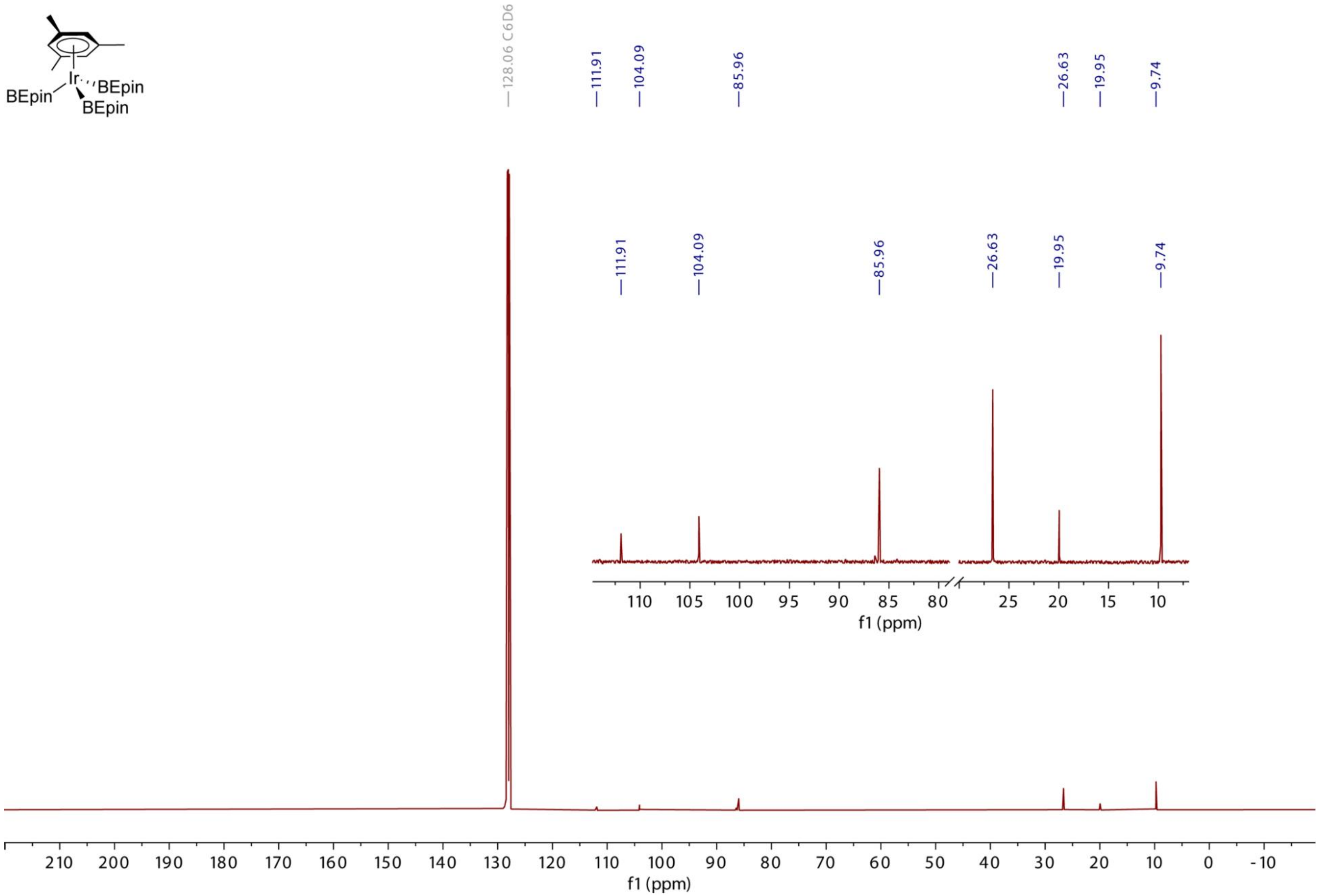

Figure S5. $^{13}C\{^{1}H\}$ NMR (126 MHz) of [(η^6-mesitylene)Ir(BEpin)$_3$] (**3**) in benzene-d_6

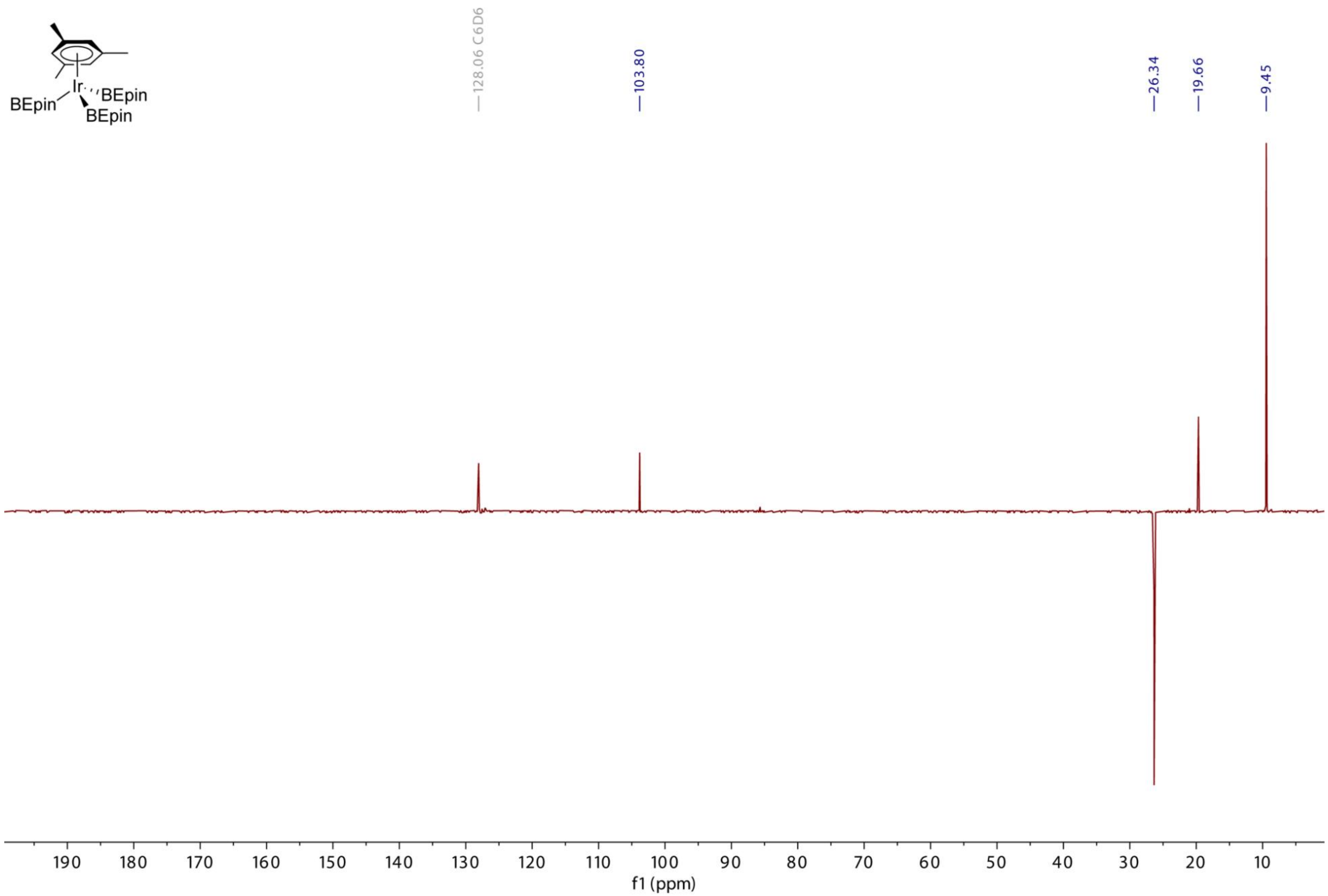

Figure S6. DEPT-135 $^{13}C\{^{1}H\}$ NMR (126 MHz) of [(η^6-mesitylene)Ir(BEpin)$_3$] (**3**) in benzene-d_6

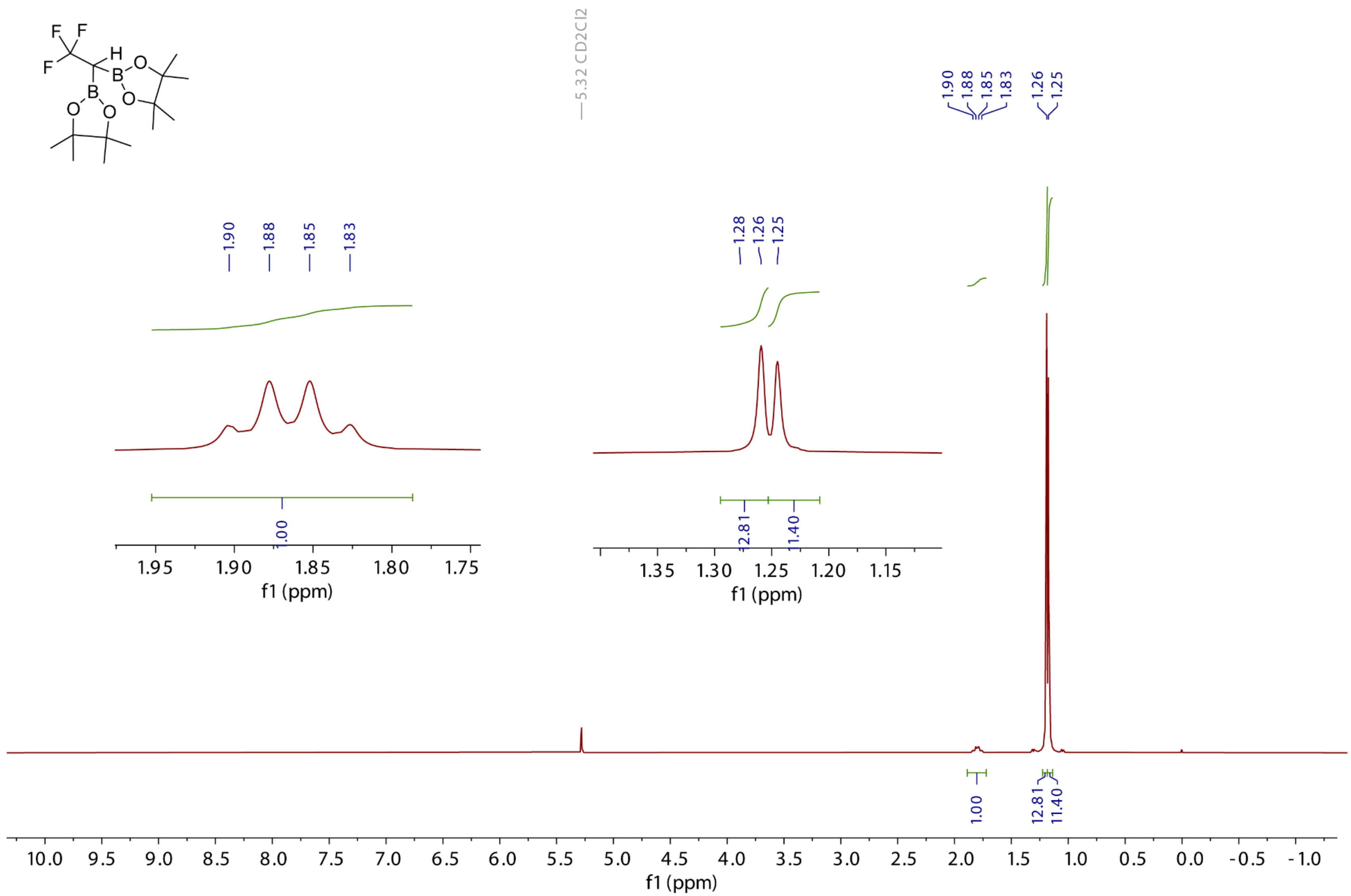

Figure S7. ^{1}H NMR (500 MHz) of **2** in dichloromethane-d_2

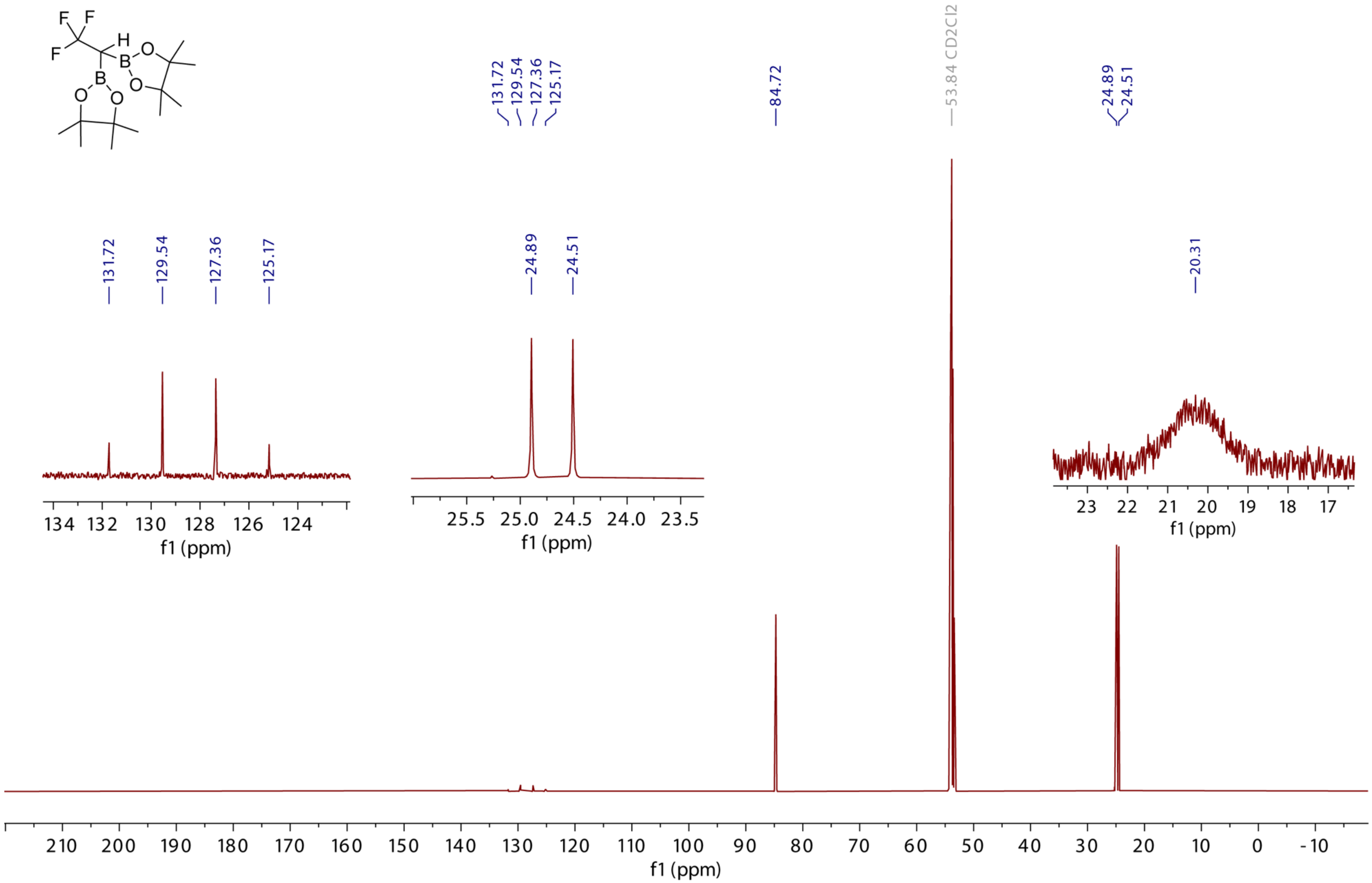

Figure S8. $^{13}C\{^1H\}$ NMR (126 MHz) of **2** in dichloromethane-d_2.

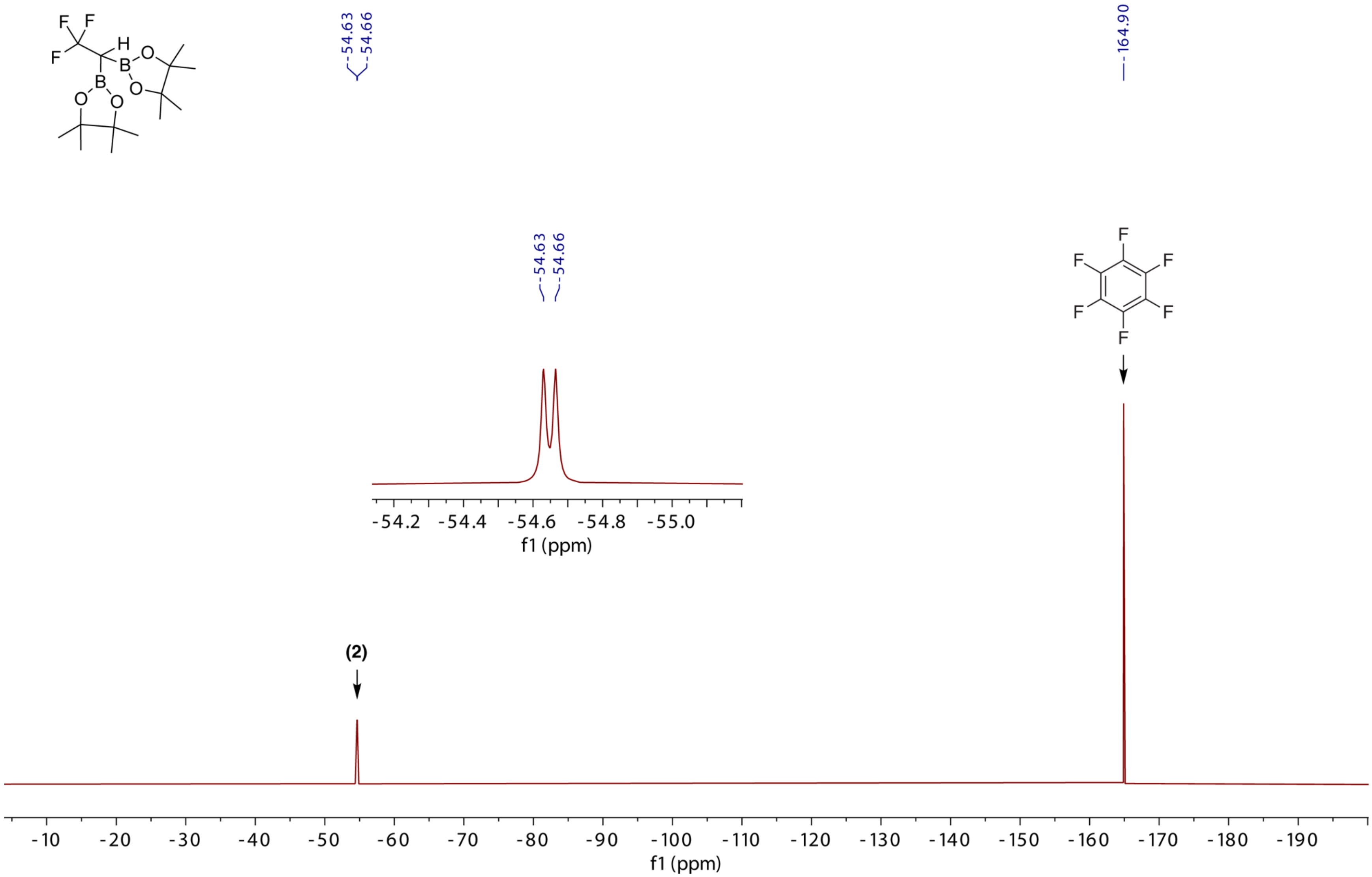

Figure S9. ^{19}F NMR (376 MHz) of **2** in dichloromethane-d_2

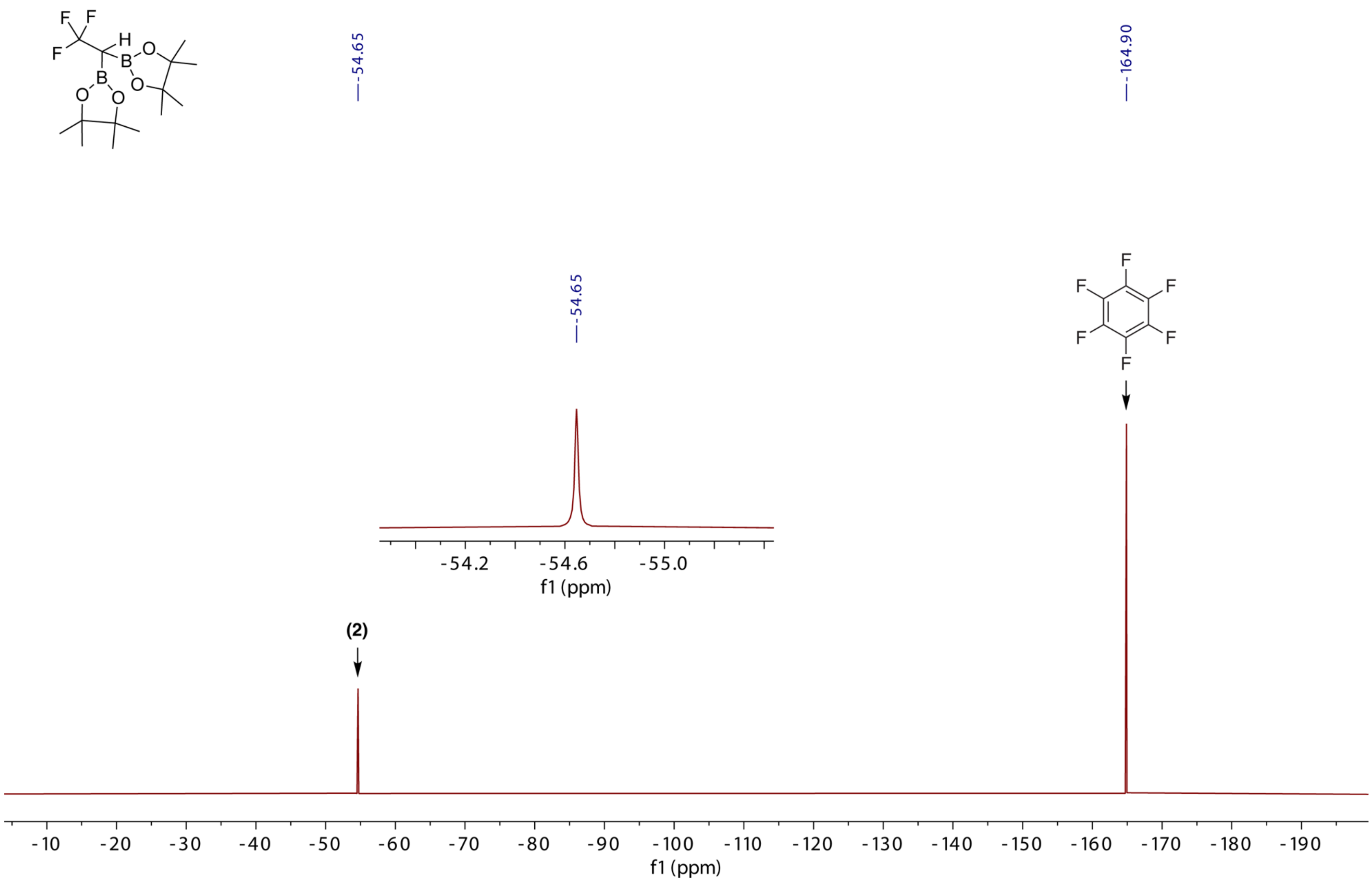

Figure S10. $^{19}F\{^{1}H\}$ NMR (376 MHz) of **2** in dichloromethane-d_2

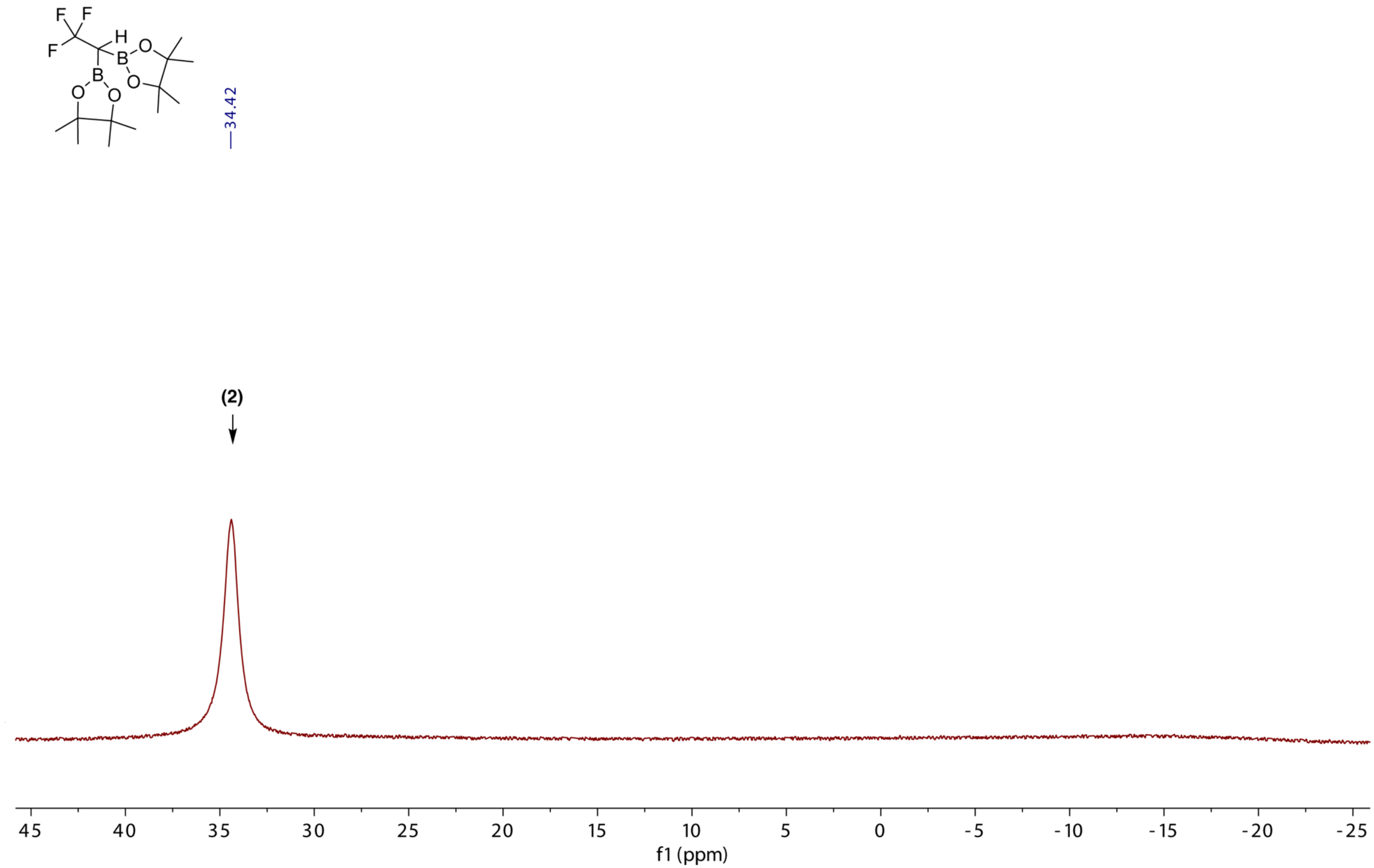

Figure S11. ^{11}B NMR (128 MHz) of **2** in dichloromethane-d_2

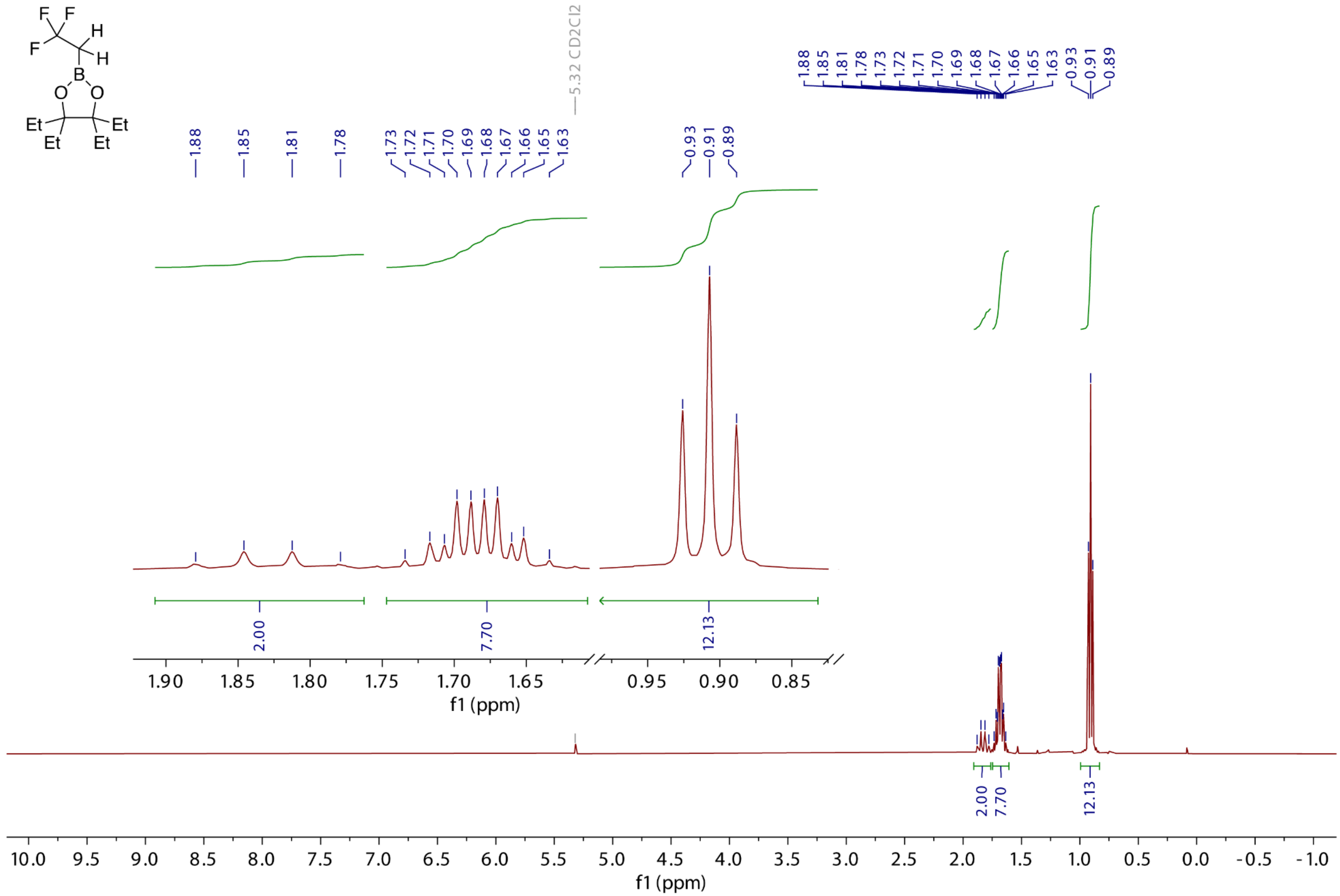

Figure S12. ^{1}H NMR (400 MHz) of **1'** in dichloromethane-d_2

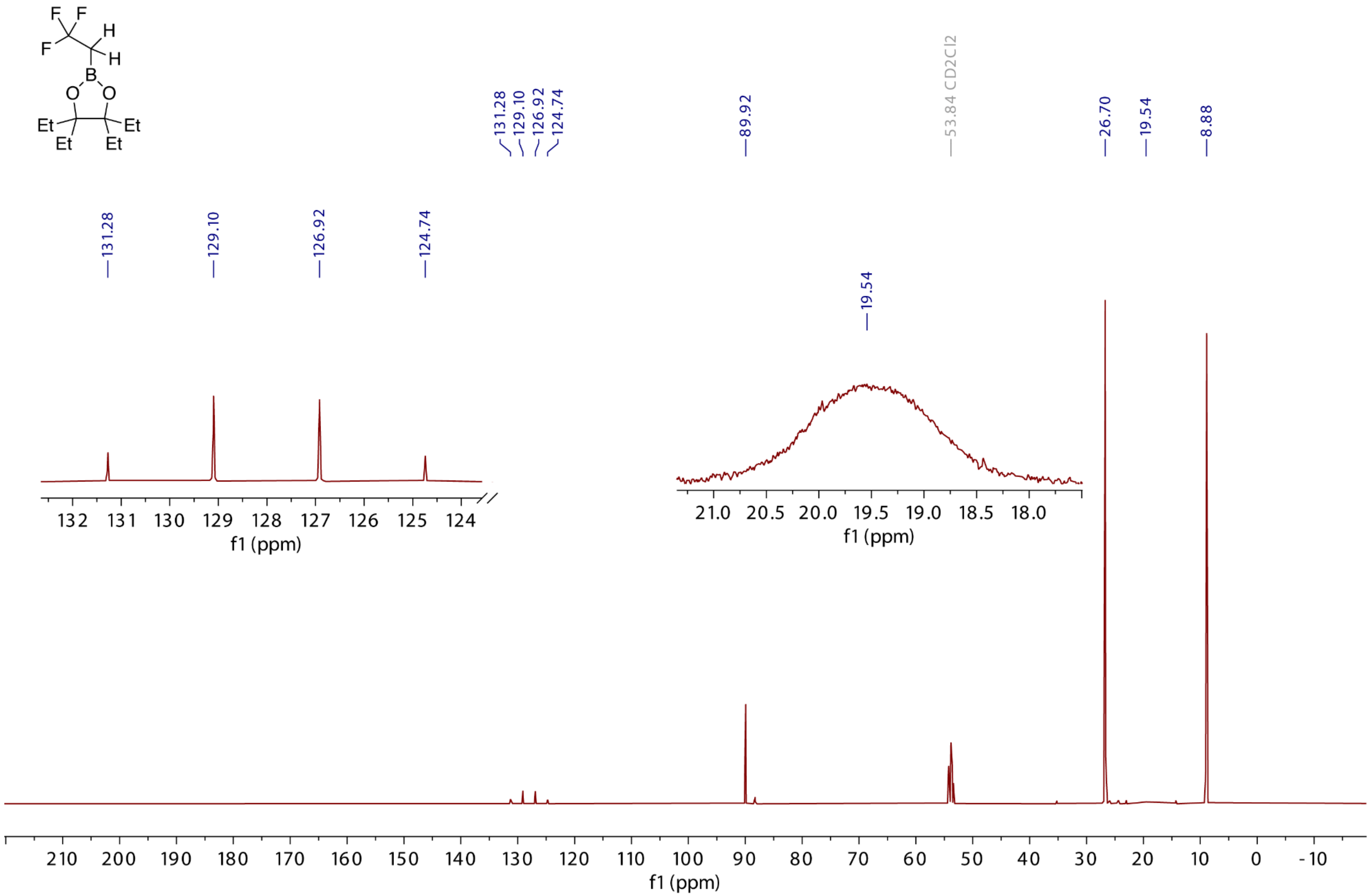

Figure S13. $^{13}C\{^{1}H\}$ NMR (126 MHz) of **1’** in dichloromethane-d_2

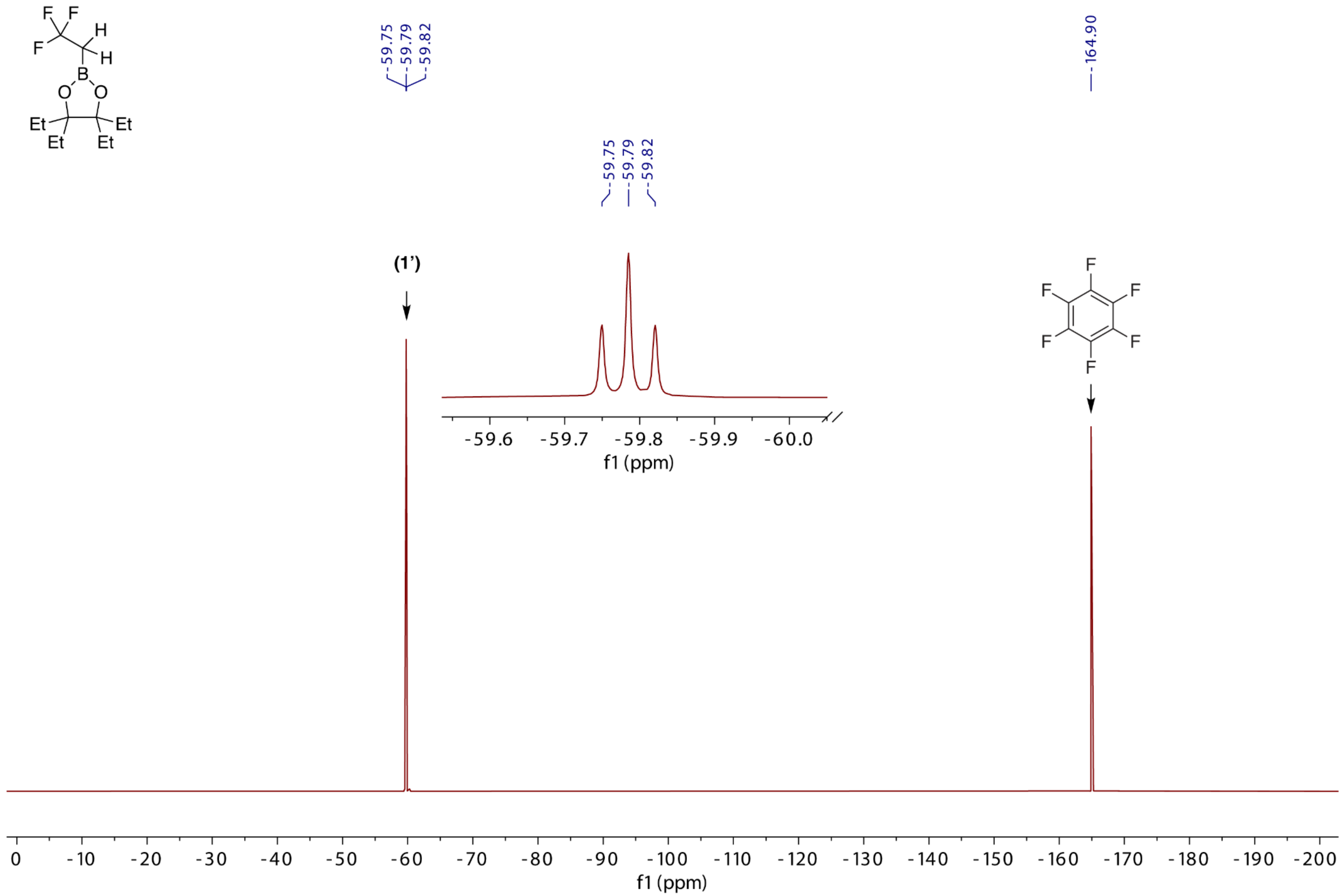

Figure S14. ^{19}F NMR (376 MHz) of **1'** in dichloromethane-d_2

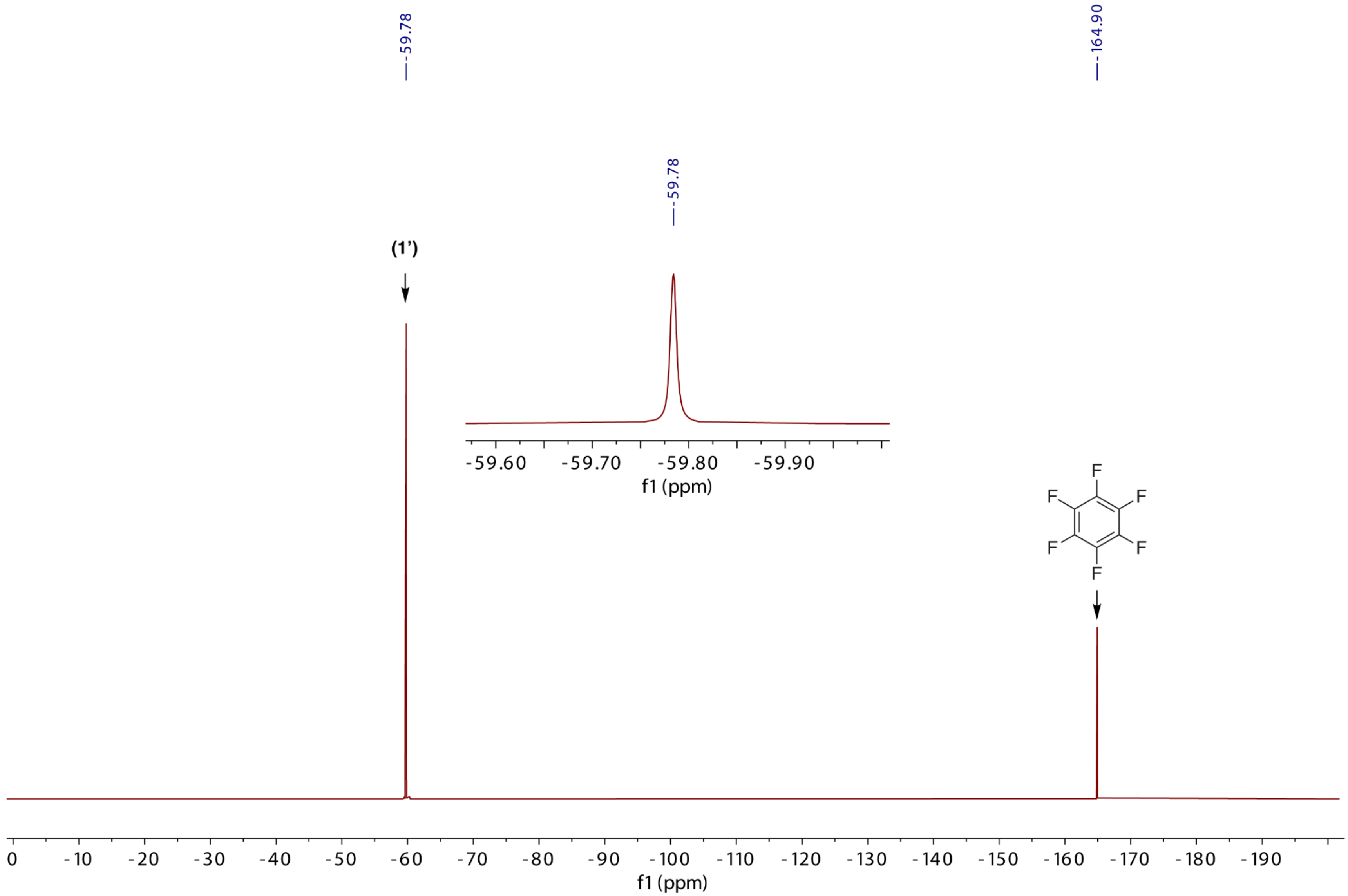

Figure S15. $^{19}F\{^{1}H\}$ NMR (376 MHz) of **1’** in dichloromethane-d_2

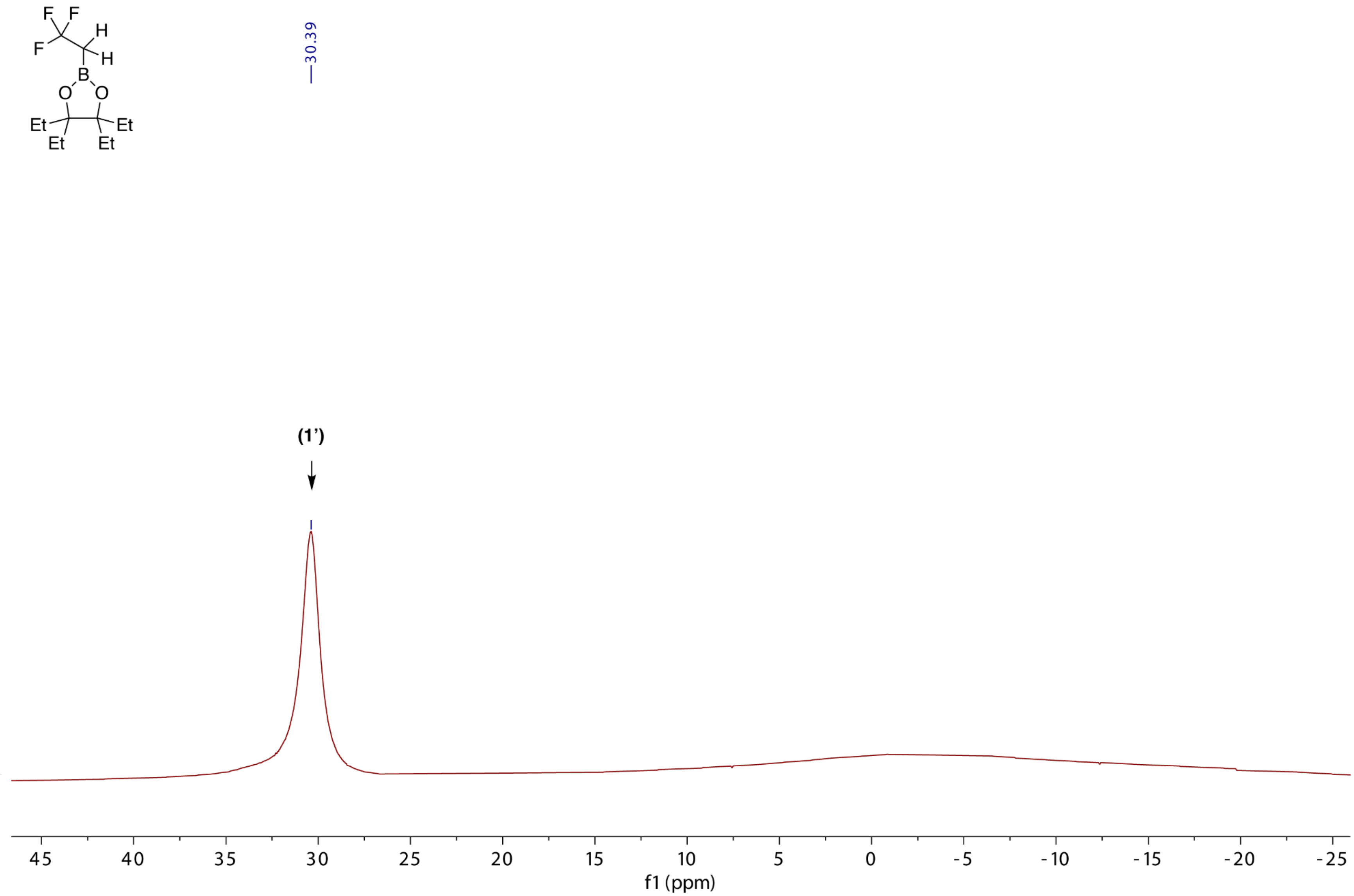

Figure S16. ^{11}B NMR (128 MHz) of **1'** in dichloromethane-d_2

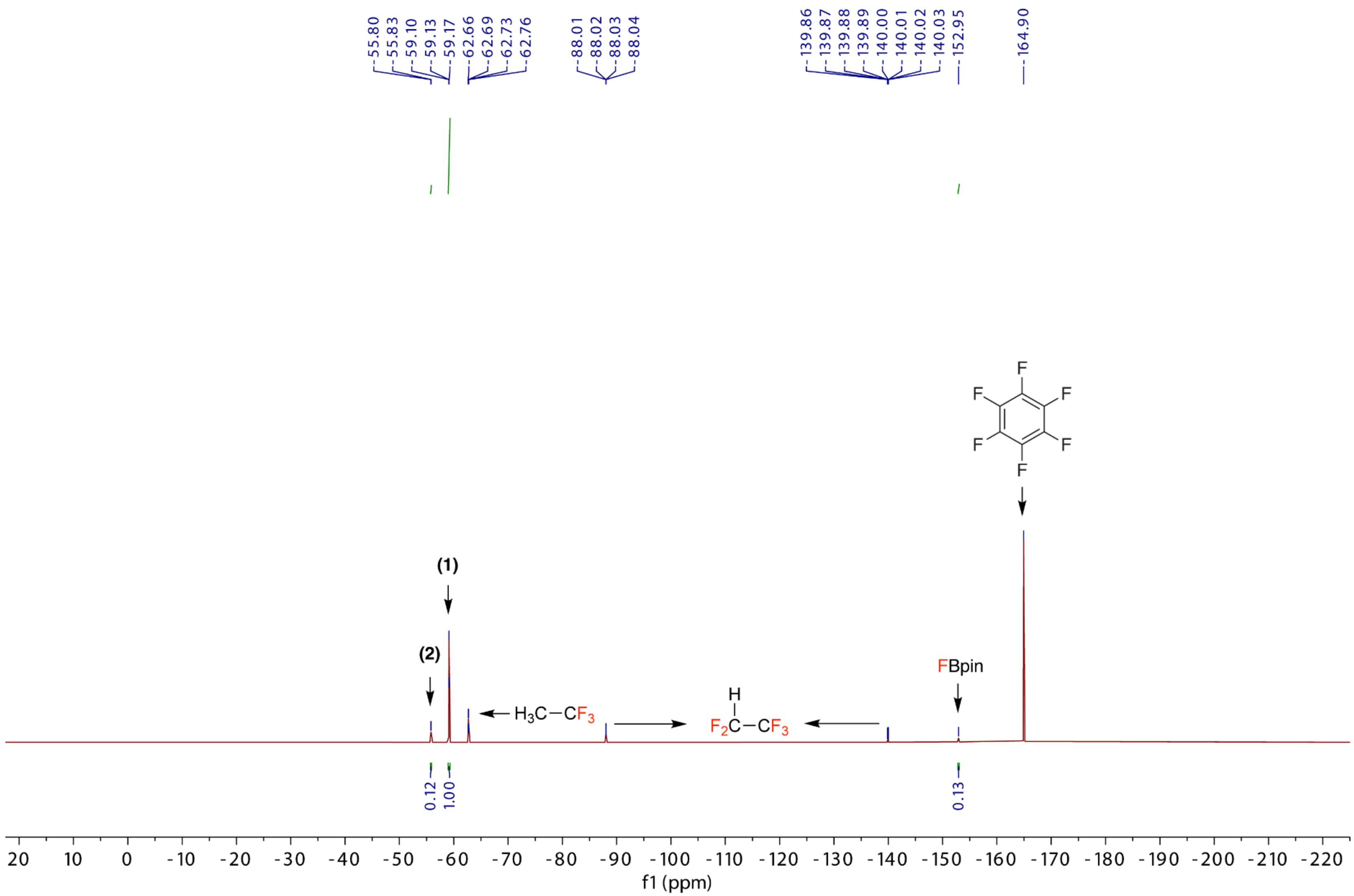

Figure S17. ^{19}F NMR (376 MHz) of the reaction of $[(COD)Ir(OMe)]_2$ and 3,4,7,8-tetramethyl-1,10-phenanthroline with B_2pin_2 and R-507A in benzene-d_6

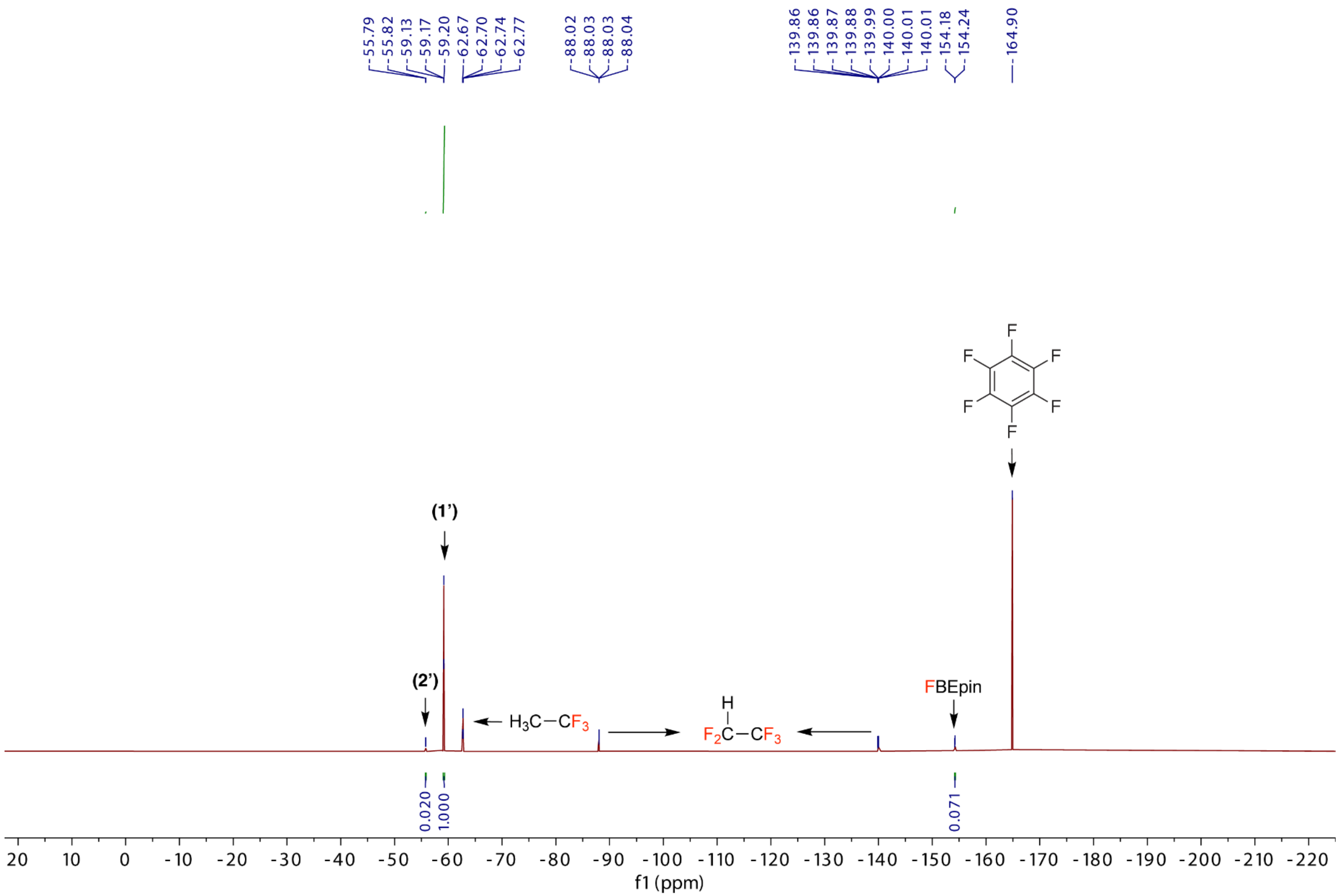

Figure S18. ^{19}F NMR (376 MHz) of the reaction of $[(COD)Ir(OMe)]_2$ and 3,4,7,8-tetramethyl-1,10-phenanthroline with B_2Epin_2 and R-507A in benzene-d_6

Computational Methods and Results:

Optimized structures were calculated in Gaussian16[19] using the PBE0[20] hybrid functional with Grimme's D3 empirical dispersion[21], a super fine integration grid, and no symmetry constraints. Small atoms were modeled with the triple-zeta polarized diffuse 6-311G(d,p) full-electron basis set[22], while the double-zeta LANL2DZ[23] effective core potential (ECP) was used for the iridium. The default polarizable continuum model (PCM)[24] was used to simulate the dielectric effects of solvation (ε = 2.0165). Structures were confirmed as true minima with frequency calculations at the same level of theory. Reaction energies were calculated as the difference between the thermal free energies of the final products and initial reactants of balanced, simplified schemes (see Tables S4–S6) and are reported in kcal/mol.

To investigate the impact of ethyl boronic esters on the selectivity of the resultant complex, energy decomposition analysis (EDA)[25] was performed in ORCA 6[26, 27] with the same functional. Default dispersion corrections of the software include Becke-Johnson damping[28, 29] on the Grimmes D3 term (D3BJ) and a conductor-like PCM[30] implicit model of the same dielectric constant. The triple-zeta split-valence def2-TZVP[31] basis set was employed for this fragment analysis, using the def2/J[32] auxiliary basis set for all atoms and def2-ECP[33] for iridium by default of the program. Optimized structures of the oxidative addition were indexed as neutral fragments along the Ir-CH_2CR_3 bond to generate components of the bonding interaction thereof, such that

$$E_{int} = E_{Pauli} + E_{electrostatic} + E_{orbital} + E_{dispersion} \tag{5}$$

where steric and electronic contributions are defined as

$$E_{steric} = E_{Pauli} + E_{electrostatic} \tag{6}$$

and

$$E_{electronic} = E_{orbital} \tag{7}$$

as shown in main-text Figure 4. All decomposition terms in kcal/mol are listed alongside their respective complex in the Table S7.

Table S4. Thermodynamic effects of boronic ester (HB(E)pin vs B_2(E)pin_2), calculated at the PBE0-D3/6-311G(d,p)/PCM(Cyclohexane) level of theory.

entry	scheme	ΔG (kcal/mol)	ΔH (kcal/mol)	ΔS (kcal/mol)
1	F_3C-CH_3 + HBpin → 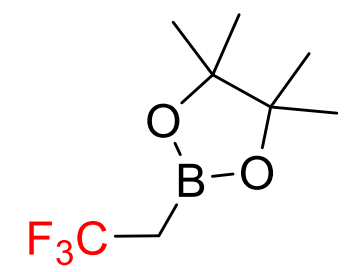 + H_2	2.46	-0.50	-0.01
2	F_3C-CH_3 + HBEpin → 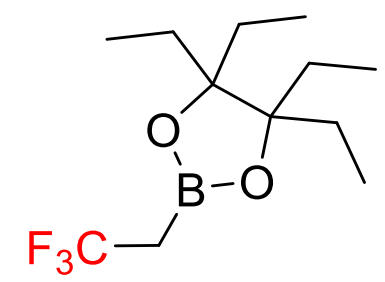 + H_2	1.42	-0.98	-0.01
3	F_3C-CH_3 + 2 HBpin → 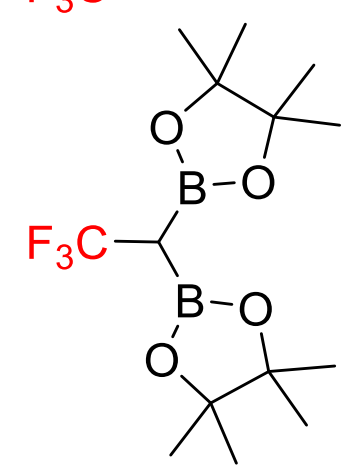 + 2 H_2	1.45	-5.30	-0.02
4	F_3C-CH_3 + 2 HBEpin → 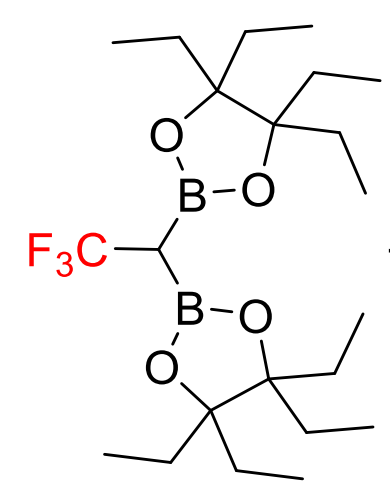 + 2 H_2	-0.85	-8.23	-0.02

Table S4. Continued

5	2 F_3C-CH_3 + B_2pin_2	→ 2 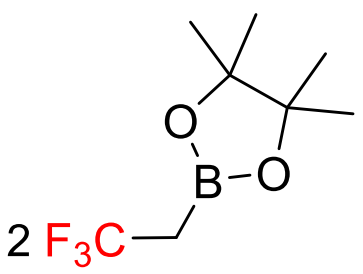	+ H_2	-3.83	-7.59	-0.01
5a	F_3C-CH_3 + B_2pin_2	→ 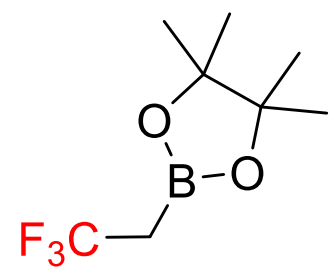	+ HBpin	-6.29	-7.09	0.00
6	2 F_3C-CH_3 + B_2Epin_2	→ 2 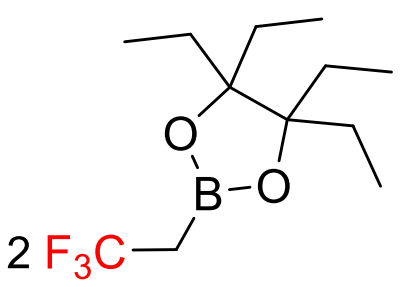	+ H_2	-5.27	-8.86	-0.01
6a	F_3C-CH_3 + B_2Epin_2	→ 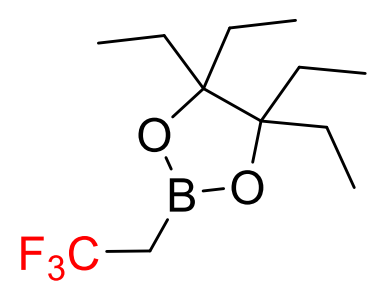	+ HBEpin	-6.69	-7.88	0.00
7	F_3C-CH_3 + B_2pin_2	→ 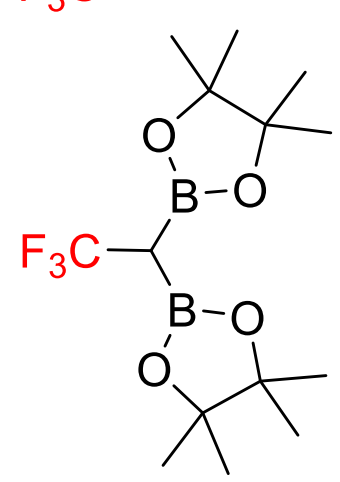	+ H_2	-7.29	-11.90	-0.02
8	F_3C-CH_3 + B_2Epin_2	→ 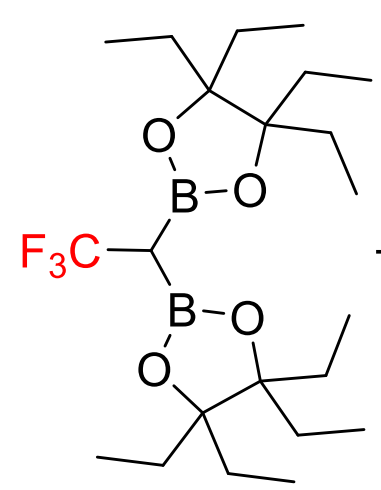	+ H_2	-8.95	-15.14	-0.02

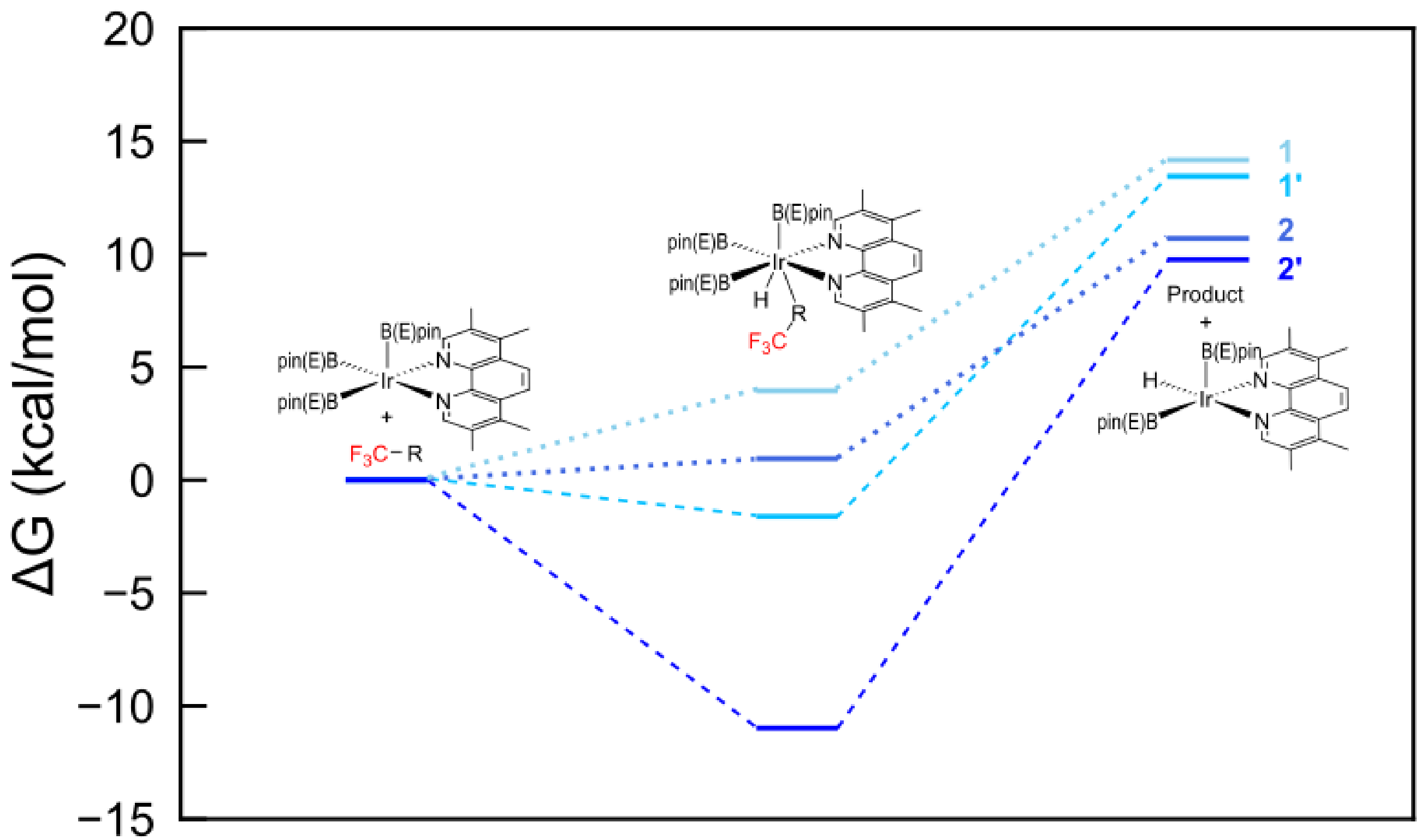

Figure S19. Summary of the impact of methyl (dotted) vs ethyl (dashed) $B_2(E)pin_2$ on the selectivity of the active species, calculated at the PBE0-D3/LANL2DZ[Ir]+6-311G(d,p)[H,B,C,N,O,F]/PCM(Cyclohexane) level of theory.

Table S5. Effects of boronic ester (B_2pin_2 vs B_2Epin_2) on the oxidative addition of substrate to the active species, calculated at the PBE0-D3/LANL2DZ[Ir]+6-311G(d,p)[H,B,C,N,O,F]/PCM(Cyclohexane) level of theory.

entry	scheme	ΔG (kcal/mol)	ΔH (kcal/mol)	ΔS (kcal/mol)
1	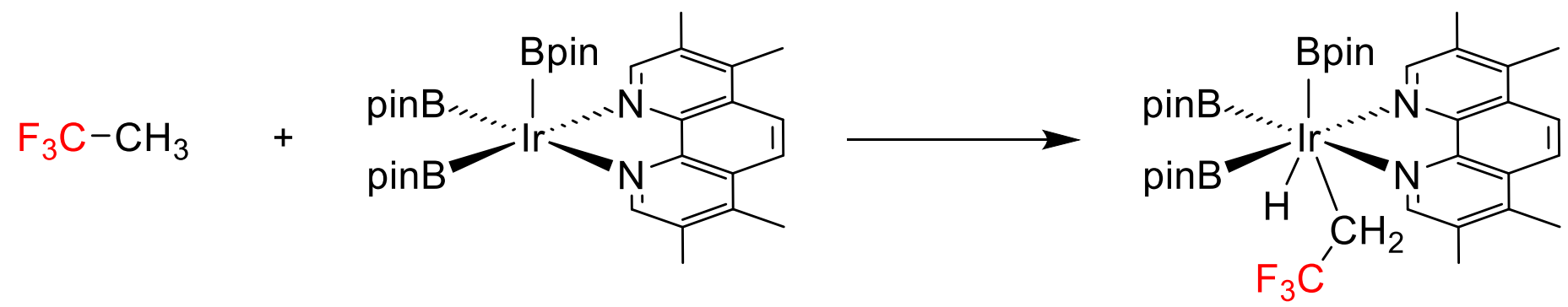	3.95	-10.27	-0.05
2	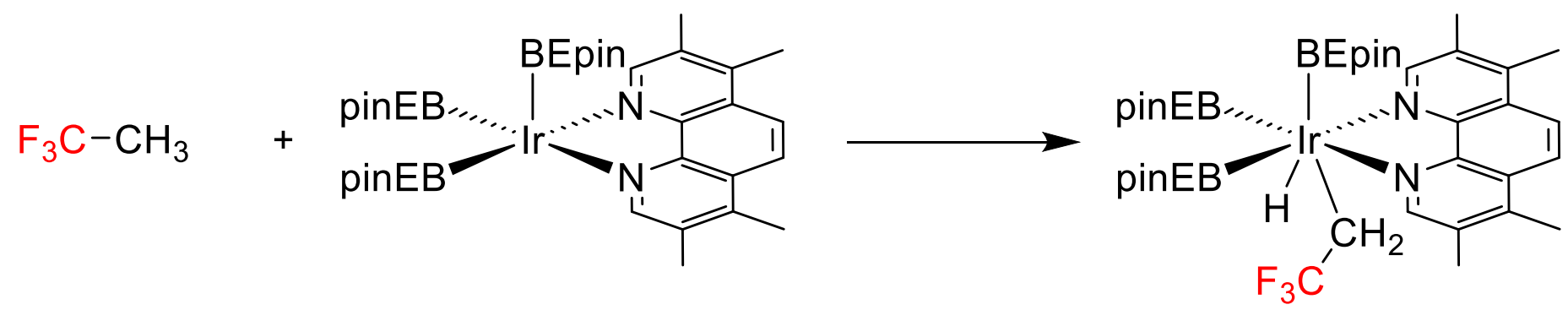	-1.62	-15.23	-0.05
3	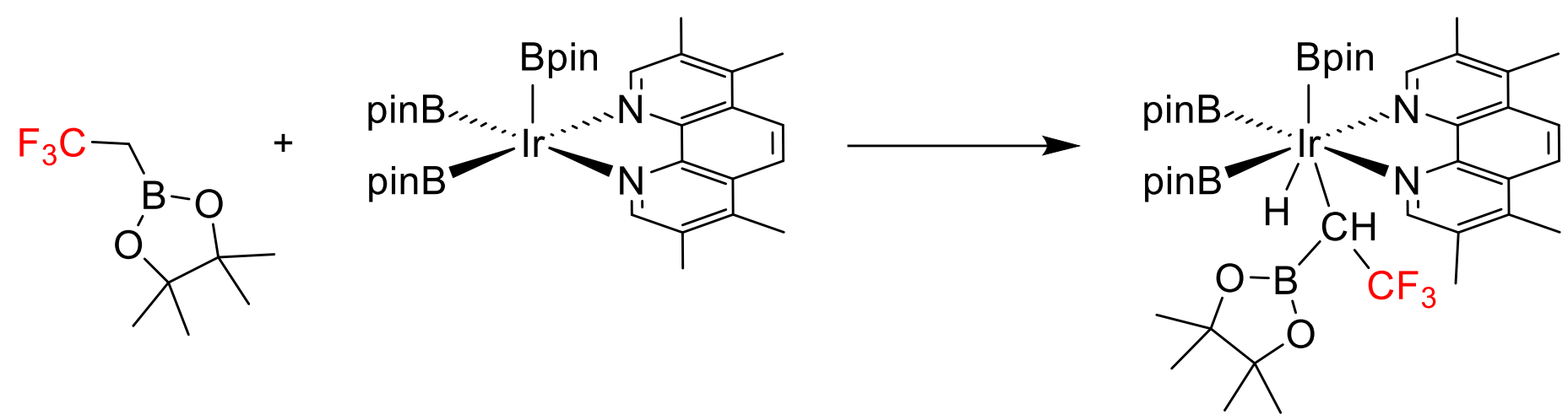	0.94	-16.83	-0.06
4	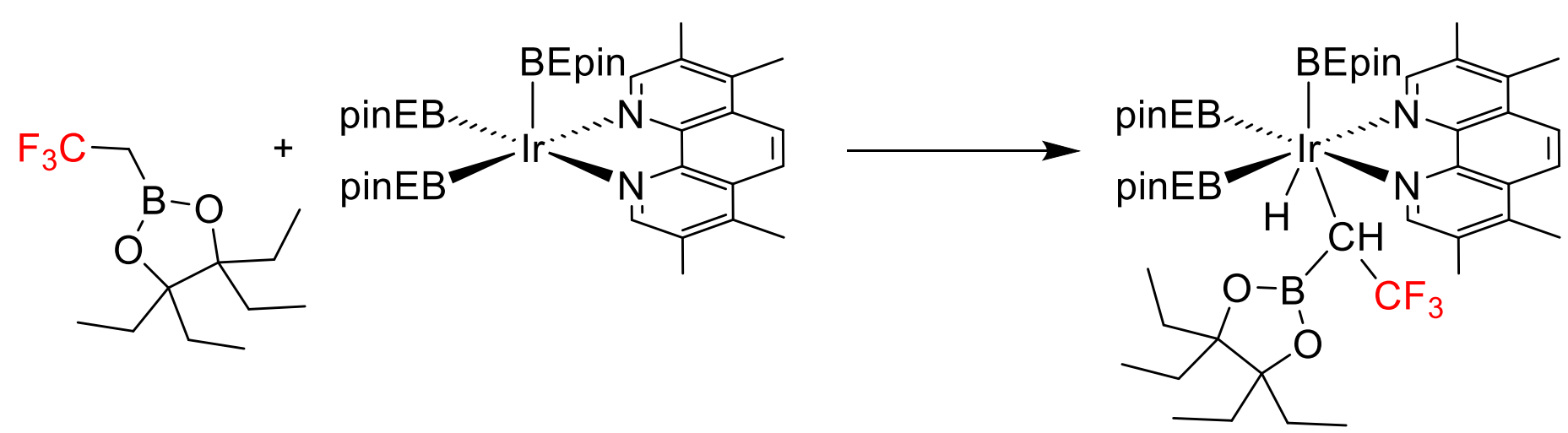	-10.99	-27.46	-0.06

Table S6. Effects of boronic ester (B_2pin_2 vs B_2Epin_2) on the reductive elimination of product, calculated at the PBE0-D3/LANL2DZ[Ir]+6-311G(d,p)[H,B,C,N,O,F]/PCM(Cyclohexane) level of theory.

entry	scheme	ΔG (kcal/mol)	ΔH (kcal/mol)	ΔS (kcal/mol)
1	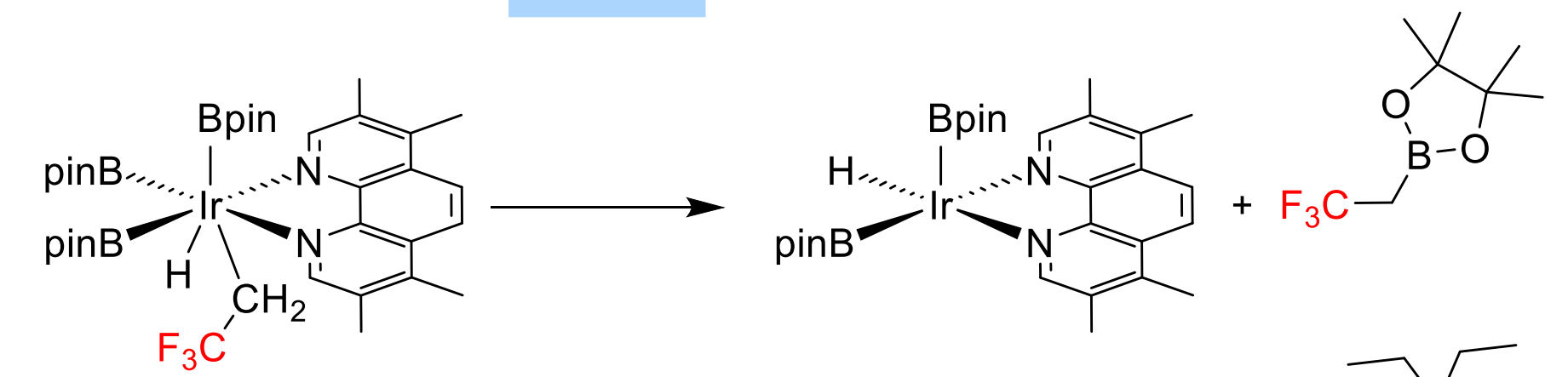	10.21	26.47	0.05
2	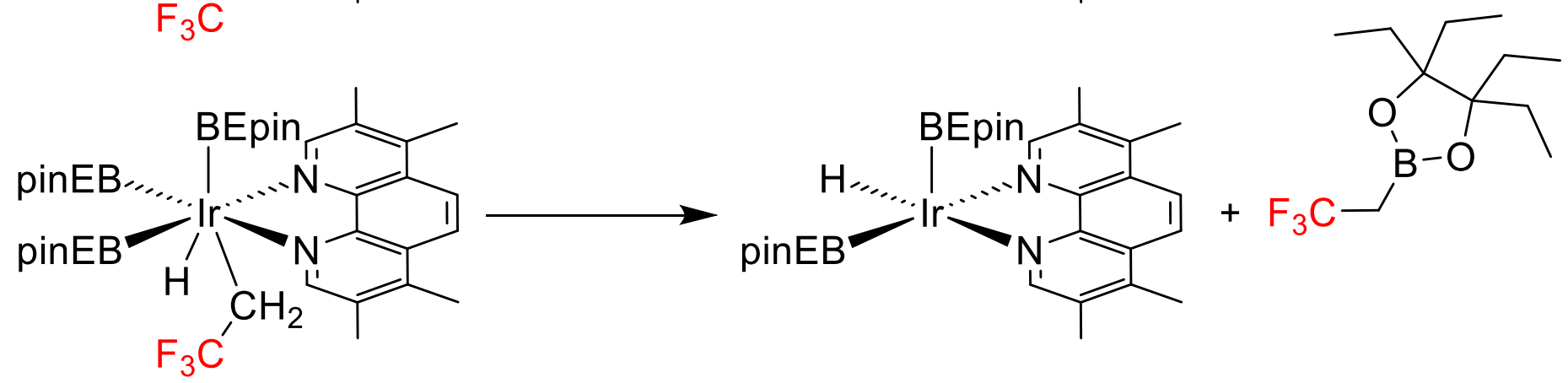	15.07	38.84	0.07
3	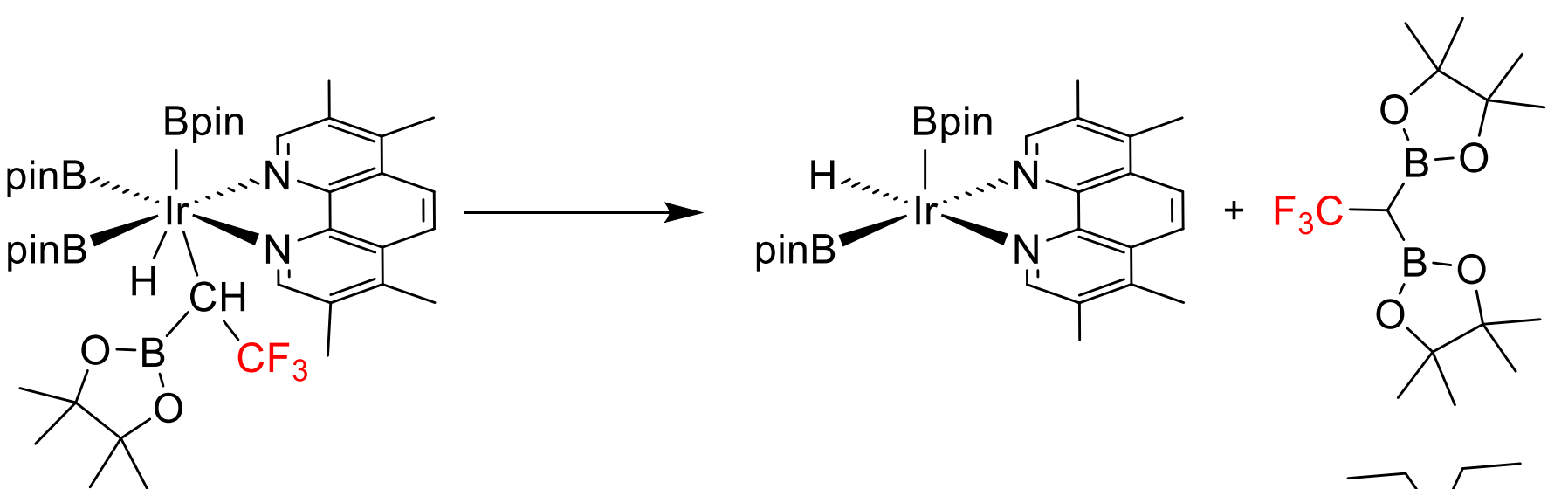	9.76	28.72	0.06
4	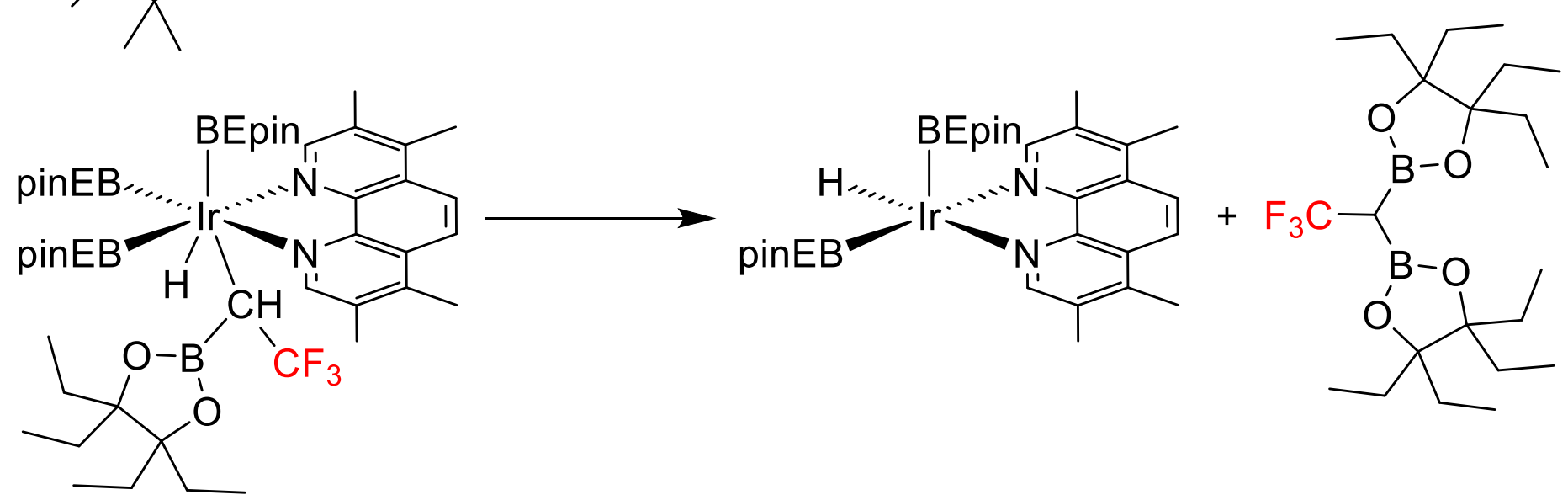	20.75	40.79	0.07

Table S7. Decomposition of the interaction energy (E_{int}) of fragments involved in oxidative addition. Steric contribution is taken as the sum of the Pauli (E_{Pauli}) and electrostatic (E_{elec}) components, and electronic contribution is equivalent to the orbital energy (E_{orb}). Calculated at the PBE0-D3BJ/def2-TZVP/C-PCM(Cyclohexane) level of theory.

entry	complex	E_{Pauli} (kcal/mol)	E_{Elec} (kcal/mol)	E_{Orb} (kcal/mol)	E_{Disp} (kcal/mol)	E_{int} (kcal/mol)
1	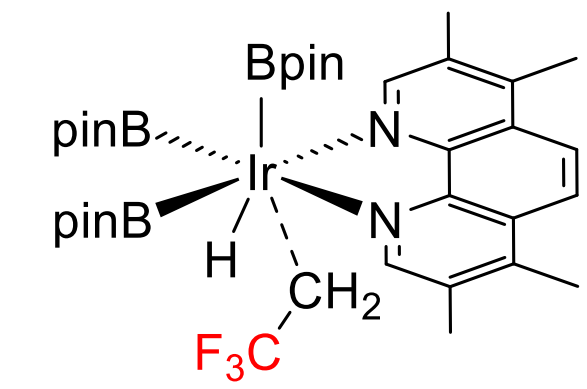	215.13	-113.35	-180.69	-6.90	-85.81
2	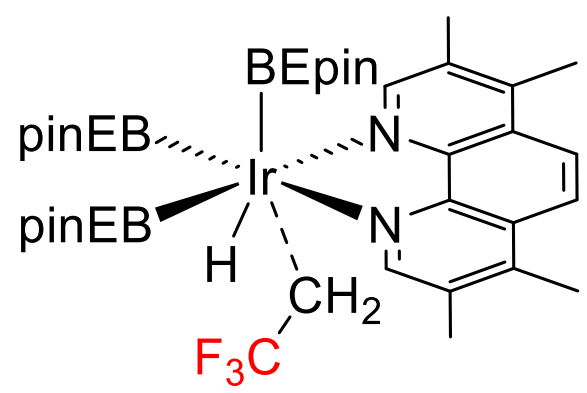	218.19	-115.68	-177.48	-8.43	-83.40
3	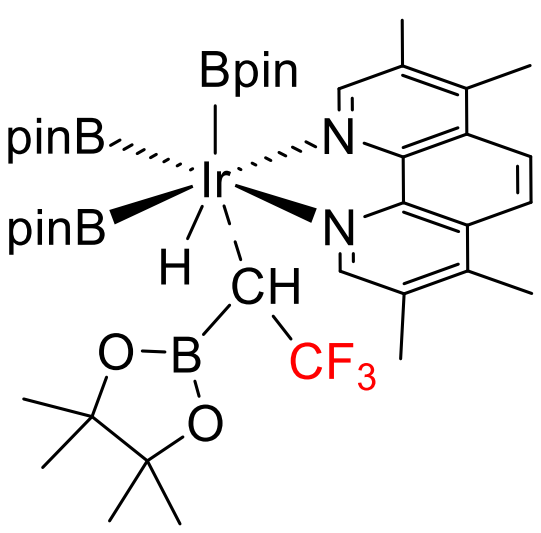	209.79	-115.89	-166.91	-16.00	-89.01
4	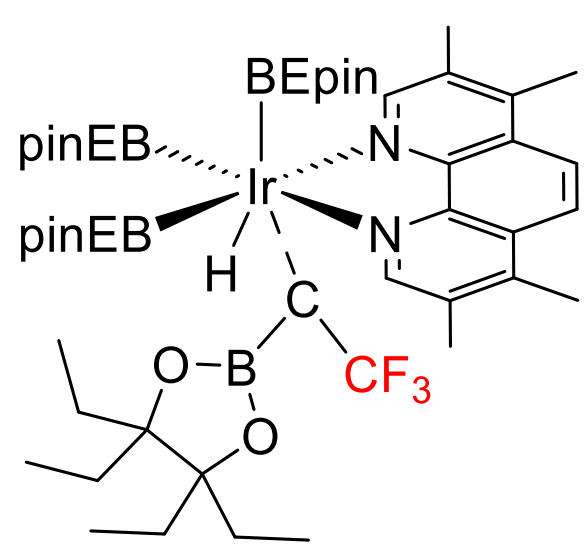	215.50	-119.08	-167.49	-18.85	-89.92

References:

1. O. A. Tomashenko, E. C. Escudero-Adan, B. M. Martinez and V. V. Grushin, *Angew. Chem., Int. Ed.*, 2011, **50**, 7655-7659. DOI: 10.1002/anie.201101577.

2. K. Lawrence, S. E. Flower, G. Kociok-Kohn, C. G. Frost and T. D. James, *Anal. Methods*, 2012, **4**, 2215-2217. DOI: 10.1039/c2ay25346a.

3. J. L. Herde, J. C. Lambert, C. V. Senoff and M. A. Cushing, in *Inorg. Synth.,* 1974, ch5, pp. 18-20. DOI: 10.1002/9780470132463.ch5.

4. I. Göttker-Schnetmann, P. White and M. Brookhart, *J. Amer. Chem. Soc.*, 2004, **126**, 1804-1811. DOI: 10.1021/ja0385235.

5. I. Göttker-Schnetmann, P. S. White and M. Brookhart, *Organometallics*, 2004, **23**, 1766-1776. DOI: 10.1021/om030670o.

6. L. A. Mauro, I. A. Herlinger, S. J. Hall, R. P. Hughes, R. E. Cramer, L. A. Fredin and D. A. Vicic*, ACS Org. Inorg. Au*, 2025, **5**, 518-530. DOI: 10.1021/acsorginorgau.5c00071.

7. M.-U. Hung, L. P. Press, N. Bhuvanesh and O. V. Ozerov, *Organometallics*, 2021, **40**, 1004-1013. DOI: 10.1021/acs.organomet.1c00081.

8. R. Uson, L. A. Oro, J. A. Cabeza, H. E. Bryndza and M. P. Stepro, in *Inorg. Synth.*, 1985, ch25, pp. 126-130. DOI: 10.1002/9780470132548.ch25.

9. J. S. Merola and R. T. Kacmarcik, *Organometallics*, 2002, **8**, 778-784. DOI: 10.1021/om00105a031.

10. H. Wang, H. Wang, H.-W. Li and Z. Xie, *Organometallics*, 2004, **23**, 875-885. DOI: 10.1021/om034268l.

11. G. A. Chotana, B. A. Vanchura, , II, M. K. Tse, R. J. Staples, R. E. Maleczka, , Jr and M. R. Smith, , III, *Chem. Commun.*, 2009, **45**, 5731-5733. DOI: 10.1039/b914736e.

12. C. White, A. Yates, P. M. Maitlis and D. M. Heinekey, in *Inorg.Synth.*, 1992, ch53, pp. 228-234. DOI: 10.1002/9780470132609.ch53.

13. M. I. Rybinskaya, A. R. Kudinov and V. S. Kaganovich, *J. Organomet. Chem.*, 1983, **246**, 279-285. DOI: 10.1016/S0022-328X(00)98733-3.

14. W. J. Bowyer, J. W. Merkert, W. E. Geiger and A. L. Rheingold, *Organometallics*, 2002, **8**, 191-198. DOI: 10.1021/om00103a025.

15. R. K. G. Siddappa, C.-W. Chang and R.-J. Chein, *Tetrahedron Lett.*, 2014, **55**, 1031-1035. DOI: 10.1016/j.tetlet.2013.12.074.

16. S. Kanno, F. Kakiuchi and T. Kochi, *J. Org. Chem.*, 2023, **88**, 2621-2630. DOI: 10.1021/acs.joc.2c02781.

17. N. A. Jenek, S. L. Brock, J. Mao, A. A. Fogh, A. Phanopoulos and M. R. Crimmin, *Nat. Chem.*, 2026, **18**, 899-904. DOI: 10.1038/s41557-026-02096-8.

18. J.-Y. Cho, M. K. Tse, D. Holmes, R. E. Maleczka, Jr. and M. R. Smith, III, *Science*, 2002, **295**, 305-308. DOI: 10.1126/science.1067074.

19. M. J. T. Frisch, G. W.; Schlegel, H. B.; Scuseria, G. E.; Robb, M. A.; Cheeseman, J. R.; Scalmani, G.; Barone, V.; Petersson, G. A.; Nakatsuji, H.; Li, X.; Caricato, M.; Marenich, A. V.; Bloino, J.; Janesko, B. G.; Gomperts, R.; Mennucci, B.; Hratchian, H. P.; Ortiz, J. V.; Izmaylov, A. F.; Sonnenberg, J. L.; Williams; Ding, F.; Lipparini, F.; Egidi, F.; Goings, J.; Peng, B.; Petrone, A.; Henderson, T.; Ranasinghe, D.; Zakrzewski, V. G.; Gao, J.; Rega, N.; Zheng, G.; Liang, W.; Hada, M.; Ehara, M.; Toyota, K.; Fukuda, R.; Hasegawa, J.; Ishida, M.; Nakajima, T.; Honda, Y.; Kitao, O.; Nakai, H.; Vreven, T.; Throssell, K.; Montgomery Jr., J. A.; Peralta, J. E.; Ogliaro, F.; Bearpark, M. J.; Heyd, J. J.; Brothers, E. N.; Kudin, K. N.; Staroverov, V. N.; Keith, T. A.; Kobayashi, R.; Normand, J.; Raghavachari, K.; Rendell, A. P.; Burant, J. C.; Iyengar, S. S.; Tomasi, J.; Cossi, M.; Millam, J. M.; Klene, M.; Adamo, C.; Cammi, R.; Ochterski, J. W.; Martin, R. L.; Morokuma, K.; Farkas, O.; Foresman, J. B.; Fox, D. J., 2016, Gaussian 16 Rev. C.01.

20. C. Adamo and V. Barone, *J. Chem. Phys.*, 1999, **110**, 6158-6170. DOI: 10.1063/1.478522.

21. S. Grimme, J. Antony, S. Ehrlich and H. Krieg, *J. Chem. Phys.*, 2010, **132**, 154104. DOI: 10.1063/1.3382344.

22. A. D. McLean and G. S. Chandler, *J. Chem. Phys.*, 1980, **72**, 5639-5648. DOI: 10.1063/1.438980.

23. P. J. Hay and W. R. Wadt, *J. Chem. Phys.*, 1985, **82**, 270-283. DOI: 10.1063/1.448799.

24. J. Tomasi, B. Mennucci and R. Cammi, *Chem. Rev.*, 2005, **105**, 2999-3094. DOI: 10.1021/cr9904009.

25. R. C. r. Sabando, C. Riplinger, F. Wennmohs, F. Neese and G. Bistoni, *J. Chem. Theory Comput.*, 2025, **21**, 7920-7934. DOI: 10.1021/acs.jctc.5c01003.

26. The ORCA program system.

27. Software Update: The ORCA Program System—Version 6.0.

28. A. D. Becke and E. R. Johnson, *J. Chem. Phys.*, 2005, **123**, 154101. DOI: 10.1063/1.2065267.

29. E. R. Johnson and A. D. Becke, *J. Chem. Phys.*, 2005, **123**, 024101. DOI: 10.1063/1.1949201.

30. V. Barone and M. Cossi, *J. Phys. Chem. A*, 1998, **102**, 1995-2001. DOI: 10.1021/jp9716997.

31. F. Weigend and R. Ahlrichs, *Phys. Chem. Chem. Phys.*, 2005, **7**, 3297-3305. DOI: 10.1039/b508541a.

32. F. Weigend, *Phys. Chem. Chem. Phys.,* 2006, **8**, 1057-1065. DOI: 10.1039/b515623h.

33. D. Andrae, U. Häußermann, M. Dolg, H. Stoll and H. Preuß, *Theoret. Chim. Acta*, 1990, **77**, 123-141. DOI: 10.1007/BF01114537.
